# Structural dissection of the catalytic domain of the serine threonine kinase StkP of *Streptococcus pneumoniae*

**DOI:** 10.1038/s41467-026-74470-6

**Published:** 2026-06-18

**Authors:** Mélisse Hamidi, Virginie Gueguen-Chaignon, Cassandra Falcou, Hugo Millat, Sathya Narayanan Nagarajan, Aline Le Roy, Céline Freton, Federico Gago, Stéphanie Ravaud, Christophe Grangeasse

**Affiliations:** 1 Université Lyon 1, CNRS, MMSB, UMR 5086, Lyon, France; 2 Protein Science Facility, CNRS UAR3444, INSERM US8, Université Claude Bernard, Lyon, France; 3 Univ. Grenoble Alpes, CNRS, CEA, IBS, Grenoble, France; 4 Department of Biomedical Sciences & Instituto de Química Médica-CSIC Associate Unit, School of Medicine and Health Sciences, University of Alcalá, Alcalá de Henares, Spain

**Keywords:** Bacterial physiology, Bacterial structural biology, Structural biology, Biophysics, Biochemistry

## Abstract

Serine/threonine kinases of the Hanks family are key regulators of bacterial physiology. Among them, membrane-associated PASTA-Hanks kinases govern bacterial cytokinesis and morphogenesis, yet their activation mechanism remains unclear. Here, we report crystal structures of the catalytic domain of the PASTA-Hanks kinase StkP of the human pathogen *Streptococcus pneumoniae*, carrying phosphoablative or phosphomimetic mutations in its activation loop. These structures demonstrate that phosphorylation of two threonine residues modulates the activation loop’s organization and dynamics and reveal an alternative mode of dimerization of the catalytic domain. Analytical ultracentrifugation, SAXS and cell imaging allow to propose a model postulating that the local concentration of StkP at the division septum promotes an inactive dimeric state in which the activation loop hampers substrate binding. The reorganization into active dimers would activate StkP and allow endogenous substrate phosphorylation. This work thus provides a mechanistic framework of the regulation of PASTA Hanks kinase for the regulation of bacterial cell division.

## Abstract (web summary)

PASTA-kinases govern the bacterial cell cycle. Here, authors solve the structure of the catalytic domain of the protein kinase StkP from Streptococcus pneumoniae and provide a mechanistic framework for its activation and regulation.

## Introduction

Protein phosphorylation is a fundamental process for signal sensing and transduction as well as for the regulation of numerous cellular processes in all living organisms^1^. In bacteria, years of intensive research have revealed the presence of a variety of protein kinases, some of which are only unique to bacteria^2–4^. Among them, the characterization of a protein kinase with a catalytic domain homologous to that of eukaryotic protein kinases was reported in 1991 in the bacterium *Myxococcus xanthus*^5^, more than 30 years after the identification of the first eukaryotic protein kinase^6^. Then, genome sequencing has rapidly revealed the widespread prevalence of these protein kinases among bacteria^7^. The vast majority of these “eukaryotic-like” protein kinases phosphorylate proteins on serine and threonine residues^8^. Given that serine/threonine kinases from bacteria and eukaryotes share a monophyletic origin^9^, these enzymes are grouped in the Hanks-type kinase family, in reference to Hanks and co-workers who originally described the structural features of these enzymes^10^.

**Fig. 1:**
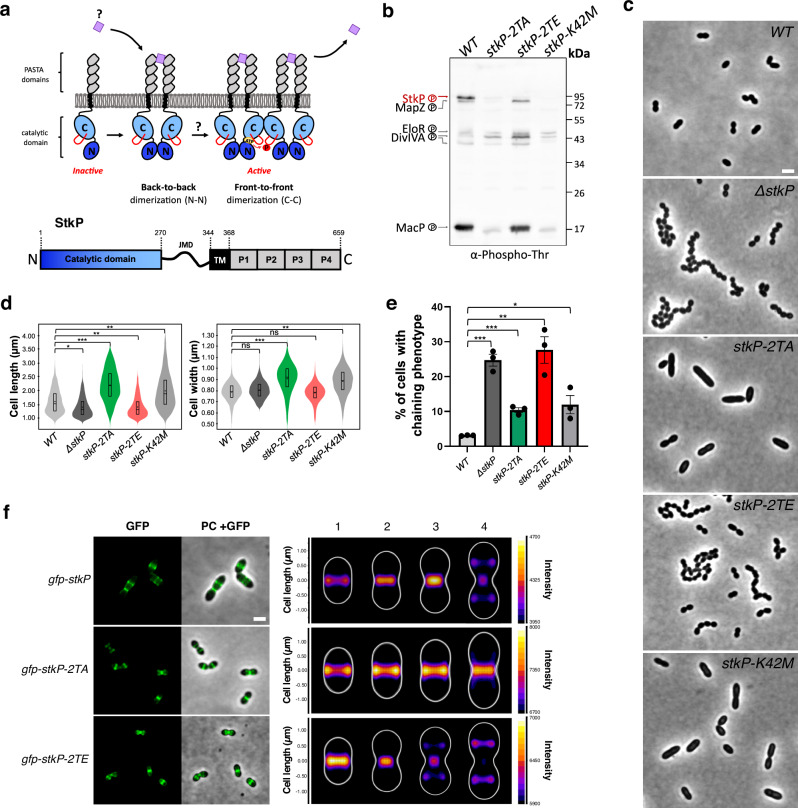
Phosphorylation of the activation loop is required for StkP kinase activity and cell division. **a** upper panel: Activation mechanism of bacterial PASTA-Hanks kinases. PASTA-Hanks-kinases form oligomers through back-to-back dimerization, enabling the activation of each monomer, triggered by ligand binding (purple square). Activation leads to phosphorylation (red “P”) of the activation loop (in red) of another monomer in a front-to-front conformation, independent of ligand binding. Lower panel: Schematic representation of StkP. The kinase domain is depicted with a gradient from dark to light blue. The juxtamembrane region is represented by a black linker, TM indicates the transmembrane domain, and the 4 PASTA domains are in gray. The amino acid numbering of each domain is indicated above. **b** Phosphorylation patterns probed with anti-phosphothreonine of *WT*, *stkP-2TA, stkP-2TE*, and *stkP-K42M* cells. The red arrow indicates StkP while black arrows show MapZ, DivIVA, EloR and MacP. **c** Representative phase contrast microscopy images of *WT*, *ΔstkP*, *stkP-2TA, stkP-2TE*, and *stkP-K42M* cells. Scale bar, 2 µm. (**d**) Violin plots showing the distribution of the cell length and cell width for *WT*, *ΔstkP*, *stkP-2TA, stkP-2TE*, and *stkP-K42M* strains. The box indicates the 25^th^ to the 75^th^ percentile. The mean and the median are respectively indicated with a dot and a line in the box. Statistical comparison was done using two-sided t-test (cell length: *P = 0.0114 (WT/*ΔstkP*), ***P = 0.0001 (WT/*stkP-2TA*), **P = 0.0042 (WT/*stkP-2TE*), **P = 0.0092 (WT/*stkP-K42M*); cell width: ns *P* > 0.05, ***P = 0.0008 (WT/*stkP-2TA*), **P = 0.0022 (WT/*stkP-K42M*)). 2000 cells were analyzed for each strain. **e** Bar chart representing the percentage of pneumococcal cell chaining (minimum five cells per chain). Bars represent the mean ( ± SEM) of three independent experiments. The WT was compared separately with the *ΔstkP*, *stkP-2TA, stkP-2TE*, or the *stkP-K42M* mutants with a two-sided unpaired t-test, ***P = 0.0002 (WT/*ΔstkP*), ***P = 0.0005 (WT/*stkP-2TA*), **P = 0.003 (WT/*stkP-2TE*), *P = 0.0278 (WT/*stkP-K42M*). **f** mGFP fluorescent signal and overlays between phase-contrast (PC) and mGFP images of m*gfp-StkP, mgfp-stkP-2TA* and m*gfp-stkP-2TE* cells. Scale bar, 2 µm. The heatmap represents the localization patterns during the cell cycle. 1000 cells were analyzed for each strain. Experiments in **b**, **d**–**f** were performed in triplicate.

The majority of bacterial Hanks-type kinases are membrane proteins that possess a variety of extracellular domains of different sizes that are poorly characterized^11^. Notably, some of these kinases exhibit an extracellular mosaic domain, found only in bacteria and named PASTA for “Penicillin-binding proteins And Serine/Threonine kinase Associated”^12^, that is proposed to bind peptidoglycan (PG) fragments or precursors^13,14^. PASTA-Hanks kinases have been shown to regulate different aspects of the bacterial cell cycle^15–18^. The current model proposes that PG binding could promote PASTA-Hanks kinases dimerization and then kinase activity. Consequently, and by analogy with the situation in eukaryotes, it is proposed that PASTA-Hanks kinases would behave as membrane receptors, signaling information about the cell wall status to endogenous phosphorylation targets, thereby regulating the bacterial cell cycle^7,16^ (Fig. 1a). However, other studies have challenged this model of ligand-induced kinase activity^14,19,20^ and the activation mode of PASTA-Hanks kinases remains an active and highly debated area of research.

Most of our knowledge regarding the structural organization of the cytoplasmic catalytic domain of bacterial Hanks kinases derives from enzymes from *Mycobacterium* species (PknA, PknB, PknE and PknG) and *Staphylococcus aureus* (PknB, also named PrkC or Stk1)^21–30^. Their kinase domains exhibit most of the conserved 11 amino acid motifs (I–XI)^10^ and the fold characteristic of eukaryotic Hanks kinases, with N- and C-lobes primarily composed of a β-sheet and an α-helical bundle, respectively (Supplementary Fig. 1). This configuration forms a central ATP- and substrate-binding region^7^. A key feature concerns the presence of an activation loop, which lies between motifs VII and VIII. This loop is characterized by its disordered state and contains two phosphothreonine residues that are required for kinase activity^27,28,30,31^. It is proposed that phosphorylation of the activation loop would modulate its conformation to allow ATP binding and subsequent substrate phosphorylation.

Among these structures, only two (PknB from *M. tuberculosis* and *S. aureus*) belong to PASTA-Hanks kinases^27,30,31^. Interestingly, both PknB homologs form dimers in their crystallographic structures, involving the backside of their N-lobes. This back-to-back dimer interface is proposed to allow allosteric activation of PknB by positioning the αC helix of the N-lobe in such a manner that the conserved glutamic acid of motif III can interact with the conserved lysine residue of subdomain II. Disrupting this interaction results in an alternative conformation of the αC helix and rearrangements of residues in the active site that render PknB inactive^29^. Alternatively, another study has shown that PknB can form front-to-front dimers through interaction between the C-lobes in which the activation loop of each monomer faces the active site of the other monomer^26^. This conformation would allow the trans-phosphorylation of the activation loop. The current model thus posits that PknB can form oligomers in which back-to-back dimerization enables the activation of each monomer, potentially upon ligand binding, so that one monomer can subsequently phosphorylate the activation loop of another monomer in the front-to-front conformation, independently of ligand binding (Fig. 1a). This model, however, lacks biological validation, and the in vivo activation mechanism of PASTA-Hanks kinases remains highly debated.

The PASTA-Hanks kinase StkP (Fig. 1a) is recognized as the central regulator of cell division and morphogenesis in the human bacterial pathogen *Streptococcus pneumoniae*. To our knowledge, StkP-mediated phosphorylation is one of the most thoroughly documented examples of the regulation of the bacterial cell cycle by phosphorylation^7,19,32,33^. However, no structural information is available for StkP, except for that of its fourth C-terminal PASTA domain, which regulates the activity of the cell wall hydrolase required for final separation of daughter cells^33^. Furthermore, it was demonstrated that StkP activation is independent of the ability of its four PASTA domains to bind PG^19^. Thus, the mechanisms by which StkP is activated and regulated remain to be elucidated.

In this study, we resolve the structures of different forms of the cytoplasmic catalytic domain of StkP, harboring amino acid substitutions on the two threonine residues of the activation loop. Based on these structures, we perform in silico, in vitro and in vivo analyses that provide insights into the activation mechanism of StkP. These results demonstrate that an alternative dimerization interface, along with the flexibility of the activation loop, governed by its phosphorylation, is critical for the activation of StkP. Overall, we propose a model in which StkP initially forms at the division septum an inactive, concentration-dependent dimer, which is further reorganized to allow the phosphorylation of the StkP activation loop and subsequent endogenous substrate phosphorylation.

## Results

### Phosphorylation of the activation loop is required for StkP kinase activity and cell division

StkP comprises an extracellular domain with four PASTA domains and a cytoplasmic catalytic domain, which spans from residue 1 to residue 344, composed of the catalytic domain and the flexible juxtamembrane domain (JMD) (Fig. 1a)^34^. As in other Hanks-type kinases, the activation loop (residues 157 to 179) is positioned between the conserved DFG (Hanks motif VII) and SPE (Hanks motif VIII) signatures. Its sequence shows the presence of two threonine residues, T167 and T169, that are conserved in the other PASTA-Hanks kinases (Supplementary Fig. 1a)^7,35^. To investigate the role of these residues in StkP kinase activity, we generated two mutant strains, *stkP-2TA* and *stkP-2TE*. The *stkP-2TA* mutant carries T167A/T169A substitutions, preventing phosphorylation at these sites, whereas the *stkP-2TE* mutant harbors T167E/T169E substitutions, mimicking permanent phosphorylation. Throughout this study, *stkP* mutations and/or fluorescent fusions were constructed at each native chromosomal locus, expressed under the control of the native promoter and represented the only source of protein. The two StkP variants demonstrated stability and production levels comparable to those of wild-type StkP (Supplementary Fig. 2a). In WT cells, the standard phosphorylation pattern was detected with signals corresponding to StkP autophosphorylation and the phosphorylation of the four cell division proteins, MapZ, EloR, DivIVA and MacP^15,36–38^ (Fig. 1b). The same observation was made for the *stkP-2TE* variant, which retained substrate phosphorylation. Only the signal of StkP itself was strongly decreased, reflecting the absence of phosphothreonine residues in the activation loop and a phosphorylation limited to the JMD^34^ (Fig. 1b). By contrast, the analysis of *stkP-2TA* strain showed that StkP-mediated phosphorylation was severely altered (Fig. 1b). This observation is reminiscent of the defective phosphorylation pattern previously reported for the catalytically inactive *stkP-K42M* mutant^15^ (Fig. 1b).

Growth and cell morphology analyses further revealed that the *stkP-2TA* strain exhibited growth and cell shape defects similar to those observed for the *stkP-K42M* mutant^15^ (Fig. 1cd and Supplementary Fig. 2b). Indeed, morphometric measurements revealed that 25% of the WT cell population were below and 25% above the quartiles Q1 (1.24 µm) and Q3 (1.88 µm), respectively. In stark contrast, 68.6% of the population of *stkP-2TA* cells were elongated and above the Q3 quartile (Fig. 1d). Additionally, we observed a slight tendency for cells to form chains (Fig. 1e). Regarding *stkP-2TE* cells, cell growth was only slightly affected (Supplementary Fig. 2b). However, cells were less elongated than WT cells with 42.75 % of the cell population below the Q1 quartile of WT cells (Fig. 1cd). Moreover, cell chaining increased as it did for ∆*stkP* cells, with more than 25% of cells in chains (Fig. 1e).

We also examined the potential impact of 2TA and 2TE substitutions on StkP localization. For that purpose, we constructed two strains producing N-terminal mGFP fusions of either StkP-2TA (*mGFP-stkP-2TA*) or StkP-2TE (*mGFP-stkP-2TE*). As previously reported for mGFP-StkP^15^, the two mutated fusion forms were stable (Supplementary Fig. 2c). They generated the same growth defects as non-mGFP-fused forms (Supplementary Fig. 2b, d). As shown in Fig. 1f, mGFP-StkP localized at the division septum as previously reported^15^. However, strikingly, the heatmap analysis revealed that the dynamics of StkP were affected by phosphomimetic and phosphoablative mutations. The mGFP-StkP-2TE fluorescence signal localized earlier at the cell equator, which corresponds to the future division site of daughter cells, and was less intense at the division site than in WT cells (cell division stages 3 and 4). On the contrary, mGFP-StkP-2TA persisted longer at the division site, and its localization at the cell equator was delayed (Fig. 1f). These observations indicate that the phosphorylation of T167 and T169 in the activation loop (i) is required for StkP activity and substrate phosphorylation and (ii) influences the dynamics of StkP localization. They also suggest that cycling between both forms is required for proper pneumococcal growth and cell division.

### Structure of the StkP cytoplasmic domain

**Fig. 2:**
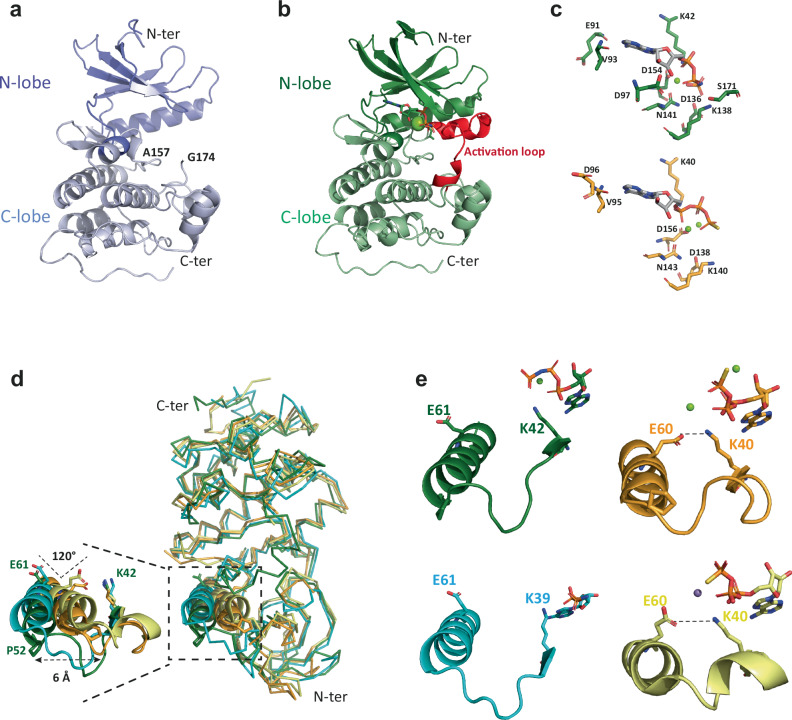
Structural characterization of the StkP cytoplasmic domain. **a** Cartoon representation of StkP^344^−2TA (molecule B). The activation loop and the C-terminal part are not visible. **b** Cartoon representation of StkP^287^−2TA (molecule B) in complex with AMP-PNP and Mg²⁺. The activation loop is visible and depicted in red. AMP-PNP (in sticks) and Mg²⁺ (green sphere) are bound in a pocket between the N-lobe (dark green) and the C-lobe (light green). **c** Binding site of AMP-PNP. The bound nucleotides (in sticks) and Mg²⁺ (green spheres) with key active site residues in StkP^287^−2TA (green, upper panel) and PknB-ATP-γ-S (orange, PDB entry 1MRU, lower panel) highlight the conserved nucleotide binding and catalytic mechanism. **d** Orientation of αC-helix. Superposition of the StkP^287^−2TA structure from *S. pneumoniae* (green) with the kinase domain structures of PknB from *M. tuberculosis* in its active (orange, PDB entry 1MRU) and inactive (yellow, PDB entry 3ORM) forms, and the inactive kinase domain of PknB from *S. aureus* (cyan, PDB entry 4EQM). While the overall superposition aligns well, the orientation of helix αC shows significant differences. Nucleotides and metal ions are omitted for clarity (**e**) Conformational changes (αC and the K42-E61 salt bridge) in the active site of StkP^287^−2TA (green) are observed compared to the active (orange, PDB entry 1MRU) and inactive forms of PknB from *M. tuberculosis* (yellow, PDB entry 3ORM) and *S. aureus* (cyan, PDB entry 4EQM).

We then sought to determine the structure of the full cytoplasmic domain of StkP (residues 1– 344) to gain insight into the potential conformational changes generated by the 2TA and 2TE substitutions. The phosphorylation variants StkP-2TA and StkP-2TE were thus purified and subjected to crystallization (Supplementary Fig. 3a). Well-diffracting crystals were only obtained for the StkP-2TA form. They diffracted to 2.8 Å resolution in space group P2₁ (Supplementary Table 1). The structure (referred to as StkP^344^−2TA) contained two molecules per asymmetric unit: molecule A was resolved between residues 4-155 and 174–271, while molecule B was built between residues 4–46, 50-156 and 176–274 (Fig. 2a and Supplementary Fig. 3b). No electron density was observed for most of the activation loop, suggesting structural flexibility in this region. The same is true for the C-terminal part of the construct. The latter corresponds mainly to the JMD linking the catalytic domain to the transmembrane helix^34^. We thus redesigned the construct of the StkP-2TA cytoplasmic domain to residues 1–287 (referred to as StkP^287^−2TA), excluding the flexible JMD segment, to improve crystal packing and resolution. AMP-PNP and Mg²⁺ or Mn²⁺ were added to the purified samples to stabilize the kinase domain.

After purification and crystallization (Supplementary Fig. 3a), the truncated StkP^287^−2TA variant yielded better-diffracting crystals in a P2₁ space group and the structure was determined in complex with AMP-PNP and either Mg²⁺ (at 1.8 Å resolution) or Mn²⁺ (at 1.9 Å resolution) (Supplementary Table 1). In common with the StkP^344^−2TA structure, these two crystal structures contained two molecules per asymmetric unit (Supplementary Fig. 3c). Both A and B molecules exhibited a similar arrangement, with a Cα r.m.s.d. of 0.7 Å between them. However, and importantly, molecule B was fully resolved, including the complete backbone of the activation loop, whereas three segments (residues 3–43, 55-158 and 176–285), with most the activation loop missing (residues 159–175), were built in molecule A (Fig. 2b and Supplementary Fig. 3c). Interestingly, the electron density was also missing between residues 44 to 54, which corresponds to the N-terminus region of helix αC. Since the crystallographic packing was identical to that of StkP^344^−2TA, we inferred that the stabilization of the activation loop resulted from the addition of AMP-PNP and cations, rather than crystal packing or contacts. Only other minor conformational differences between B molecules of StkP^287^−2TA and StkP^344^−2TA were observed, primarily in helix αG (Hanks motif X), G-loop (Hanks motif I) and the relative orientation of the N-lobe to the C-lobe (Supplementary Fig. 3d).

### Characterization of the StkP active site

**Fig. 3:**
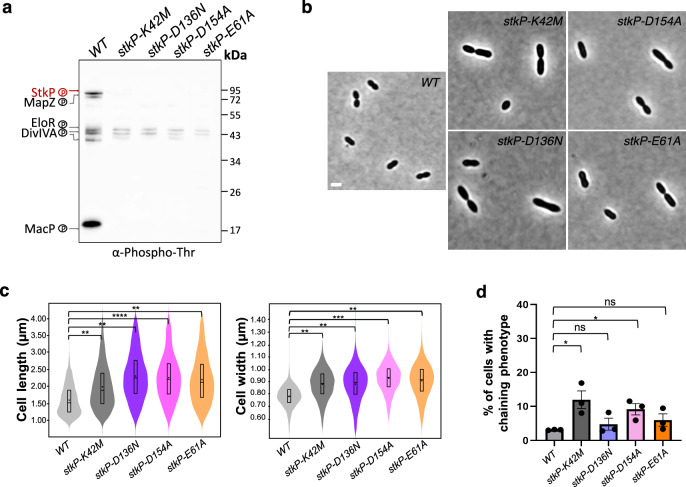
Functional characterization of StkP mutants. **a** Phosphorylation patterns. Western immunoblots of whole-cell lysates from *WT*, *stkP-K42M*, *stkP-D136N, stkP-D154A*, and *stkP-E61A* cells. Cells were probed with anti-phosphothreonine antibodies. StkP is shown in red and StkP substrates (MapZ, DivIVA, EloR and MacP) are indicated in black. Images are representative of experiments made in triplicates. **b** Representative phase contrast microscopy images of *WT*, *stkP-K42M*, *stkP-D136N, stkP-D154A*, and *stkP-E61A* cells. Scale bar, 2 µm. (c) Violin plot showing the distribution of the cell length (left panel) and cell width (right panel) for *WT*, *stkP-K42M*, *stkP-D136N, stkP-D154A*, and *stkP-E61A* strains as determined using MicrobeJ^55^. The distribution of the cell length and width are shown in light gray for the *WT* strain, in medium gray for the *stkP-K42M* strain, in purple for the *stkP-D136N* strain, in magenta for the *stkP-D154A* strain and in orange for the *stkP-E61A* strain. The box indicates the 25^th^ to the 75^th^ percentile. The mean and the median are respectively indicated with a dot and a line in the box. Statistical comparison was done using a two-sided t-test (cell length: **P = 0.0092 (WT/*stkP-K42M*), **P = 0.001 (WT/*stkP-D136N*), ****P < 0.0001 (WT/*stkP-D154A*), **P = 0.0067 (WT/*stkP-E61A*); cell width: **P = 0.0022 (WT/*stkP-K42M*), **P = 0.0041 (WT/*stkP-D136N*), ***P < 0.0002 (WT/*stkP-D154A*), **P = 0.0024 (WT/*stkP-E61A*). 2000 cells were analyzed for each strain and each replicate. Experiments were conducted in triplicates. **d** Bar chart representing the percentage of pneumococcal cells harboring a chaining phenotype (minimum five cells per chain). Bars represent the mean ( ± SEM) of three independent experiments. The WT was compared separately with the *stkP-K42M*, *stkP-D136N, stkP-D154A*, or *stkP-E61A* mutants a two-sided unpaired t-test, ns *P* > 0.05, *P = 0.0277 (WT/*stkP-K42M*), *P = 0.0231 (WT/*stkP-D154A*).

The StkP^287^−2TA domain in complex with AMP-PNP adopts the conserved two-lobed architecture previously described in PASTA-Hanks kinase homologs PknB from *Mycobacterium tuberculosis* or *Staphylococcus aureus*^27,28,30^ (Fig. 2b). The AMP-PNP was clearly visible and binds within the inter-lobe cleft, forming critical interactions similar to those described in PknB structures (Fig. 2c). Notably, the structure underscores the critical role of D154 from the Hanks-motif VII in Mg²⁺ coordination and of D136 from the Hanks-motif VI in the interaction with the nucleotide analog (Fig. 2c). To confirm these observations in vivo, we constructed the *stkP-D154A* and *stkP-D136N* mutant strains and analyzed their phosphorylation patterns, growth, cell shape and chaining (Fig. 3 and Supplementary Fig. 2e). As expected, these two mutations abolished StkP kinase activity, leading to altered growth and cell morphology defects comparable to those observed in the *stkP-2TA* and *stkP-K42M* mutants.

Importantly, in the StkP^287^−2TA structure, the αC helix, which carries the Hanks motif III, adopts a conformation that differs from that in active PknB^27^. Its position is translated by around 6 Å (measured at the level of P52) and tilted by around 120° away from the N-lobe (Fig. 2d). In this αC orientation, referred to as the αC-out conformation, the conserved E61 of Hanks motif III is turned away from the active site and cannot form the salt bridge with K42 of Hanks motif II, which is a hallmark of active protein kinases^39,40^ (Fig. 2e). This was also consistent with the deficient phosphorylation patterns displayed by the *stkP-K42M* mutant (Fig. 1b). Interestingly, the formation of this canonical K42–E61 salt bridge is also prevented in inactive PknB mutated on the back-to-back interface^29^ (Fig. 2e). To functionally assess the importance of this salt bridge in StkP activation, we constructed the *stkP-E61A* strain and showed that it exhibited a complete loss of kinase function and displayed morphological defects identical to those of the other inactive mutants (Fig. 3 and Supplementary Fig. 2e).

### Role of the activation loop in StkP activity

**Fig. 4:**
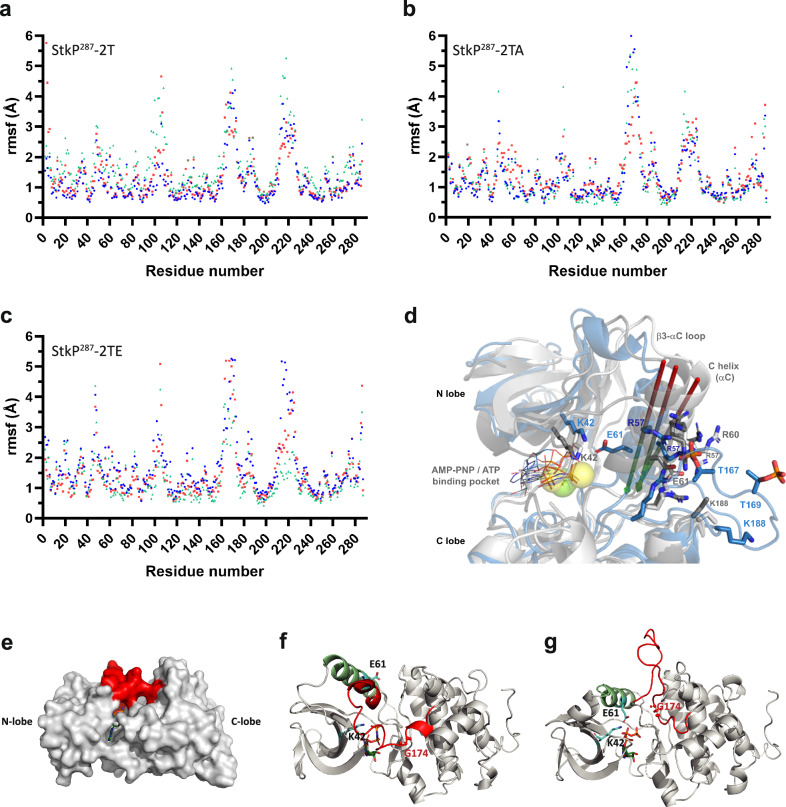
Characterization of the activation loop flexibility. Root-mean-square fluctuations per residue (RMSF) were calculated in triplicates (three colors) using the cpptraj module of AmberTools for (**a**) StkP^287^−2T, (**b**) StkP^287^−2TA and (**c**) StkP^287^−2TE. **d** Superposition of the active *wt*StkP-2pT model (blue cartoon – Supplementary Fig. 6) onto chains A (dark gray) and B (light gray) of the X-ray crystal structure StkP^287^−2TA. RMS fitting was performed using only C-lobe Cα atoms, yielding an RMSD of 0.775 Å (136 atoms) between the theoretical and experimental structures. This overlay reveals the activation switch driven by coordinated motion of the activation loop and the αC helix. In the inactive state, the outward-shifted conformation of αC disrupts the crucial salt bridge between K42 (in β3 strand) and E61 (in αC). The αC deviation angle between inactive (gray) active (blue) conformations is defined using a vector along αC (red-to-green gradient), calculated using all main-chain atoms (N, O, C) with a 3.5 Å hydrogen bond cutoff. The inactive αC in chain A is shifted away by 16.9° from the active state αC conformation in the 2pT simulated structure, which displays a stable K42-E61 salt bridge. The also inactive αC in chain B adopts an intermediate state (7.5°), suggesting a transition between inactive and active forms. AMP-PNP and ATP are shown as lines; the coordinated Mg^2+^ ions are shown as spheres (green in crystal structure, yellow in simulations). **e** Surface representation of StkP^287^−2TA showing the activation loop (red) partially blocking the AMP-PNP binding site. **f** Conformational state of the activation loop in the crystal structure of StkP^287^−2TA. The αC helix (green) adopts an “αC-out” conformation, and the K42–E61 salt bridge (cyan sticks) is disrupted. **g** Conformational state of the activation loop in the AlphaFold 3 model of StkP^287^-WT bound to ATP and Mg²⁺ (Supplementary Fig. 6), showing an “αC-in” conformation with restoration of the K42–E61 interaction. G174 of the conserved Hanks motif VII (DFG), acting as a pivot for activation loop rearrangement, is indicated.

As stated above, the complete backbone of the activation loop was clearly visible in the electron density map of the StkP^287^−2TA structure obtained in complex with AMP-PNP bound to either Mg²⁺ or Mn²⁺ (Fig. 2b and Supplementary Fig. 3c). Although fully resolved activation loops have previously been reported for some bacterial Hanks kinases, for example in the *Mycobacterium tuberculosis* PknG structures^41,42^, this represents the first structure of a bacterial PASTA-Hanks kinase in which the activation loop is fully resolved. The loop is positioned at the mouth of the catalytic cleft between the two lobes and reveals an interaction between S171 and the β-phosphate of the AMP-PNP molecule (Fig. 2c). To gain insight into the stability of this loop, we performed molecular dynamics (MD) simulations of this phosphoablative (2TA) variant based on the crystallographic structure (the dimer or monomeric molecule B) upon replacement of AMP-PNP with Mg^2+^-bound ATP (Supplementary Video 1). For completeness, the StkP^287^-WT, its phosphorylated counterpart (StkP^287^−2pT) and the phosphomimetic variant (StkP^287^− 2TE) were also simulated under identical conditions (Supplementary Videos 2–5). The activation loop adopted a manifold of conformations, depending on the initial configuration (three non-identical replicas) and threonine modifications, which reflect its intrinsic structural variability (Fig. 4a–d). In StkP^287^−2TA and StkP^287^-WT, the activation loop remained in a conformation that obstructs the active site and the K42-E61 salt bridge is not formed (Fig. 4e, f). In sharp contrast, in StkP^287^−2TE and StkP^287^−2pT, the loop was highly flexible, thereby opening the active site to endogenous substrates (Fig. 4d, g). We noted that the glycine residue, G174, from the critical Hanks motif VII (DFG), facilitated the movement of the activation loop (Supplementary Video 2, 3 and 4). To investigate this further, we generated a *stkP-G174P* mutant strain, as proline is known to restrict flexibility. Similar to the catalytic mutant *stkP-K42M*, this strain exhibited severely impaired cell growth (Supplementary Fig. 4a) and a loss of kinase function, as attested by the absence of StkP autophosphorylation and substrate phosphorylation (Supplementary Fig. 4b). Nevertheless, the effects observed in Supplementary Fig. 4 may not arise solely from reduced activation-loop flexibility. In addition to limiting backbone mobility, substitution of Gly174 with proline eliminates a free backbone amide NH group that could participate in peptide-mediated interactions. Accordingly, Gly174 may contribute not only to loop flexibility but also to backbone-mediated contacts involved in substrate recognition or binding, which could be perturbed by the proline substitution. Thus, additional structural or functional consequences may also contribute to the observed growth phenotype.

To support the MD simulations experimentally, we also purified and solved the structure of the active StkP^287^−2TE variant in complex with AMP-PNP and Mn²⁺ at 1.6 Å resolution (Supplementary Fig. 3a and 5a). This protein crystallized in the same space group as StkP^287^−2TA, with similar crystal packing and two molecules per asymmetric unit arranged identically to those in StkP^287−^2TA (Supplementary Table 1 and Supplementary Fig. 5a). A key difference, however, was found in the activation loop, which could not be resolved for any of the monomers due to lack of electron density in the corresponding region, despite the higher resolution and the overall similarity (Supplementary Fig. 5a). This structure thus supports our MD simulation results, indicating that the activation loop is highly dynamic when glutamate residues substitute for T167 and T169, thus mimicking the functional state of the phosphorylated threonines. Nonetheless, it is worth noting that the αC helix in the N-terminal lobe of StkP^287^−2TE is positioned as in StkP^287^-2TA, preventing the formation of the K42–E61 salt bridge (Supplementary Fig. 5a). This was unexpected, given that the *stkP−2TE* mutant is active in vivo (Fig. 1b). To shed further light on this issue, we used AlphaFold 3 (AF3) to build and simulate models of StkP^287^−2TE in which Mg^2+^-bound ATP replaces the nucleotide analog AMP-PNP used for crystallization (Supplementary Fig. 5b and Supplementary Fig. 6). The AF3 structures were very similar to the X-ray crystal structure (rmsd = 0.927 Å over 255 Ca atoms), the degree of confidence being notably lower for the activation loop relative to the rest of the protein (Supplementary Fig. 5b and Supplementary Fig. 6). However, a critical difference concerned the active site region. The αC helix adopts a “αC-in” conformation similar to that observed in the active PknB structure and the K42–E61 salt bridge is formed (Fig. 4fg). The same result was obtained when a homology model of StkP^287^−2pT was built (and later simulated) using as template the X-ray crystal structure of the activated catalytic subunit of cAMP-dependent protein kinase (PKA; PDB code: 4HPU) (Supplementary Fig. 6). These observations suggest that, besides the phosphorylation state of the activation loop, the nature of the bound nucleotide is also important for the formation of this key electrostatic interaction.

### An unusual asymmetric interface drives the dimerization of the StkP kinase domain

**Fig. 5:**
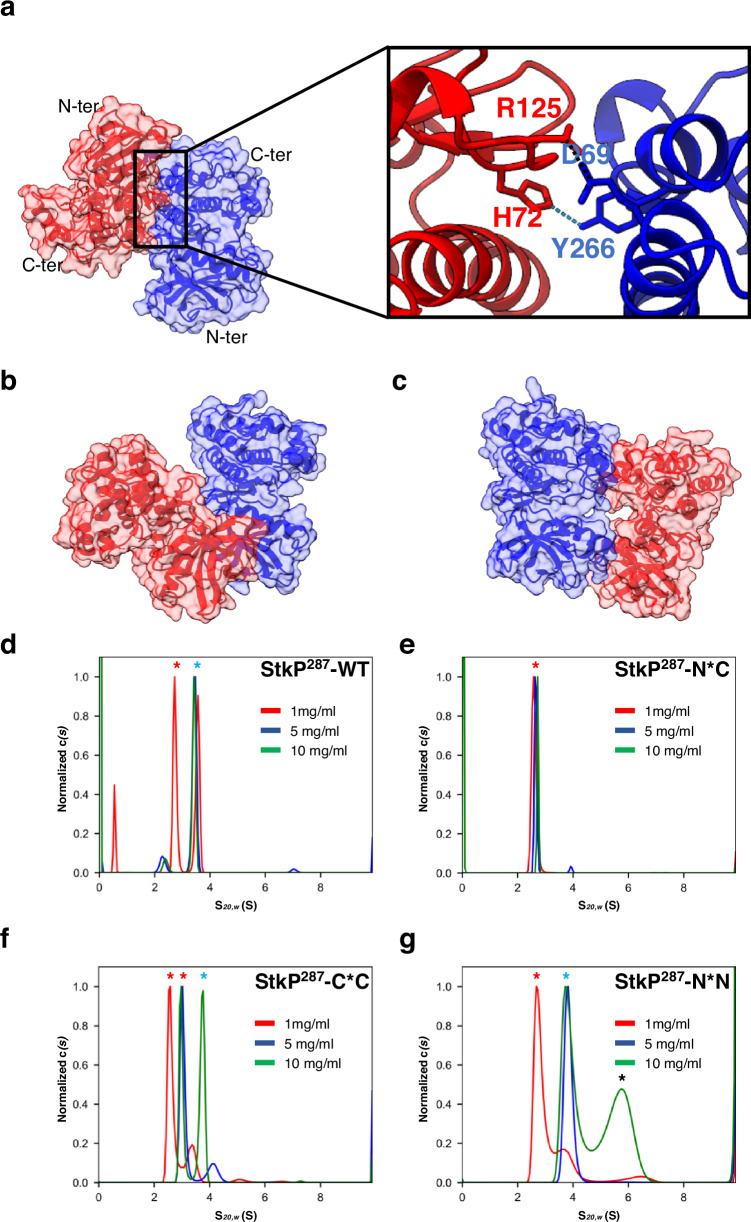
A unusual asymmetric interface drives the dimerization of the StkP kinase domain. **a** Cartoon with surface representation of the StkP kinase domain dimer formed through the N-lobe–C-lobe interface. Molecule A is shown in red, and molecule B in blue. The interactions between D69 and R125 and between H72 and Y266 are depicted as dotted lines. **b** Cartoon and surface representation of the *M. tuberculosis* PknB kinase domain dimer in a front-to-front orientation (mediated by C-lobe–C-lobe interactions). **c** Cartoon and surface representation of the *M. tuberculosis* PknB kinase domain dimer in a back-to-back orientation (mediated by N-lobe–N-lobe interactions). **d**–**g** Normalized c(s20,w) distributions obtained from interference data for StkP287-WT (**d**), StkP287- N*C (**e**), StkP287-C*C (**f**) and StkP287-N*N (**g**) at concentrations of 1 mg/mL (red), 5 mg/mL (blue), and 10 mg/mL (green). The stars indicate the positions of the peaks corresponding to a globular monomer (red stars), globular dimers (blue stars) and trimers (black star).

All of the structures, namely StkP^344^−2TA, StkP^287^−2TA, and StkP^287^-2TE, crystallized in the same space group, forming an unusual asymmetric dimer in the asymmetric unit. Indeed, the interface (named NC interface) involved residues from the N-lobe of one monomer and residues from the C-lobe of the other monomer (Fig. 5a). It differed from that of PknB that also crystallized with two molecules in the asymmetric unit, but in different space groups. Indeed, dimers of PknB form either through back-to-back or front-to-front interfaces, with dimerization occurring via N-lobe-N-lobe (NN) and C-lobe-C-lobe (CC) interactions, respectively^26,29^ (Fig. 5bc). The NC interface represents a surface of ~784 Å², in the same range as the calculated surface areas for CC (790 Å², PDB code: 1MRU) and NN dimers (883 Å², PDB code: 3F69).

Polar and charged residues primarily mediate intermolecular contacts, including an ionic interaction between D69 and R125 and a hydrogen bond between H72 and Y266 (Fig. 5a). These interacting residues are located immediately after the αC helix (D69, H72), just before (R125) the catalytic loop (Hanks domain VI), or within helix αI (Y266). Although most of these residues are not conserved in PknB homologs, they are conserved in other *Streptococcus* species, where similar polar or charged residues are found at equivalent positions (Supplementary Fig. 7).

To characterize the oligomeric state of the StkP catalytic domain in solution and determine whether the observed NC interface is not solely due to crystal packing, we performed analytical ultracentrifugation (AUC) experiments. We first examined the behavior of purified StkP^287^-WT (Supplementary Fig. 3a) at different concentrations ranging from 0.2 to 10 mg/mL (Fig. 5d and Supplementary Fig. 8a–c). The AUC c(s) distributions revealed two main peaks. The first peak corresponds to a globular monomer, with f/fmin = 1.12 and the second one is consistent with a globular dimer, with f/fmin = 1.28 (Fig. 5d). Non-Interacting Species (NIS) confirmed these assignments estimating a molecular weight of 30 + /− 2.7 kDa for the first peak (close to the theoretical MW of a StkP^287^-WT monomer, 32.4 kDa) and 70.6 + /− 2.7 kDa for the second peak (theoretical dimer MW of 64.8 kDa). The peak corresponding to the WT monomer decreased as the concentration increases: it accounts for 50% at 1 mg/ml, 14% at 5 mg/ml, and 10% at 10 mg/ml (Fig. 5d). On the other hand, the proportion of the peak attributed to the dimer, increased in a concentration-dependent manner. It represents 50% at 1 mg/ml, 86% at 5 mg/ml, and 90% at 10 mg/ml (Fig. 5d). These variations in the proportion of peaks, favoring the larger species with increasing concentration, suggest the presence of an association-dissociation equilibrium. The stability of the s values and the NIS analysis further suggest a slow monomer-dimer equilibrium, with *Kd* = 37 µM (Supplementary Fig. 8c) ^43^. The SAXS experiments performed at different concentrations of StkP^287^-WT supported these observations (Supplementary Table 2). Indeed, we measured that the radius of gyration (Rg), the maximum diameter of particles (Dmax) and the volume values of the particles in solution progressively increased with concentration. Together, these data confirmed that StkP forms dimers in solution. However, the monomer-dimer equilibrium, demonstrated by both AUC and SAXS analyses, indicates that dimerization is not constitutive but rather governed by concentration.

We then purified StkP^287^ carrying two mutations (D69A and Y266A) designed to disrupt the identified NC interface (StkP-N*C) (Supplementary Fig. 3a), and performed the same AUC experiments as for the WT protein (Fig. 5e and Supplementary Fig. 8c). Strikingly, at all three tested concentrations, the sedimentation profile of StkP-N*C revealed a single peak, which corresponds to a globular monomer (f/fmin = 1.37), as confirmed by NIS analysis, which gave a MW of 30 + /− 2.5 kDa. These results demonstrated that the D69A and Y266A mutations, which target the interface revealed in our crystal structures, disrupt the dimeric assembly, indicating that this NC interface is not a consequence of crystallization but may rather be physiologically relevant for StkP function.

Interestingly, the amino acids involved in back-to-back (NN interface) and front-to-front (CC interface) dimerization of PknB are conserved in StkP (Supplementary Fig. 1a). We thus purified two StkP^287^ mutants carrying either D76A and L33D (StkP-N*N) or V22D and A225E (StkP-C*C) substitutions (Supplementary Fig. 3a), previously described to disrupt the respective interfaces and performed AUC experiments to assess their oligomeric state (Fig. 5fg). Surprisingly, the sedimentation profiles of both mutants at 1 and 5 mg/mL revealed two peaks, corresponding to a globular monomer and a dimer, similar to the WT protein. At 10 mg/mL, an additional peak consistent with a higher-order oligomeric species was even observed. NIS analysis confirmed the molecular weights of the observed peaks, supporting the presence of monomeric, dimeric, and oligomeric forms of StkP^287^-N*N and StkP^287^-C*C (Fig. 5fg and Supplementary Fig. 8d). These observations demonstrate that, in solution, the dimer of the StkP catalytic domain relies on interaction between the N-lobe and the C-lobe, and differs from the model proposed for PknB.

### Biological relevance of the NN, CC and NC interface

**Fig. 6:**
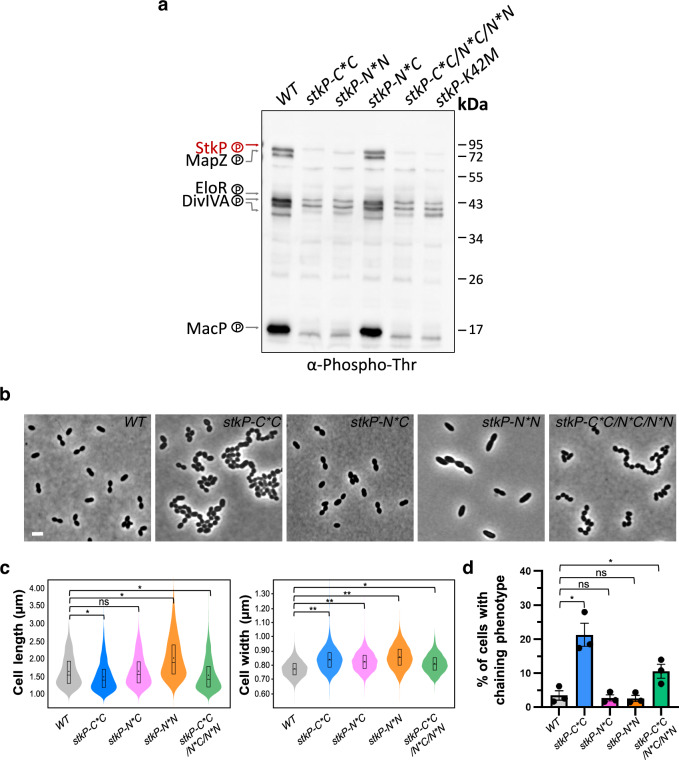
Functional characterization of the NC dimerization interface of the StkP kinase domain. **a** Phosphorylation patterns. Western immunoblots of whole-cell lysates from *WT*, stkP-C*C, stkP-N*N, stkP-N*C and stkP-N*C/C*C/N*N cells. Cells were probed with anti-phosphothreonine antibodies. StkP is shown in red and its substrates (MapZ, DivIVA, EloR and MacP) in black. Images are representative of experiments made in triplicates. **b** Representative phase contrast microscopy images of *WT*, stkP-N*C, stkP-C*C, stkP-N*N, and stkP-N*C/C*C/N*N cells. Scale bar, 2 µm. **c** Violin plot showing the distribution of the cell length (left panel) and cell width (right panel) for *WT*, stkP-N*C, stkP-C*C, stkP-N*N, and stkP-N*C/C*C/N*N strains as determined using MicrobeJ^55^. The distribution of the cell length and width are shown in gray for the *WT* strain, in blue for the stkP-C*C strain, in magenta for the stkP-N*C strain, in orange for the stkP-N*N strain and in green for the stkP-N*C/C*C/N*N strain. The box indicates the 25^th^ to the 75^th^ percentile. The mean and the median are respectively indicated with a dot and a line in the box. Statistical comparison was done using a two-sided t-test (cell length: ns *P* > 0.05, *P = 0.042 (WT/stkP-C*C), *P = 0.0298 (WT/stkP-N*N), *P = 0.046 (WT/stkP- N*C/C*C/N*N); cell width: **P = 0.0015 (WT/stkP- C*C), **P = 0.001 (WT/stkP- N*C), **P = 0.0052 (WT/stkP- N*N), *P = 0.0258 (WT/stkP- N*C/C*C/N*N). 2000 cells were analyzed for each strain and each replicate. Experiments were conducted in 3 replicates. **d** Bar chart representing the percentage of pneumococcal cells harboring a chaining phenotype (minimum five cells per chain). Bars represent the mean ( ± SEM) of three independent experiments. The WT was compared separately with the stkP-N*C, stkP-C*C, stkP-N*N, or stkP-N*C/C*C/N*N mutants with a two-sided unpaired t-test, ns *P* > 0.05, *P = 0.025 (WT/stkP- C*C), *P = 0.0435 (WT/stkP- N*C/C*C/N*N).

To determine the biological relevance of the three potential interfaces, we constructed the three stkP-N*C, stkP-N*N and stkP-C*C mutated pneumococcal strains to analyze their phosphorylation pattern, growth and cell morphology. Recapitulating observations for PknB^26,29^, the phosphorylation patterns for both stkP-N*N and stkP-C*C strains were strongly altered (Fig. 6a), reflecting that back-to-back and front-to-front interactions are required in vivo for StkP activity. Consistent with this, stkP-N*N cells were elongated and similar to *stkP-K42M* cells (Fig. 6b, c). On the other hand, stkP-C*C cells were shorter, rounded and chained, similar to *StkP-2TE* cells (Fig. 1e and Fig. 6d). Both mutants were also impaired in growth (Supplementary Fig. 9a). Surprisingly, the stkP-N*C strain displayed a growth and a phosphorylation pattern similar to that of WT cells (Fig. 6a and Supplementary Fig. 9b). In addition, cells were similar to WT cells even if slightly wider (Fig. 6b, c). To rule out that mutation of the different interfaces affected StkP stability and/or localization, we constructed strains producing the corresponding mGFP fusions. mGFP-StkP-N*C, mGFP-StkP-N*N and mGFP-StkP-C*C were stable and localized properly at the division septum (Supplementary Fig. 9b, c). These observations show that the NC dimerization interface of StkP in solution is not required for StkP-mediated phosphorylation. On the contrary, they suggest that the NN and CC StkP interfaces, which are not involved in dimerization in solution (Fig. 5f, g), are critical for StkP kinase activity. Also importantly, the absence of an elongated phenotype for stkP-C*C cells suggests that mutating the C-lobe-C-lobe interface suppresses the need of StkP kinase activity for cell division. To check this, we generated the stkP-C*C/N*C/N*N mutant, in which all three interfaces were disrupted. As expected, this StkP mutant was fully inactive (Fig. 6a), and cells exhibited growth defects, altered morphology, and chaining phenotypes nearly identical to those of stkP-C*C (Supplementary Fig. 9a and Fig. 6b–d). This similarity underscores the dominant role of the CC interface in StkP function, as its disruption alone recapitulates the triple mutant phenotype. Collectively, our data highlight a complex interplay between dimerization, kinase activation, and pneumococcal cell division.

## Discussion

The Ser/Thr kinase StkP plays a pivotal role in controlling cell division and morphogenesis in *Streptococcus pneumoniae*^15^. Although previous studies have described its role in phosphorylating various cell division proteins^15,36–38^, how StkP kinase activity is triggered still remains unclear. In this study, we present the structural characterization of the StkP’s cytoplasmic domain in its phosphoablative and phosphomimetic forms. Importantly, these structures provide the structural insights into the organization of the activation loop and reveal an unusual dimerization mode of the catalytic domain. In vitro and in vivo experiments further demonstrate the biological relevance of these structural observations, shedding a light on the activation mechanism of StkP.

Our data reveal that the phosphorylation of the two conserved threonines, T167 and T169, acts as a molecular switch that regulates the conformational state of the activation loop and the activity of StkP (Fig. 1). Indeed, the phosphoablative mutant StkP-2TA behaves like the catalytically dead K42M mutant, exhibiting drastically reduced substrate phosphorylation. This finding is consistent with the observations reported for other bacterial Hanks-kinases, such as *Enterococcus faecalis* IreK and *Mycobacterium tuberculosis* PknB, where the mutation of the threonine residues in the activation loop severely impairs kinase activity^31,44,45^. In contrast, the phosphomimetic StkP-2TE mutant demonstrates full activity. Consequently, and as expected, the phosphoablative *stkP-2TA* mutant cells are elongated cells and display division defects, similar to the *stkP-K42M* mutant cells (Fig. 1). However, the cell shape of the phosphomimetic *stkP-2TE* mutant is also affected, albeit not as drastically, producing small and chained cells (Fig. 1c–e). This shows that the absence of StkP activity is significantly detrimental, impeding cell division. Conversely, permanent phosphorylation of StkP enables cell division, although it alters cell elongation and final separation. Furthermore, we observed that StkP-2TE localizes early to the future division sites at cell equators, whereas StkP-2TA persists at the septum with delayed re-localization (Fig. 1f). This finding is consistent with the recent discovery that the phosphorylation of the StkP’s JMD, which is dependent on prior phosphorylation of the activation loop, also impacts the dynamics of StkP localization^34^. Imbalance of StkP activity has thus a significant impact on multiple division stages of the cell cycle progression with both phosphorylated and unphosphorylated states of StkP being required. It highlights the complexity of the underlying regulatory mechanisms and the significance of stringent regulation of StkP kinase activity through activation loop phosphorylation to control StkP intracellular dynamics and to ensure cell division. In this context, it can be hypothesized that the StkP cognate phosphatase PhpP^46^, which is required for proper cell division, might be critical in this regulation process.

Structurally, the activation loop adopts a variety of partially unfolded states that obstructs the active site in the unphosphorylated StkP^287^−2TA form (Figs. 2 and 4). This positioning is stabilized by a direct interaction between S171 and the β-phosphate of AMP-PNP, suggesting a mechanism by which the activation loop can effectively block substrate access in the inactive state. In contrast, the phosphomimetic StkP^287^−2TE loop is highly flexible and unresolved in the crystallographic structure, a dynamic behavior that is supported by MD simulation results showing increased loop mobility and active site accessibility similar to that of StkP^287^−2pT (Fig. 4 and Supplementary Videos 3 and 4). Restriction of this structural rearrangement via the G174P mutation abolishes kinase activity (Supplementary Fig. 4b), thus underscoring the dual requirement for phosphorylation and loop reorganization for activation. The flexibility of the activation loop is a conserved regulatory principle in Hanks-type kinases^31,44,45^. Consequently, this may reflect an autoinhibitory strategy among PASTA-Hanks kinases, even if the molecular determinants of the loop captured in its self-inhibited conformation are likely to StkP.

Unexpectedly, both 2TA and 2TE structures displayed the αC helix in an “out” conformation, thereby precluding formation of the canonical K42–E61 salt bridge that is typically required for kinase activity. Indeed, the αC-out state is characteristic of inactive kinases and has been observed in inactive PknB mutants from *M. tuberculosis* and *S. aureus*^27,29,30^. The in vivo data confirms the significance of this salt bridge, as evidenced by the catalytically inactive and phenotypically indistinguishable state of the E61A mutant in comparison to the kinase-dead K42M mutant (Fig. 3). However, and intriguingly, StkP-2TE remains catalytically active in vivo, suggesting that conformational changes not captured in the crystal structure may facilitate formation of the K42–E61 salt bridge. Supporting this interpretation, our MD simulation results with AlphaFold3 models of ATP-bound StkP^287^−2TE and StkP^287^−2pT, as well as a PKA-based homology model of StkP^287^−2pT, demonstrate the feasibility and stability of an αC-in orientation that restores the catalytically crucial K42–E61 salt bridge (Fig. 4 and Supplementary Fig. 6). In fact, the structural overlay of the active WT StkP-2pT model onto the A and B chains of the X-ray crystal structure of StkP^287^−2TA allows visualization of the conformational switch that involves the coordinated motion of the activation loop and the αC helix (Fig. 4d). In the inactive state (chain A of the crystal structure), the αC-out conformation appears shifted away by 16.9° from the active state αC conformation in the doubly phosphorylated simulated structure (WT StkP-2pT), which displays a stable K42-E61 ionic lock. Remarkably, the αC helix in the also inactive chain B of the crystal structure is found midway through (angle = 7.5°), which implies that it may well constitute an intermediate between both conformational states. Taken together, these results reinforce the view that the αC helix behaves as an important allosteric element in StkP regulation, with the potential to integrate input from loop phosphorylation and the oligomerization state. In accordance with studies on PknB from mycobacteria^26,29^, it is thus proposed that the αC helix exists in a dynamic equilibrium between inactive and active states, and that its repositioning may require dimerization or other structural transitions.

Another key finding of this study is the identification of an unusual asymmetric N-lobe–C-lobe (NC) dimer interface that mediates a concentration-dependent dimerization of the StkP catalytic domain in solution (Fig. 5). This interface is distinct from the classical and conserved back-back (NN) and front-front (CC) interfaces described in PknB^26,29^ and restricted to streptococci (Supplementary Fig. 7). The back-to-back dimer is supposed to stabilize the αC helix in the active “in” conformation, thereby promoting pre-activation prior to loop phosphorylation^29^. Conversely, the front-to-front dimer facilitates transphosphorylation of the activation loops of two facing monomers^26^. However, our data show that only the NC interface is required to form dimers in solution (Fig. 5). Moreover, we observed that mutations affecting the NN and CC interfaces are the only ones to impair StkP kinase activity in vivo and to induce major cell division defects (Fig. 6). These observations imply that, although StkP does not form NN or CC dimers in vitro, both are likely to exist in vivo, potentially stabilized by interactions with other cellular partners. Interestingly, the analysis of the triple mutant (NC/CC/NN) reveals a phosphorylation pattern and a phenotype that are similar to those of the inactive CC mutant (Fig. 6), thereby confirming the prevailing role of the CC interface in catalytic activation and suggesting that the NC interface fulfills regulatory functions. In this context, the NC dimer may play a non-catalytic role, potentially constituting an inactive conformation required before StkP reorganization as active back-back (NN) and/or front-front (CC) dimers.

Unusual oligomeric forms of PknB have also been observed in *S. aureus* and *M. tuberculosis*, although their physiological relevance remains to be investigated in vivo. For instance, the crystallographic structure of the *S. aureus* PknB dimer reveals an αC-out autoinhibited state in *S. aureus*^30^, which may correspond to a comparable inactive scaffold as the NC interface of StkP. The identification of a physiologically relevant NC interface in StkP (Fig. 5) therefore expands the repertoire of kinase regulatory assemblies and suggests the existence of conceptually conserved, but structurally diverse, autoinhibitory dimers among PASTA-Hanks kinases. It can be further hypothesized that the inactive NC dimer must reorganize as an active dimer, potentially following interaction with an additional partner, to be fully active. This is supported by several observations made with eukaryotic-hanks-type kinases. Indeed, atypical dimers that inhibit activity have also been described^47–51^. For instance, GSK3β dimers occlude substrate-binding sites, representing an inactive form that is reversible by partner binding^49,50^. By analogy, we propose that the StkP NC dimer may represent a resting inactive state that prevents premature activation.

**Fig. 7:**
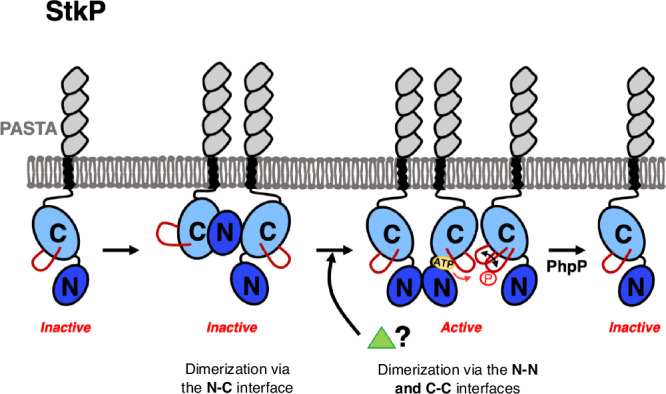
Model for StkP activation. StkP is depicted as in Fig. 1a. Accumulation of StkP at the septum increases its intracellular concentration, promoting the formation of an NC dimer. This dimer is inactive, and the activation loop adopts a stabilized “closed” conformation. Through interaction with an intracellular partner, the NC dimer would reorganize into active NN and CC dimers. Generation of the NN interface enables the allosteric activation of StkP, thereby resulting in the activation loop phosphorylation through the CC interface. Once phosphorylated, the activation loop adopts a dynamic “open” conformation that allows phosphorylation of StkP substrates. StkP activation would be potentially turned off by dephosphorylation of the activation loop by the StkP cognate phosphate PhpP.

Based on our data, we propose a revised model for StkP activation, in which the kinase initially forms an inactive, concentration-dependent dimer via the NC interface at the division septum. (Fig. 7). In this state, the activation loop adopts a defined organization that occludes the catalytic cleft, while the αC helix adopts an outward-shifted conformation that prevents salt bridge formation between the side chains of K42 and E61. This “poised” state may facilitate spatial concentration of StkP and provide a controlled environment for activation. Upon reaching a local concentration threshold, the NC dimer transitions toward the active NN conformation. This switch unlocks kinase activity through reorganization of the activation loop and the αC helix. In vivo, this transition may, however, require interaction with a cellular partner. Once formed, each monomer of the NN dimer would be activated upon stabilization of the αC helix in the active “in” conformation, and prompt to form additional dimers via the CC interface to allow trans-phosphorylation of the activation loop (Fig. 7). Each phosphorylated monomer, in which the activation loop does not obstruct the active site, would then be able to phosphorylate endogenous proteins. This model deviates from the proposed ligand-induced activation model of PASTA-Hanks kinases, in which PG fragments and/or lipid II binding to the extracellular domain trigger dimerization and activation^13,14^ but is consistent with several observations showing that PG binding is not a prerequisite for StkP activation^14,19,52^. Instead, our data suggest that intrinsic features of the kinase domain, such as dimer dynamics combined with loop phosphorylation, are the primary drivers of activation. This model is further substantiated by the ligand-independent activation of PrkC in *B. subtilis* ^20^and PknG in *M. tuberculosis*^24^. Overall, these observations suggest that this model may also apply to other PASTA-Hanks kinases involved in regulating the bacterial cell cycle, and that their activation may rely on interacting with cytoplasmic components rather than with extracellular ligands such as PG precursors or derivatives. In this respect, this model questions the ability of bacterial PASTA-Hanks kinases to function as membrane kinase receptors, suggesting instead that their extracellular domain may fulfill other functions, such as coordinating StkP activity with that of interacting partners like LytB and PBP2x^33,53^.

Our work underlines a dual regulatory mechanism for StkP activation, combining phosphorylation-induced loop restructuring with concentration-dependent dimerization via an unusual NC interface. This mechanism not only ensures tight control over kinase activation during cell division but also introduces an additional layer of spatial and structural regulation. Importantly, our data provide a framework for the exploration of analogous regulatory strategies in other PASTA-Hanks kinases across Gram-positive bacteria. Further investigations into how the NC interface coordinates and/or is formed with other cellular partners, will be critical. Comparative structural studies on StkP homologs in *S. mitis*, *S. agalactiae*, or *S. pyogenes*, in which the NC interface residues are conserved, may also prove useful in revealing the evolutionary conservation of this mechanism.

## Methods

### Strains and growth conditions

*S. pneumoniae* R800, wild type (WT) and mutants were grown in C + Y medium (pH 6.8) at 37 °C without shaking. Growth was tracked using a Tecan SUNRISE microtiter plate reader with a 550 nm filter. Cells were first cultured in C + Y medium (pH 6.8) to an OD550 of 0.1–0.2, then diluted to an OD550 of 0.0001 and transferred to a Nunc Edge 96-well, non-treated, flat- bottom MicroplateGreen plate. Growth was monitored at 37 °C, with OD readings taken every 10 min following brief shaking.

*E. coli* DH5-α and BL21 Star (DE3) strains were used for cloning and protein overexpression, respectively. Both strains were cultivated in Luria Bertani (LB) medium with appropriate antibiotics at 37 °C with shaking. The nucleotide sequence of all synthesized and mutated genes was checked to ensure proper base replacement and error-free amplification. All plasmids used in this study are listed in Supplementary Table 4.

### Allelic replacement mutagenesis and plasmid construction

To generate *S. pneumoniae* mutants, we used a two-step method involving the Janus bicistronic kan-rpsL cassette, as previously described^54^. Transformants were plated into THY-agar supplemented with 3% (vol/vol) defibrinated horse blood in the presence of appropriated antibiotics. All genetic modifications were performed at their native chromosomal loci in *S. pneumoniae*. DNA fragments for homologous recombination, flanked by ~0.5–1 kb of chromosomal DNA, were amplified by PCR from *S. pneumoniae* chromosomal DNA using primers listed in Supplementary Table 3. Each transformation included a negative control reaction without DNA. All strains are listed in Supplementary Table 4. For plasmid construction, DNA fragments encoding StkP mutant constructs were amplified by PCR using chromosomal DNA from the *S. pneumoniae* R800 strain as a template. The sequences of the primers and the description of the plasmids are provided in Supplementary Tables 3 and 4, respectively.

### Phase contrast and fluorescence microscopy

For microscopy, 0.5 µL of exponentially growing cells was deposited onto a 1% agarose C + Y pad on a microscope slide and sealed with a coverslip. Imaging was performed using a Nikon Ti-E/B microscope fitted with an Orca-CMOS Flash4 V2 camera and a 100×, 1.45 NA objective. Images were captured with NIS-Elements software (Nikon) and processed using ImageJ (http://rsb.info.nih.gov/ij/) with the MicrobeJ plugin^55^. Statistical analysis, based on Student’s t-tests, was conducted in triplicate using the MicrobeJ plugin.

### Preparation of S. pneumoniae crude extracts and immunoblot analysis

*S. pneumoniae* cultures were grown in C + Y medium (pH 6.8) to a specified OD550, then harvested by centrifugation and resuspended in 10 mM Tris-HCl (pH 8), 1 mM EDTA, with a protease and phosphatase inhibitor cocktail (Sigma, P5726). Following sonication to disrupt cells, 20 µg of crude extracts were separated by SDS-PAGE and transferred onto an Immobilon- P membrane (Millipore). Rabbit polyclonal primary antibodies were diluted as follows in TBST-BSA: anti-phosphothreonine (Cell Signaling Technology) at 1:2000 in 5% BSA, anti- StkP-PASTA at 1:100,000 in 5% BSA and anti-mGFP (Amsbio) at 1:5000 in 1% BSA^15^. The goat anti-rabbit HRP-conjugated secondary antibody (Bio-Rad) was used at 1:5000 in TBST with 1% BSA.

### Protein production and purification

The cytoplasmic domain of StkP, spanning residues 1–344 (StkP^344^−2TA) or 1–287 (StkP^287^- WT, StkP^287^−2TA, StkP^287^−2TE, StkP^287^-NC, StkP^287^-NN, StkP^287^-CC, and StkP^287^-NC/CC/NN) was encoded in the pEtPhos vector. These recombinant plasmids were transformed into *E. coli* BL21 Star (DE3). Cultures were grown in LB medium at 37 °C until reaching an OD600 of 0.6, and protein expression was induced with 1 mM IPTG for 3 h at 37 °C. Cells were harvested, resuspended in buffer A (25 mM Tris-HCl pH 8, 300 mM NaCl, 10% (v/v) glycerol, 10 mM imidazole) supplemented with 1 µg/mL lysozyme, 6 µg/mL DNase/RNase, and 1× Roche protease inhibitor, and disrupted using an M110P microfluidizer (Microfluidics) or sonication. After centrifugation at 15,000 × g for 30 min, the supernatant was applied to Ni-NTA agarose resin (Qiagen). Fractions containing the target protein were pooled and dialyzed overnight at 4 °C against 25 mM Tris pH 8, 300 mM NaCl, 1 mM DTT, and 0.5 mM EDTA, with the addition of solubility-enhanced hexahistidine-tagged S219V TEV protease at a 20:1 ratio^56^. The uncleaved protein and TEV protease were separated from the cleaved protein by a second purification step on a Ni- NTA agarose resin. The flowthrough, containing the cleaved protein, was concentrated and purified by size-exclusion chromatography on a HiLoad 16/600 Superdex 200 column (Cytiva) equilibrated in 25 mM Bis-Tris pH 7.4, 150 mM NaCl, except for StkP^344^−2TA (0.025 M Tris pH 7.5, 0.3 M NaCl, 10% glycerol). Pure protein fractions were pooled, concentrated, and assessed for purity by SDS-PAGE. Sample monodispersity was confirmed by dynamic light scattering (DLS) using a Zetasizer Nano S (Malvern Instruments) after centrifugation at 16,000 × *g* for 10 min at 4 °C. For long-term storage, proteins were flash- frozen in liquid nitrogen and kept at −80 °C.

### Crystallization and structure determination

Crystallization screening was carried out using a Mosquito workstation (STP Labtech) with commercial kits via the sitting-drop vapor diffusion method. All experiments were performed at 19 °C and observed with a RockImager 182 (Formulatrix).

StkP^344^−2TA (31 mg/mL) crystallized after 2 days in 12% PEG 4 K, 0.1 M MOPS pH 7 (MBClassII-E1), with crystals optimized using seeds in 11% PEG 4 K, 0.1 M MES pH 6.5. StkP^287^−2TA (10 mg/mL), supplemented with 1 mM AMP-PNP and 1 mM MgCl₂, crystallized after 3 days in 0.1 M HEPES pH 7.5, 10% (w/v) PEG 6 K, 5% (v/v) MPD (Crystal Screen-G6). StkP^287^−2TA (10 mg/mL), supplemented with 1 mM AMP-PNP and 1 mM MnCl₂, crystallized after 5 days in 20 %w/v PEG 3350, 0.1 M BIS-TRIS pH 6.5, 0.2 M KSCN (PactI-F4). StkP^287^−2TE (18 mg/mL) mixed with 1 mM AMP-PNP and 1 mM MnCl₂ formed crystals after 12 days in 8% (v/v) Tacsimate, 20% (w/v) PEG 3350 (PEG Ion-F7).

Crystals were cryoprotected by adding 20% (v/v) glycerol or ethylene glycol to their mother liquors and flash-frozen in liquid nitrogen. Diffraction data were collected at 100 K on beamlines ID29 at the ESRF synchrotron, PX1 and PX2A at the SOLEIL synchrotron and processed with the XDS package^57^. Space groups, asymmetric unit contents, and resolution details are listed in Supplementary Table 1.

Structures were solved by molecular replacement using BALBES for StkP^344^−2TA and Phaser in PHENIX^58^ for the others. The PDB entry 3DXN served as the starting model for StkP^344^−2TA, which was then used to solve the other four structures. Refinement proceeded through iterative cycles of COOT^59^ and PHENIX^58^ or Refmac5 in CCP4^60^. Structure quality was validated with MOLPROBITY^61^ prior to deposition in the Protein Data Bank under accession codes 9RU3 (StkP^344^−2TA- apo), 9RV7 (StkP^287^−2TA with AMP-PNP and Mg²⁺), 9RUK (StkP^287^−2TA with AMP-PNP and Mn²⁺), 9RUJ (StkP^287^−2TE with AMP-PNP and Mn²⁺). Data collection and refinement statistics are detailed in Supplementary Table 1. Sequence alignments and structural figures were created using ESPript^62^, UCSF ChimeraX^63^ and Pymol.

### Analytical ultracentrifugation

Sedimentation velocity experiments were performed on StkP^287^-WT at 0.2, 0.5, 1.5, 3, 4, 5 and 7 mg/ml, in 25 mM Bis-Tris pH 7.4, 150 mM NaCl, and on StkP^287^N*C, StkP^287^N*N, StkP^287^C*C and StkP^287^N*C/C*C/N*N, at 1, 5 and 10 mg/ml, in 25 mM Bis-Tris pH 7.4, 150 mM NaCl, 5% glycerol, at 4 °C and 42 000 rpm (130,000 × g). We used a Beckman XL-I analytical ultracentrifuge with a An-50 Ti or An-60 Ti rotor (Beckman Colter, USA). Samples of 400, 100 or 50 µl were loaded into two-channel titanium centerpieces of 12, 3 or 1.5 mm optical path-length, equipped with sapphire windows (Nanolytics, Germany). The buffer in the reference channel was the sample buffer. The acquisition was done every 8 min (except using the An-60 Ti, every 5 min), overnight, using absorbance at 280 nm and interference detection. Data processing and analysis were done using SEDFIT software v 16.36 (Schuck 2000), freely available at (https://sedfitsedphat.nibib.nih.gov/software), REDATE v 1.0.1^41^ and GUSSIv1.4.2^64^, freely available at (https://www.utsouthwestern.edu/research/core-facilities/mbr/software/). Sedimentation velocity profiles were globally analyzed in terms of *c*(*s*) distributions. The partial specific volume, refractive index increment, *dn/dc*, and extinction coefficient at 280 nm of StkP^287^-WT (0.731 ml/g, 0.189 ml/g and 0.644 ml/(mg cm), respectively), StkP^287^N*C (0.732 ml/g, 0.188 ml/g and 0.600 ml/(mg cm), respectively), StkP^287^N*N (0.732 ml/g, 0.189 ml/g and 0.600 ml/(mg cm), respectively), StkP^287^C*C (0.730 ml/g, 0.189 ml/g and 0.642 ml/(mg cm), respectively), StkP^287^N*C/C*C/N*N (0.730 ml/g, 0.189 ml/g and 0.600 ml/(mg cm), respectively), were estimated, at 4 °C, from the amino-acid sequence, using SEDFIT software v 16.36. The solvent density and viscosity of the buffer without glycerol and with glycerol (ρ = 1.007 and 1.021 g/ml, respectively, and η = 1.600 and 1.857 mPa s, respectively) were estimated using Sednterp software v 3.0.2 Beta, freely available at http://www.jphilo.mailway.com. For StkP WT samples, an isotherm analysis was done with the *s20w,mean* between 2 and 6S, using SEDPHAT v 15.2b, freely available at https://sedfitsedphat.nibib.nih.gov/software, in terms of A + A = A2 equilibrium, fixing Mw monomer = 32.4 kDa and *s20w* monomer = 3.0S.

### Small angle X-ray scattering (SAXS) data collection and processing

SAXS data were acquired at beamline BM29 of the ESRF synchrotron in Grenoble, France. StkP^287^-WT samples at concentrations of 1.3, 2.6, 5.2, 9.1, 10, and 13 mg/mL were centrifuged at 14,000 × *g* for 10 min prior to injection into the BM29 flow cell. Scattering data were collected at 4 °C, recording ten 1-second exposure frames^65^. Buffer background scattering was measured using the gel filtration buffer from sample purification. The EDNA pipeline at the beamline was used for data processing, including background subtraction, averaging, and scaling. ScÅtter IV^66^ was employed to calculate the pair distribution function P(r) and to determine Dmax and Rg from the scattering curves I(q) versus q in an automated, unbiased approach.

### Molecular dynamics simulations and modeling

The X-ray crystal structure StkP^287^−2TA in complex with AMP-PNP (ANP in the WWPDB Chemical Component Dictionary) and Mg^2+^ was used upon replacement of the ANP ligand with ATP. The RCD+ server^67^ was used to generate alternative conformations of the incomplete activation loop (amino acids 164−174) and select the three top-scoring ones as system replicates with distinct initial configurations. Replacement of A167 and A169 with either threonine or glutamic acid provided the corresponding initial models for StkP^287^−2T and StkP^287^−2TE, respectively. Additional starting structures for ATP-bound StkP^287^−2pT were generated with the AlphaFold 3^68^ and Swiss-Model^69^ servers, in this latter case using PDB entry 4HPU as the template (Supplementary Fig. 6 and Table 5). Energy minimization and unrestrained molecular dynamics (MD) simulations were run in explicit physiological saline solution under periodic boundary conditions, essentially as described^70^ using the *ff14SB* AMBER force field^71^ and updated parameters for phosphates^72^. The simulation protocol made use of the *pmemd.cuda_SPFP* engine implemented in AMBER 18^25^ running on Nvidia GTX 1080 RTX2080Ti GPUs. In brief, the SHAKE algorithm was applied to all bonds involving hydrogens and an integration step of 2.0 fs was used. The cutoff distance for the non-bonded interactions was 9 Å and electrostatic interactions were represented using the smooth particle mesh Ewald method with a grid spacing of 1 Å. Following energy minimization to remove any steric clashes, TIP3P water molecules and counterions were allowed to redistribute around the positionally restrained solute (5 kcal mol^−1^Å^−2^) during a heating phase from 100 to 310 K (0.1 ns) followed by equilibration at 310 K (2.5 ns). Each system was then simulated in the absence of any restraints for 300 ns during which system coordinates were collected every 5 ns for further analysis. For completeness, *in crystallo* simulations of chains A and B of StkP^344^−2TA (in the presence of symmetry mates C and D) were also run with either AMP-PNP:2Mg^2+^ or ATP:2Mg^2+^ bound in the active site (Supplementary Fig. 6 and Supplementary Table 5) using previously reported simulation conditions^73^. Root-mean-square fluctuations per residue were calculated with the aid of the *cpptraj* module in AmberTools^74^. The molecular graphics program PyMOL v. 2.6 (https://pymol.org) was employed for molecular visualization and figure preparation, and the AngleBetweenHelices.py plugin was used to calculate the angle between helices shown in Fig. 4d.

### Reporting summary

Further information on research design is available in the Nature Portfolio Reporting Summary linked to this article.

## Supplementary information

Supplementary Information

Description of Additional Supplementary File

Supplementary Video 1

Supplementary Video 2

Supplementary Video 3

Supplementary Video 4

Supplementary Video 5

Reporting Summary

Transparent Peer Review file

## Source data

Source data

## Data Availability

Coordinates for the cytoplasmic catalytic domain structures of *S. pneumoniae* StkP have been deposited into the Protein Data Bank under accession numbers 9RU3 (structure of StkP^344^−2TA in the apo state), 9RV7 (structure of StkP^287^−2TA with AMP-PNP and Mg²⁺), 9RUK (structure StkP^287^−2TA with AMP-PNP and Mn²⁺), 9RUJ (structure of StkP^287^−2TE with AMP-PNP and Mn²⁺). PDB 3DXN served as a starting model for structure determination. PDB 4HPU was used as a template in molecular modeling. PDB 1MRU), 3ORM, 4EQM) and 3F69 were used for structural comparisons. The source data underlying the Figures and the Supplementary Figs. and Tables are provided as Source Data files and in the Supplementary Information. As microscopy images represent a large amount of data, they are available from the corresponding authors upon request. Source data are provided with this paper.
