# Supplementary material for: Structural dissection of the catalytic domain of the serine threonine kinase StkP of Streptococcus pneumoniae
Source: Nat Commun. 2026 Jun 18;17:7687. doi: 10.1038/s41467-026-74470-6 (PMC13434768; doi:10.1038/s41467-026-74470-6)

SUPPLEMENTARY INFORMATION

Structural dissection of the catalytic domain of the serine threonine kinase StkP of *Streptococcus pneumoniae*

Mélisse Hamidi[1], Virginie Gueguen-Chaignon[2], Cassandra Falcou[1], Hugo Millat[1], Sathya Narayanan Nagarajan[1], Aline Le Roy[3], Céline Freton[1], Federico Gago[4], Stéphanie Ravaud[1, ‡] and Christophe Grangeasse[1, ‡, *]

Supplementary Figures and legends 1 to 9

Supplementary Tables 1 to 5

Supplementary References

Source Data Supplementary Figures 2a, 2c, 3a, 4b, 9b

Supplementary Figure 1. Structural features of the catalytic domain of bacterial Hanks-type kinases.

(**a**) Sequence alignment of *S. pneumoniae* StkP with *M. tuberculosis* PknB, *B. subtilis* PrkC, and *S. aureus* PknB. The secondary structure derived from the X-ray structure of $StkP^{287}$-2TA is shown above. The position of Hanks motifs is highlighted in: pink (I), blue (II), dark gray (V), yellow (VI), purple (VII), red (activation loop), green (VIII), orange (X), and dark blue (XI). The two phosphorylatable threonine residues in the activation loop, T167 and T169, are marked by red asterisks. The figure was generated using ESPript (https://espript.ibcp.fr/). (**b**) Cartoon representation of $StkP^{287}$-2TA showing the N- and C-lobes and highlighting the positions of the Hanks motifs with the same color coding as in (a).

a

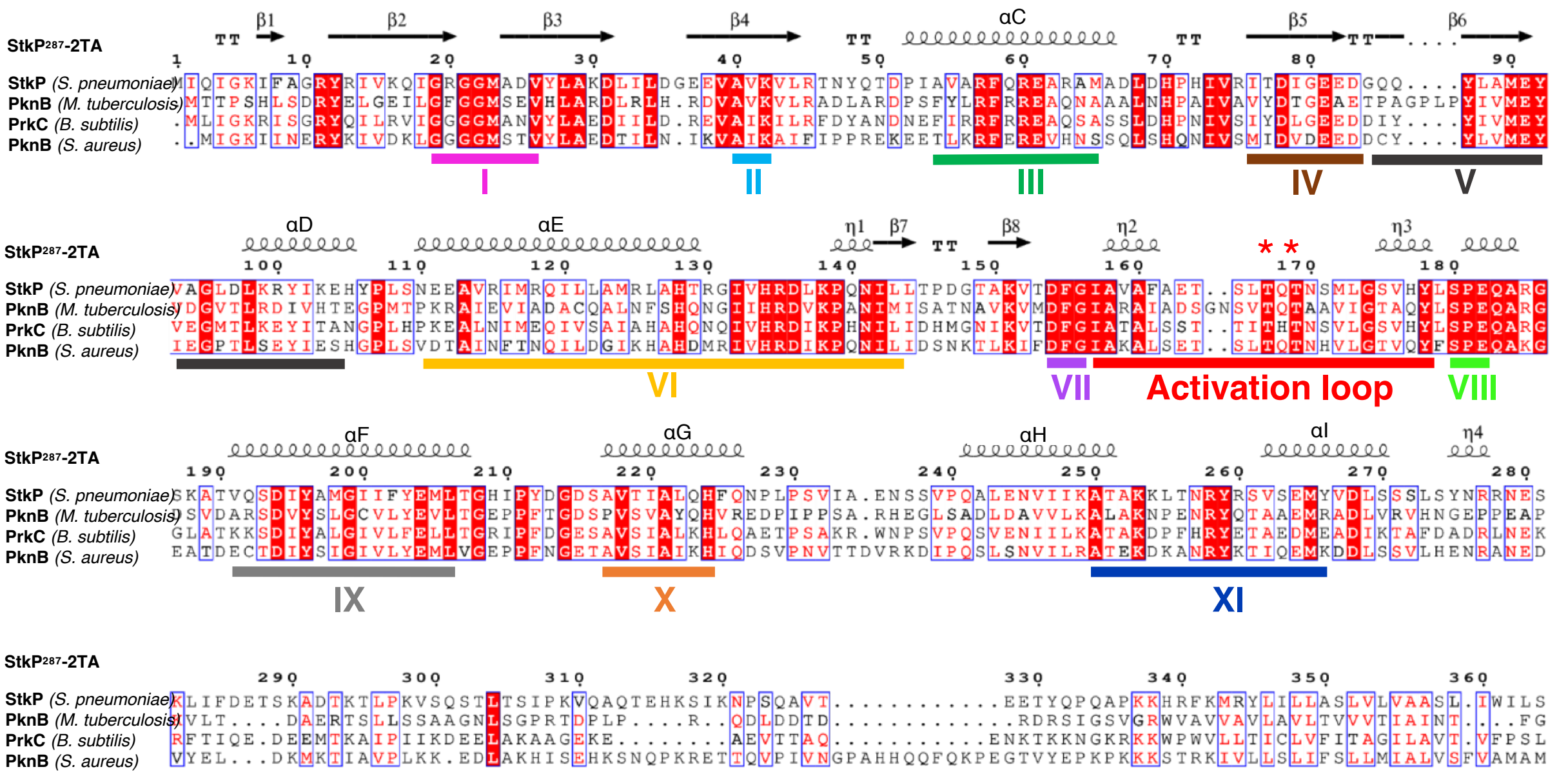
StkP287-2TA
StkP (S. pneumoniae)
PknB (M. tuberculosis)
PrkC (B. subtilis)
PknB (S. aureus)
I
II
III
IV
V
VI
VII
Activation loop
VIII
IX
X
XI

b

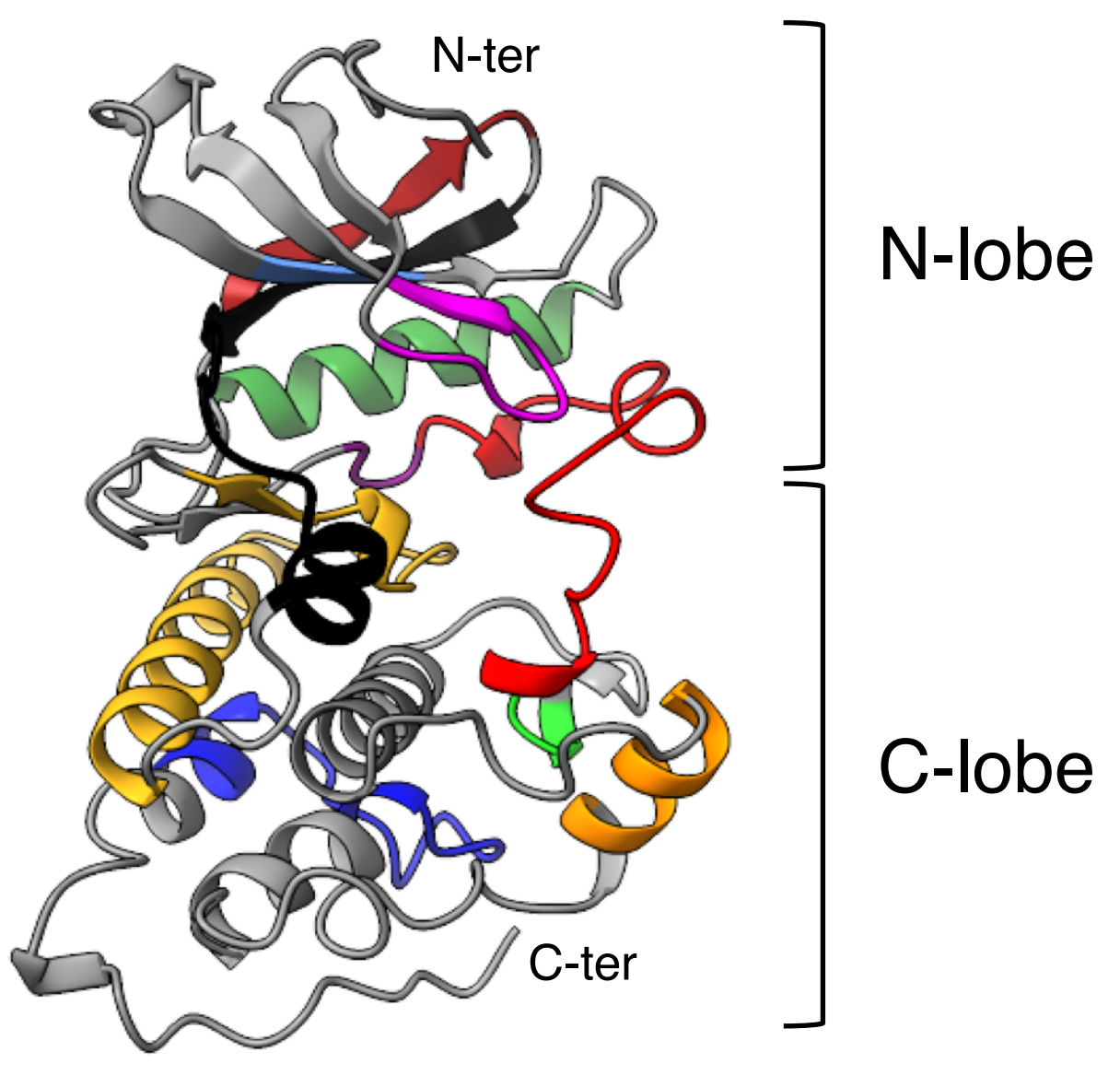
N-ter
N-lobe
C-lobe
C-ter

Supplementary Figure 2. Impact of StkP mutations and mGFP fusion on phosphorylation patterns and cell growth.

(**a**) Western immunoblot of whole-cell lysates from *WT*, *stkP-2TA, stkP-2TE,* and *stkP-K42M* cells. Cells were probed with anti-StkP PASTA domain antibodies showing that StkP mutated forms are properly produced. (**b**) Growth of *WT*, *stkP-2TA, stkP-2TE,* and *stkP-K42M* strains. Strains were grown in C + Y medium at pH 6,8 and at 37°C. (**c**) Expression of m*gfp-stkP*, m*gfp-stkP-2TA* and m*gfp-stkP-2TE* in the pneumococcus. The Western immunoblot was probed with anti-mGFP antibodies (α-GFP). (**d**) Growth of m*gfp-stkP*, m*gfp-stkP-2TA* and m*gfp-stkP-2TE* strains. Strains were grown as indicated in (b). (**e**) Growth of *WT*, *stkP-K42M*, *stkP-D136N, stkP-D154A,* and *stkP-E61A* strains. Strains were grown as indicated in (b). All experiments were performed in triplicate.

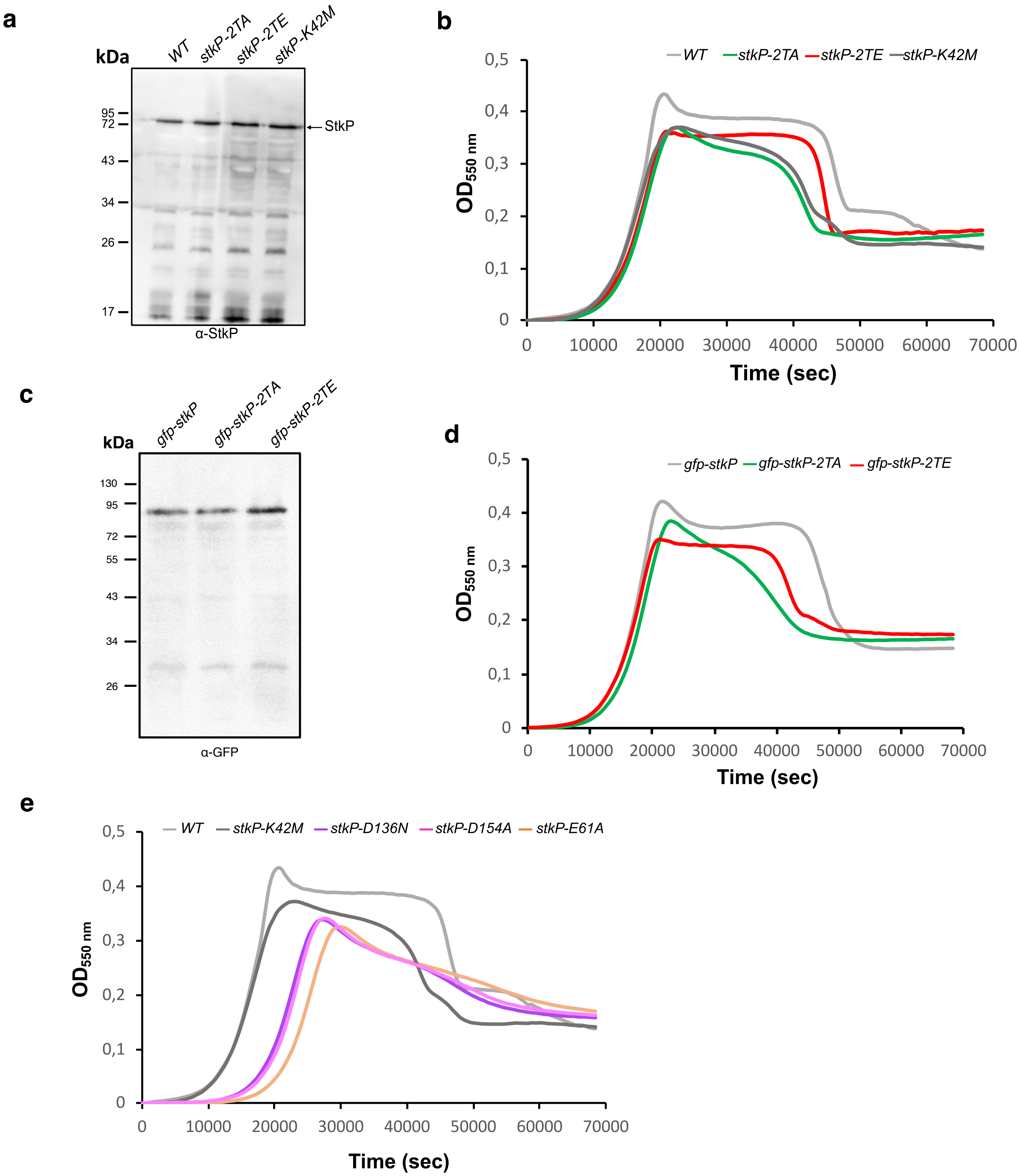
a
kDa
WT
stkP-2TA
stkP-2TE
stkP-K42M
95
72
43
34
26
17
StkP
α-StkP
b
WT
stkP-2TA
stkP-2TE
stkP-K42M
OD550 nm
0,5
0,4
0,3
0,2
0,1
0
0
10000
20000
30000
40000
50000
60000
70000
Time (sec)
c
kDa
gfp-stkP
gfp-stkP-2TA
gfp-stkP-2TE
130
95
72
55
43
34
26
α-GFP
d
gfp-stkP
gfp-stkP-2TA
gfp-stkP-2TE
OD550 nm
Time (sec)
e
WT
stkP-K42M
stkP-D136N
stkP-D154A
stkP-E61A
OD550 nm
Time (sec)

Supplementary Figure 3. Purification, crystallization and structure of StkP mutants.

(**a**) Purification of the StkP samples used in the X-ray crystallography study. After overproduction in *E. coli* cells and purification, all the proteins were analyzed by SDS-PAGE. (**b**) Cartoon representation of the asymmetric unit of $StkP^{344}$-2TA structure (molecules A and B) (left panel). The right panel shows the superimposition of molecules A and B. (**c**) Cartoon representation of the asymmetric unit of $StkP^{287}$-2TA structure (molecules A and B) (left panel). The right panel shows the superimposition of molecules A and B. (**d**) Superimposition of molecule B of $StkP^{344}$-2TA (in light orange) on the molecule B of $StkP^{287}$-2TA (in teal).

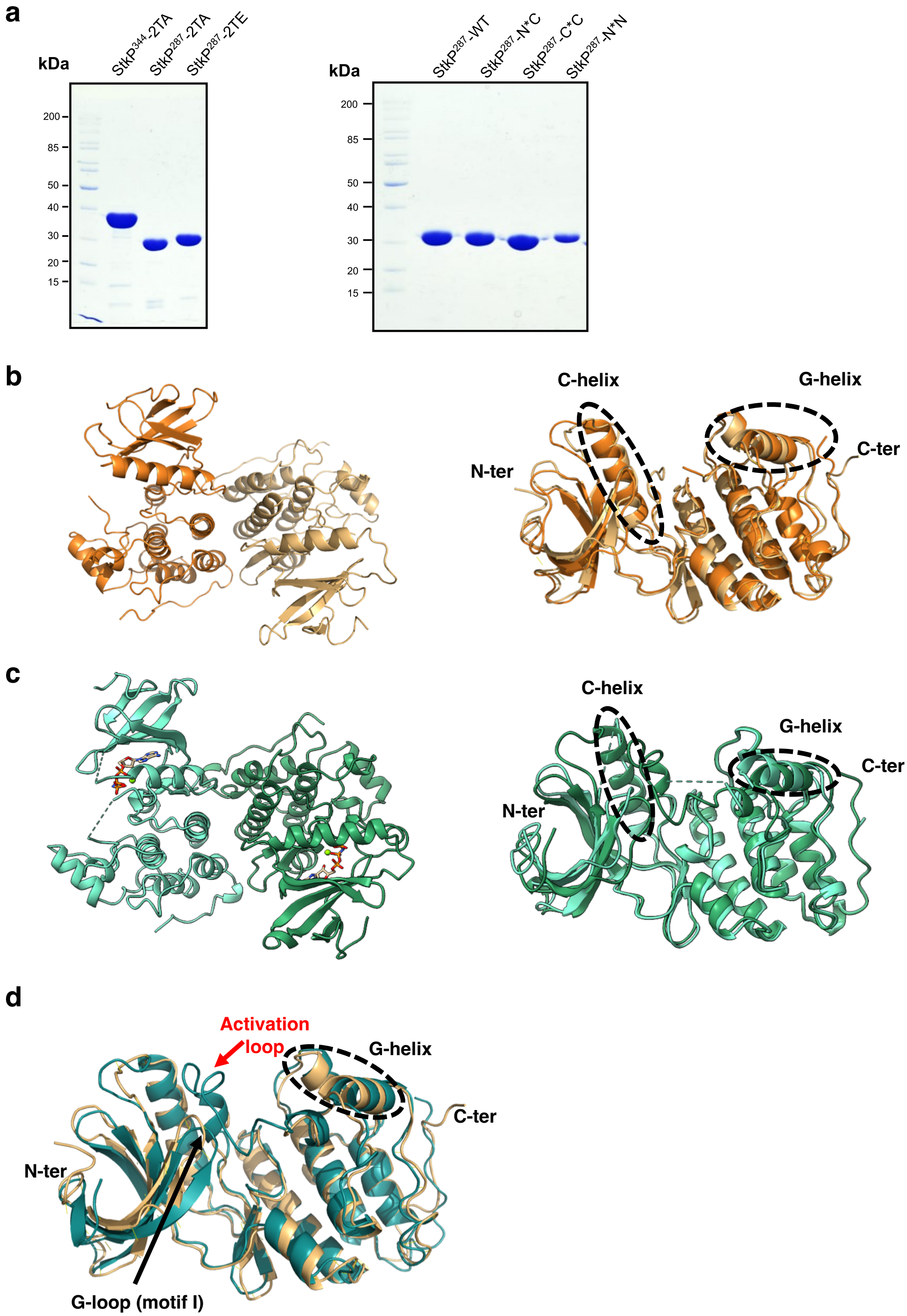

a
kDa
StkP344-2TA
StkP287-2TA
StkP287-2TE
200
85
50
40
30
20
15
kDa
StkP287-WT
StkP287-N*C
StkP287-C*C
StkP287-N*N
200
85
50
40
30
20
15
b
C-helix
G-helix
N-ter
C-ter
c
C-helix
G-helix
N-ter
C-ter
d
Activation loop
G-helix
C-ter
N-ter
G-loop (motif I)

Supplementary Figure 4. Characterization of the G174P mutant.

(a) Growth of WT and *stkP-G174P* strains. Strains were grown in C + Y medium at pH 6.8 and at 37°C. Experiments were performed in triplicate. **(b)** Phosphorylation patterns. Western immunoblots of whole-cell lysates from WT, *stkP-G174P* and *stkP-K42M* cells. Cells were probed with anti-phosphothreonine antibodies. StkP substrates (MapZ, DivIVA, EloR and MacP) are listed in black and StkP is shown in red. Images are representative of experiments made in triplicate.

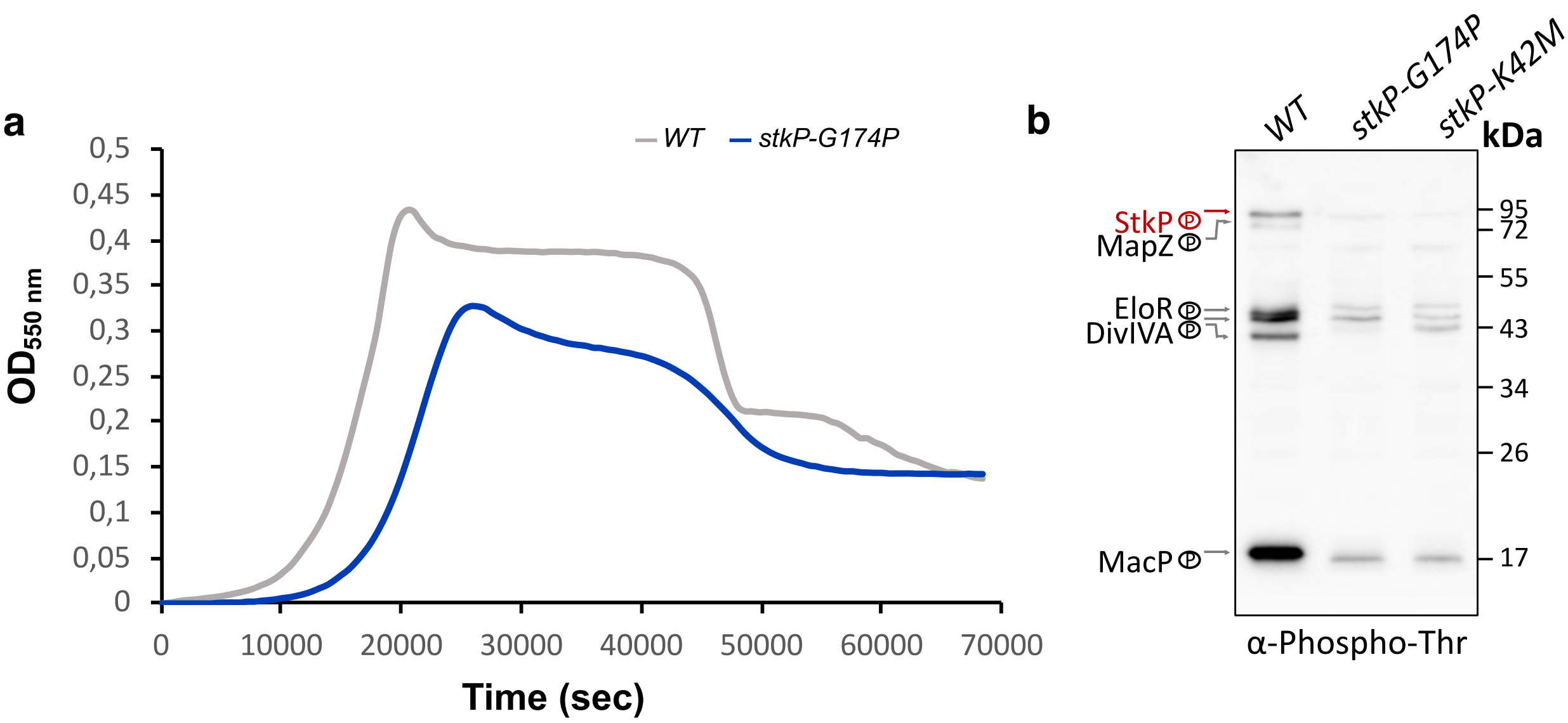
a
WT
stkP-G174P
OD550 nm
Time (sec)
0,5
0,45
0,4
0,35
0,3
0,25
0,2
0,15
0,1
0,05
0
10000
20000
30000
40000
50000
60000
70000
b
WT
stkP-G174P
stkP-K42M
kDa
StkP
MapZ
EloR
DivIVA
MacP
95
72
55
43
34
26
17
α-Phospho-Thr

Supplementary Figure 5. Structure of the StkP^{287}-2TE form.

(**a**) Cartoon representation of the asymmetric unit of the StkP^{287}-2TE crystal structure (molecules A (pink) and B (red)). The partially resolved activation loop is indicated (dotted rectangle) in the middle panel (molecule B only). The left panel shows the superimposition of molecule B of StkP^{287}-2TE (red) on the molecule B of StkP^{287}-2TA (in teal). A zoom-in on the αC helix in the N-terminal lobe of StkP$_{287}$-2TE focuses on residues K42 and E61, indicating the absence of the salt bridge. (**b**) Comparison of the X-ray crystal structure of StkP^{287}-2TE bound to AMP-PNP and a Mn^{2+} ion (left) with the Alphafold 3 model of StkP^{287}-WT bound to ATP and Mg^{2+} (right). The quality of the AlphaFold3 model, including the predicted local distance difference test (pLDDT) is given in Supplementary Figure 6. Note the differences in the loop comprising residues T46-T50 and in the activation loop containing T167 and T169 which is only visible in the latter and places S171 in a distinct location relative to the nucleotide phosphates.

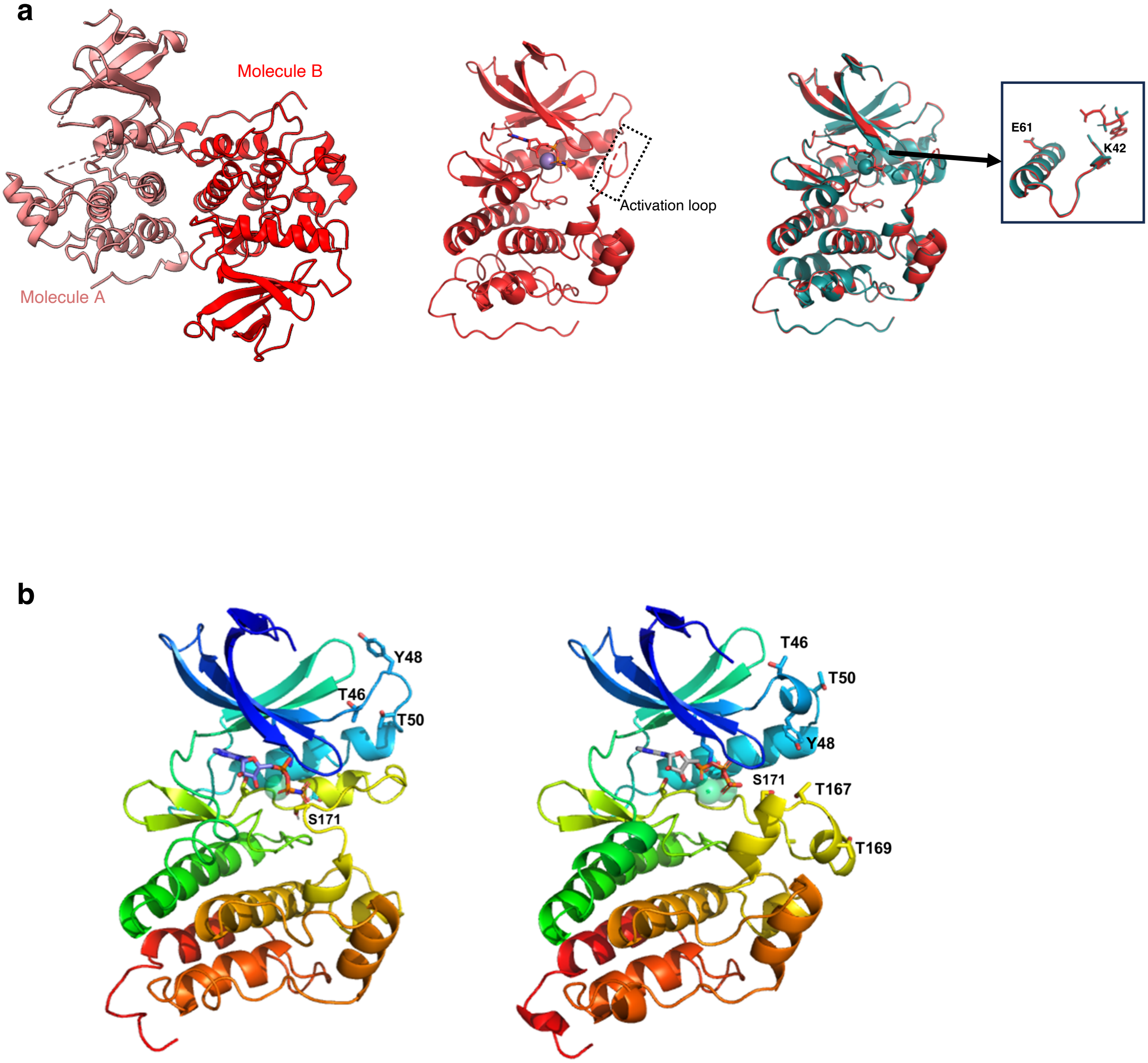

a
Molecule B
Molecule A
Activation loop
E61
K42
b
Y48
T46
T50
S171
T46
T50
Y48
S171
T167
T169

Supplementary Figure 6. SwissModel and AlphaFold 3 models used in the molecular dynamic simulations.

(a) Initial structure provided by SwissModel for $StkP^{287}$-2TA using the X-ray crystal structure as template**. (b)** AlphaFold 3 model for WT $StkP^{287}$:ATP:2Mg^{2+}. **(c)** Initial structure provided by SwissModel for $StkP^{287}$-2T using PDB entry 4HPU as template. **(d)** AlphaFold 3 model for WT $StkP^{287}$-2TPO:ATP:2Mg^{2+}. **(e)** Initial structure provided by AlphaFold 3 for $StkP^{287}$-2TE:ATP:2Mg^{2+}. (**f**) AlphaFold 3 model for $StkP^{287}$-2TD:ATP:2Mg^{2+}.

a

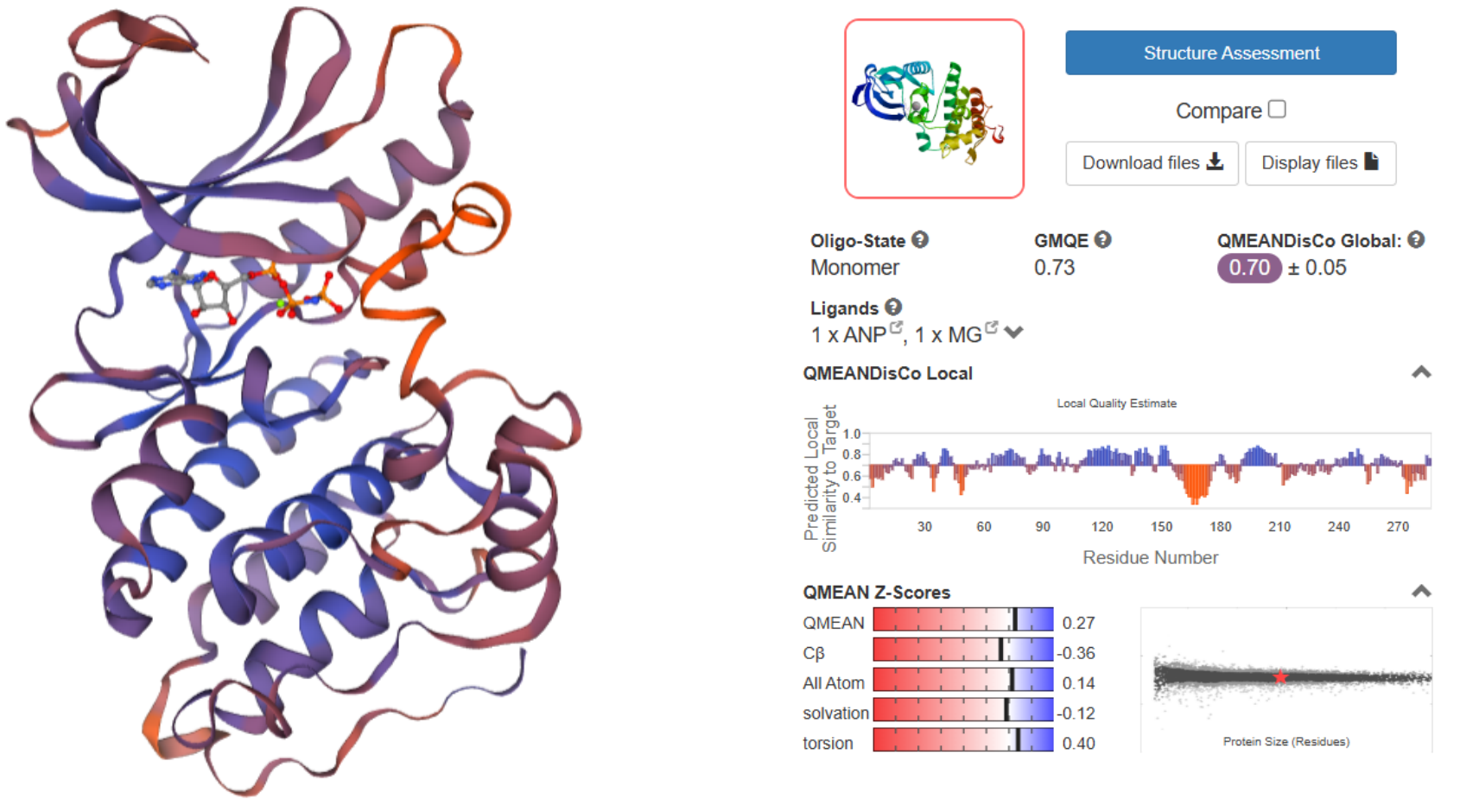

Structure Assessment
Compare
Download files
Display files
Oligo-State
Monomer
GMQE
0.73
QMEANDisCo Global:
0.70 ± 0.05
Ligands
1 x ANP, 1 x MG
QMEANDisCo Local
Local Quality Estimate
Predicted Local Similarity to Target
Residue Number
QMEAN Z-Scores
QMEAN 0.27
Cβ -0.36
All Atom 0.14
solvation -0.12
torsion 0.40
Protein Size (Residues)

b

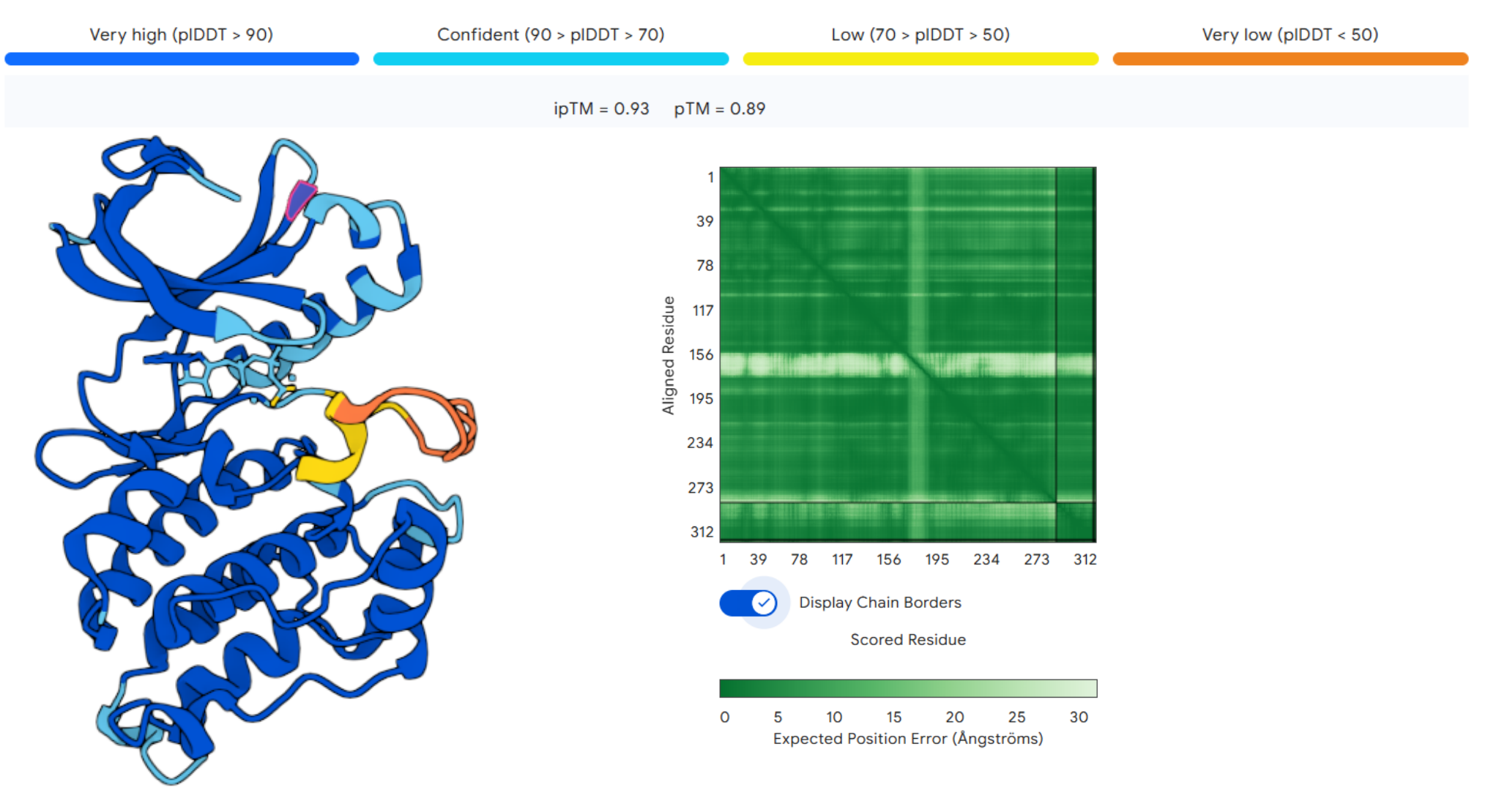

Very high (plDDT > 90)
Confident (90 > plDDT > 70)
Low (70 > plDDT > 50)
Very low (plDDT < 50)
ipTM = 0.93 pTM = 0.89
Aligned Residue
Display Chain Borders
Scored Residue
Expected Position Error (Ångströms)

c

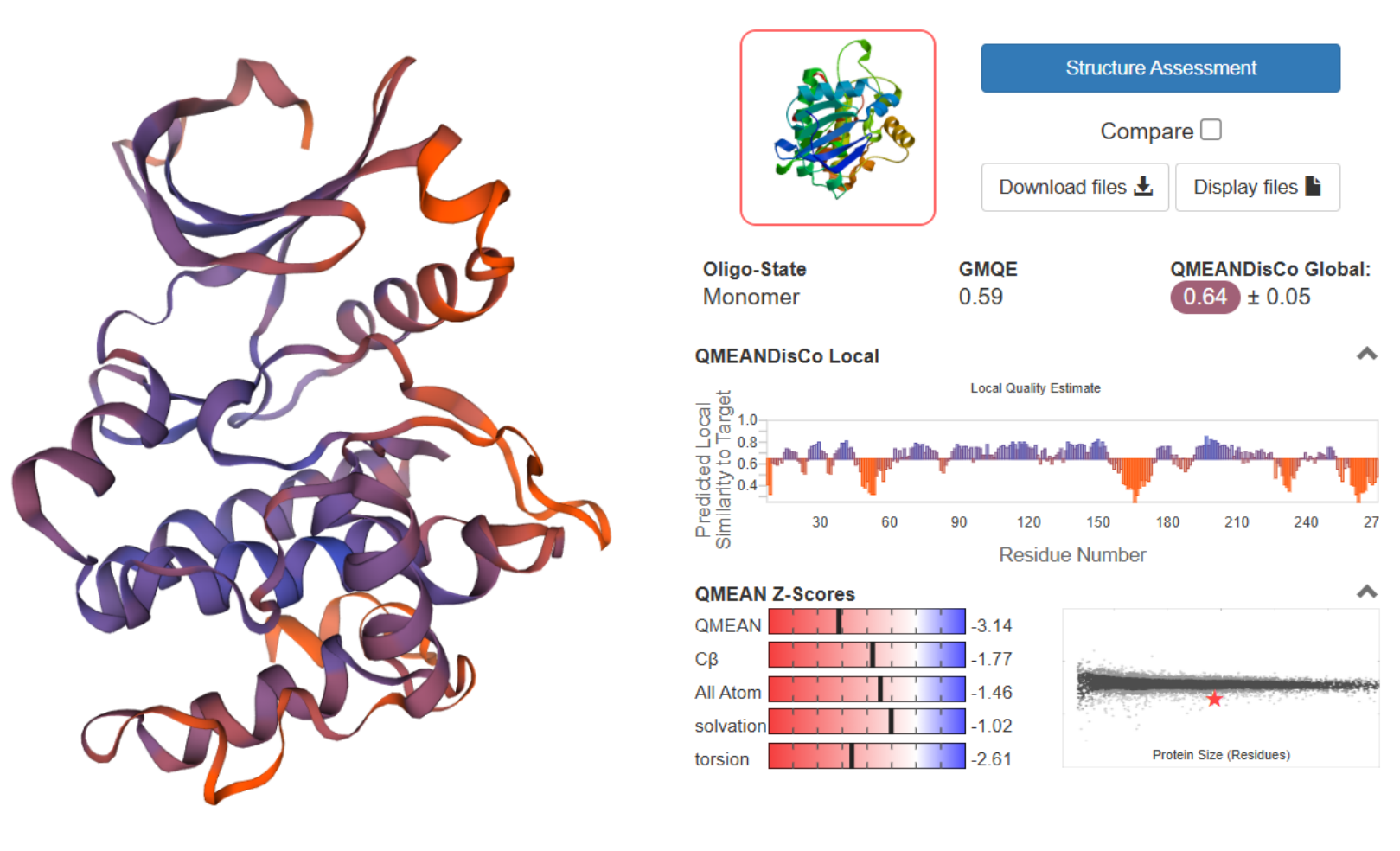

Structure Assessment
Compare
Download files
Display files
Oligo-State
Monomer
GMQE
0.59
QMEANDisCo Global:
0.64 ± 0.05
QMEANDisCo Local
Local Quality Estimate
Predicted Local Similarity to Target
Residue Number
QMEAN Z-Scores
QMEAN -3.14
Cβ -1.77
All Atom -1.46
solvation -1.02
torsion -2.61
Protein Size (Residues)

d

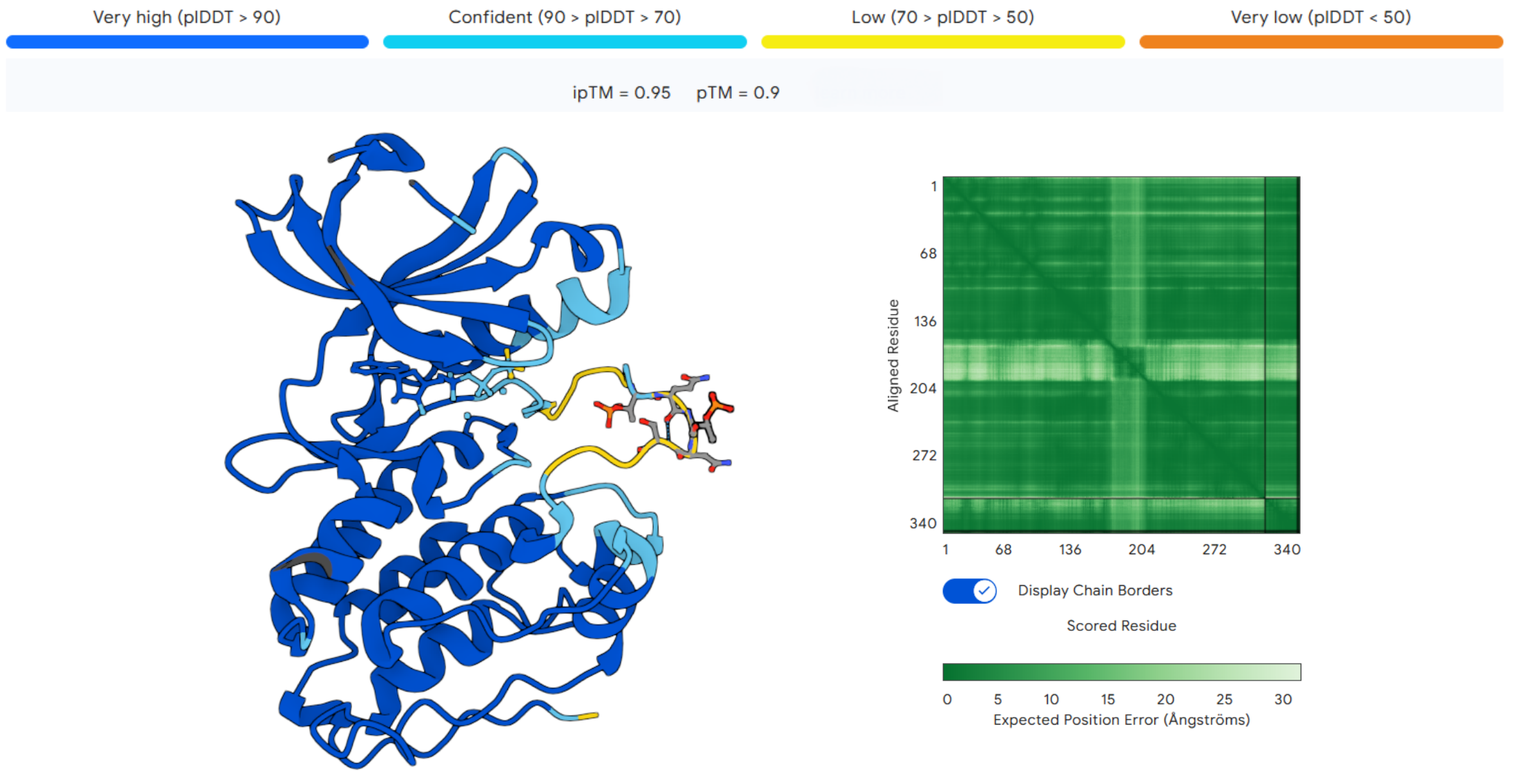

Very high (pIDDT > 90)
Confident (90 > pIDDT > 70)
Low (70 > pIDDT > 50)
Very low (pIDDT < 50)
ipTM = 0.95 pTM = 0.9
Aligned Residue
1
68
136
204
272
340
Display Chain Borders
Scored Residue
0 5 10 15 20 25 30
Expected Position Error (Ångströms)

e

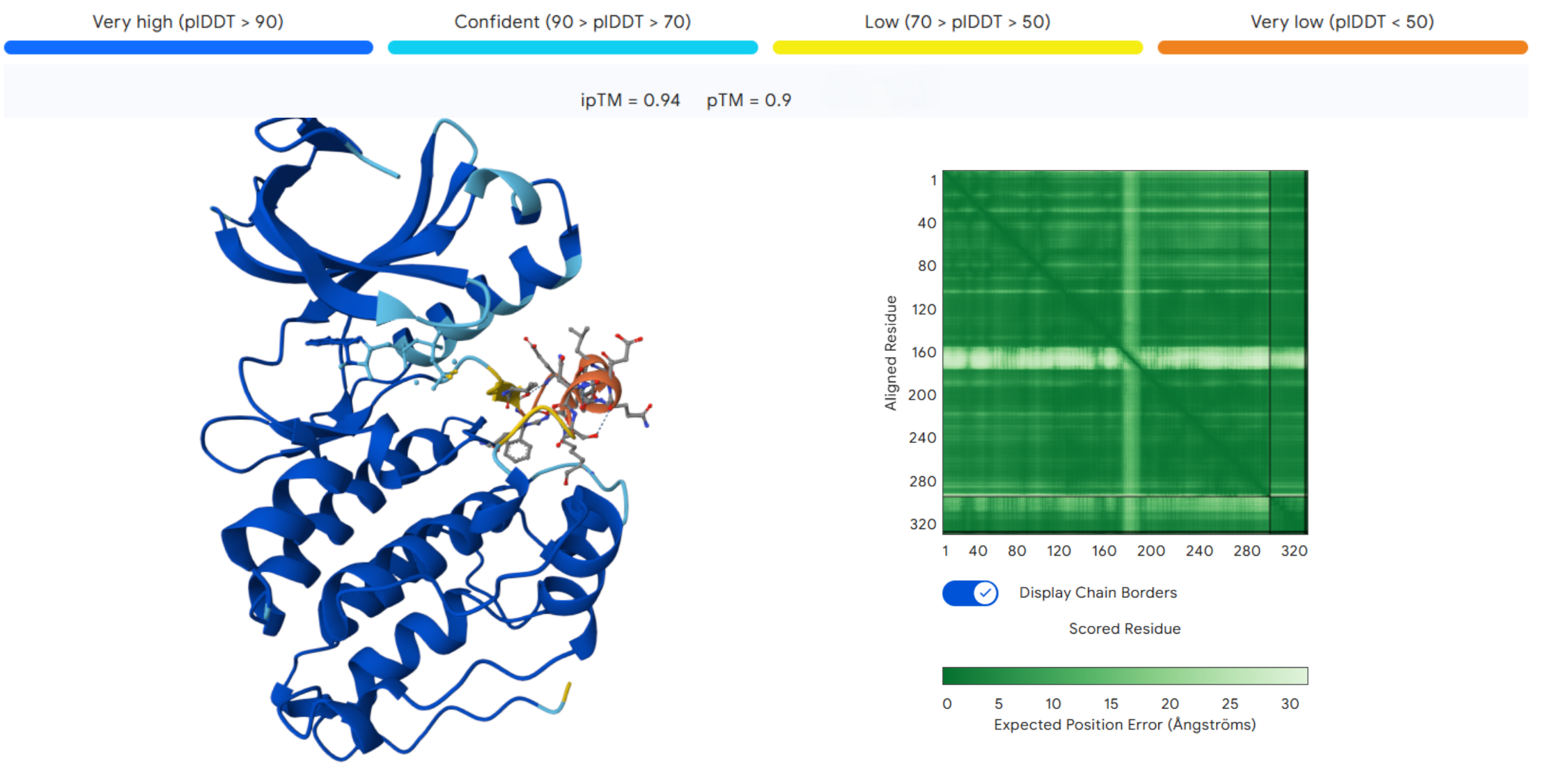

Very high (pIDDT > 90)
Confident (90 > pIDDT > 70)
Low (70 > pIDDT > 50)
Very low (pIDDT < 50)
ipTM = 0.94 pTM = 0.9
Aligned Residue
1
40
80
120
160
200
240
280
320
Display Chain Borders
Scored Residue
0 5 10 15 20 25 30
Expected Position Error (Ångströms)

f

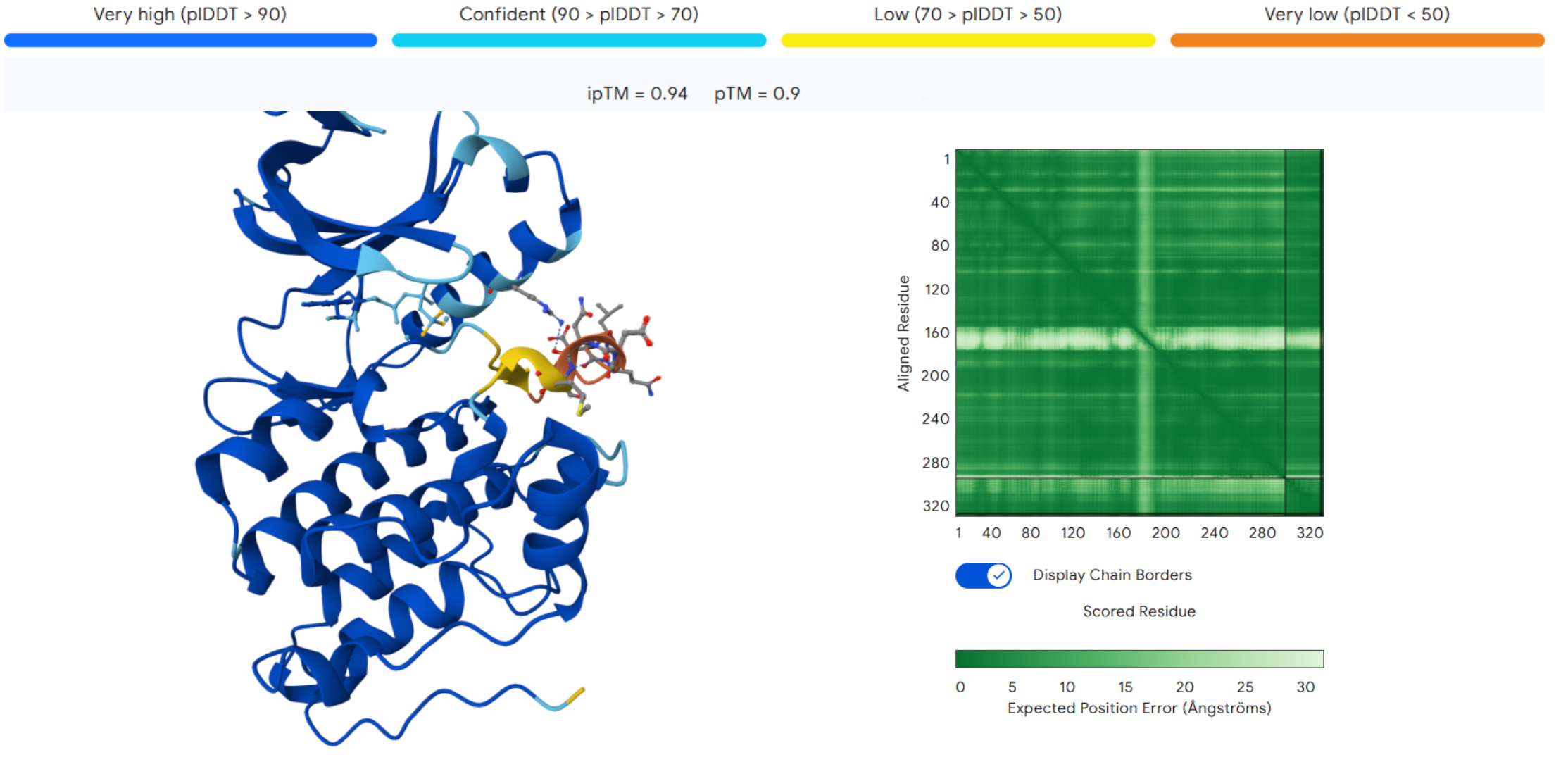

Very high (pIDDT > 90)
Confident (90 > pIDDT > 70)
Low (70 > pIDDT > 50)
Very low (pIDDT < 50)
ipTM = 0.94 pTM = 0.9
Aligned Residue
1
40
80
120
160
200
240
280
320
Display Chain Borders
Scored Residue
0 5 10 15 20 25 30
Expected Position Error (Ångströms)

Supplementary Figure 7. Sequence conservation of the catalytic domain of Streptococcal StkP homologs.

(a) Sequence alignment indicating with asterisks the positions of the residues D69, H72, R125 and Y266 involved in the NC interface. **(b)** Surface and cartoon representations of the catalytic domain of StkP (StkP^{287}-2TA, molecule B of the asymmetric unit) colored according to sequence conservation as in (a). The protein is depicted in two different orientations as in N-lobe–C-lobe interface. The positions of the residues involved in the NC interface and marked with asterisks in (a) are indicated.

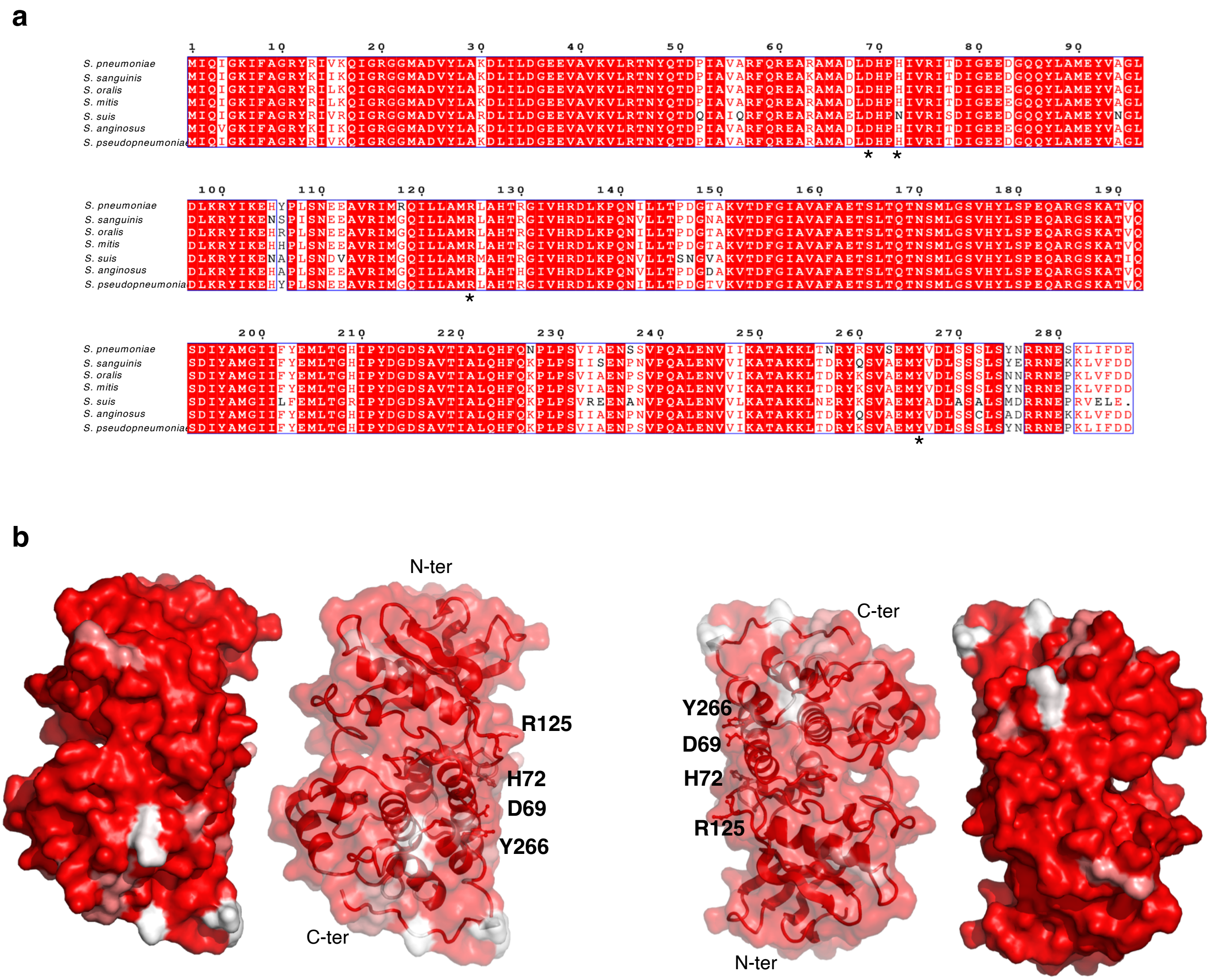
a
1 10 20 30 40 50 60 70 80 90
S. pneumoniae MIQIGKIFAGRYRIVKQIGRGGMADVYLAKDLILDGEEVAVKVLRTNYQTDPIAVARFQREARAMADLDHPHIVRITDIGEEDGQQYLAMEYVAGL
S. sanguinis MIQIGKIFAGRYKIIKQIGRGGMADVYLAKDLILDGEEVAVKVLRTNYQTDPIAVARFQREAKAMADLDHPHIVRITDIGEEDGQQYLAMEYVAGL
S. oralis MIQIGKIFAGRYRILKQIGRGGMADVYLAKDLILDGEEVAVKVLRTNYQTDPIAVARFQREARAMADLDHPHIVRITDIGEEEGQQYLAMEYVAGL
S. mitis MIQIGKIFAGRYRILKQIGRGGMADVYLAKDLILDGEEVAVKVLRTNYQTDPIAVARFQREARAMAELDHPHIVRITDIGEEEGQQYLAMEYVAGL
S. suis MIQIGKIFAGRYRIVRQIGRGGMADVYLARDLILDGEEVAVKVLRTNYQTDQIAIQRFQREARAMAELDHPNIVRISDIGEEDGQQYLAMEYVNGL
S. anginosus MIQVGKIFAGRYKIIKQIGRGGMADVYLAKDLILDGEEVAVKVLRTNYQTDPIAVARFQREARAMADLDHPHIVRITDIGEEEGQQYLAMEYVAGL
S. pseudopneumoniae MIQIGKIFAGRYRIVKQIGRGGMADVYLAKDLILDGEEVAVKVLRTNYQTDPIAVARFQREARAMADLDHPHIVRITDIGEEDGQQYLAMEYVAGL
100 110 120 130 140 150 160 170 180 190
S. pneumoniae DLKRYIKEHYPLSNEEAVRIMRQILLAMRLAHTRGIVHRDLKPQNILLTPDGTAKVTDFGIAVAFAETSLTQTNSMLGSVHYLSPEQARGSKATVQ
S. sanguinis DLKRYIKENSPISNEEAVRIMGQILLAMRLAHTRGIVHRDLKPQNVLLTPDGNAKVTDFGIAVAFAETSLTQTNSMLGSVHYLSPEQARGSKATVQ
S. oralis DLKRYIKEHRPLSNEEAVRIMGQILLAMRLAHTRGIVHRDLKPQNILLTPDGTAKVTDFGIAVAFAETSLTQTNSMLGSVHYLSPEQARGSKATVQ
S. mitis DLKRYIKEHHPLSNEEAVRIMGQILLAMRLAHTRGIVHRDLKPQNILLTPDGTAKVTDFGIAVAFAETSLTQTNSMLGSVHYLSPEQARGSKATVQ
S. suis DLKRYIKENAPLSNDVAVRIMGQILLAMRMAHTRGIVHRDLKPQNVLLTSNGVAKVTDFGIAVAFAETSLTQTNSMLGSVHYLSPEQARGSKATIQ
S. anginosus DLKRYIKEHAPLSNEEAVRIMGQILLAMRLAHTHGIVHRDLKPQNVLLTPDGDAKVTDFGIAVAFAETSLTQTNSMLGSVHYLSPEQARGSKATVQ
S. pseudopneumoniae DLKRYIKEHYPLSNEEAVRIMGQILLAMRLAHTRGIVHRDLKPQNILLTPDGTVKVTDFGIAVAFAETSLTQTNSMLGSVHYLSPEQARGSKATVQ
200 210 220 230 240 250 260 270 280
S. pneumoniae SDIYAMGIIFYEMLTGHIPYDGDSAVTIALQHFQNPLPSVIAENSSVPQALENVIIKATAKKLTNRYRSVSEMYVDLSSSLSYNRRNESKLIFDE
S. sanguinis SDIYAMGIIFYEMLTGHIPYDGDSAVTIALQHFQKPLPSIISENPNVPQALENVVIKATAKKLTDRYQSVAEMYVDLSSSLSYERRNEKKLVFDD
S. oralis SDIYAMGIIFYEMLTGHIPYDGDSAVTIALQHFQKPLPSVIAENPSVPQALENVVIKATAKKLTDRYKSVAEMYVDLSSSLSNNRRNEPKLVFDD
S. mitis SDIYAMGIIFYEMLTGHIPYDGDSAVTIALQHFQKPLPSVIAENPSVPQALENVVIKATAKKLTDRYKSVAEMYVDLSSSLSYNRRNEPKLVFDD
S. suis SDIYAMGIILFEMLTGRIPYDGDSAVTIALQHFQKPLPSVREENANVPQALENVVLKATAKKLNERYKSVAEMYADLASALSMDRRNEPRVELE.
S. anginosus SDIYAMGIIFYEMLTGHIPYDGDSAVTIALQHFQKPLPSIIAENPNVPQALENVVIKATAKKLTDRYQSVAEMYVDLSSCLSADRRNEKKLVFDD
S. pseudopneumoniae SDIYAMGIIFYEMLTGHIPYDGDSAVTIALQHFQKPLPSVIAENPSVPQALENVVIKATAKKLTDRYKSVAEMYVDLSSSLSYNRRNEPKLIFDD
b
N-ter
R125
H72
D69
Y266
C-ter
C-ter
Y266
D69
H72
R125
N-ter

Supplementary Figure 8. AUC analysis of WT StkP and dimerization mutants.

(**a**) Superposition of selected experimental (dot) and fitted (line) sedimentation velocity profiles acquired in absorbance at 280 nm, during ≈ 14h30 at 130 000 g (top), and residuals (bottom), for StkP^{287}-WT at 3 mg/ml, measured at 4°C, in a cell of 0.15 cm optical path length. (**b**) Normalized *c* ($s_{20,w}$) distributions obtained at 280 nm, at 4°C, for StkP^{287}-WT at 0.2 (magenta), 0.5 (purple), 1.5 (blue), 3 (green), 4 (yellow), 5 (orange) and 7 (red) mg/ml. (**c**) Fit of the $s_{20,w}$ isotherm (in the model of a monomer-dimer equilibrium), providing a dissociation constant Kd = 37 µM. The confidence interval for Kd is 31 - 46 µM considering a 68% confidence level. (**d**) Superposition of the mean $s_{20,w}$ for StkP^{287}-N*N (black), StkP^{287}-N*C (magenta), StkP^{287}-N*C/C*C/N*N (empty), and StkP287-C*C (yellow). $s_{20,w}$ were calculated using tabulated density and viscosity.

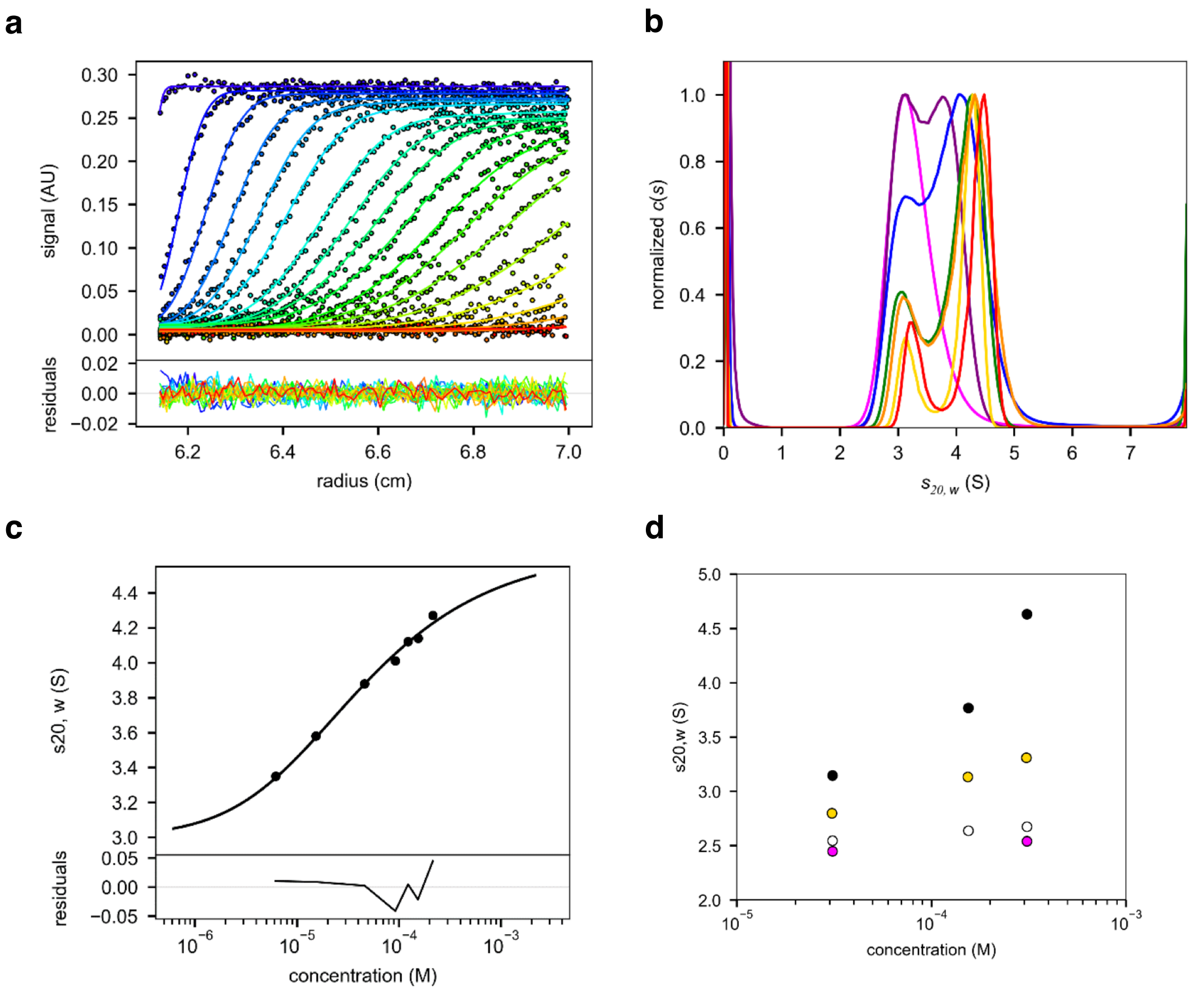
a
b
c
d
signal (AU)
residuals
radius (cm)
normalized c(s)
s20, w (S)
s20, w (S)
residuals
concentration (M)
s20,w (S)
concentration (M)

Supplementary Figure 9. Growth and localization of StkP dimerization mutants.

(**a**) Growth of *WT*, *stkP-C*C*, *stkP-N*C, stkP-N*N,* and *stkP-C*C/N*C/N*N* strains. Strains were grown in C + Y medium at pH 6,8 and at 37°C. (**b**) Expression of m*gfp-StkP*, m*gfp-stkP-N*C, mgfp-stkP-C*C,* and *mgfp-stkP-N*N*. The Western immunoblot was probed with anti-mGFP antibodies (α-GFP) (upper panel). (**c**) mGFP fluorescent signal (upper pannel), phase contrast (PC, middle pannel) and overlays (lower panel) of m*gfp-StkP, mgfp-stkP-N*C, mgfp-stkP-C*C,* and *mgfp-stkP-N*N* cells, respectively. Scale bar, 2 µm. 1000 cells were analyzed for each strain. All experiments were performed in triplicate.

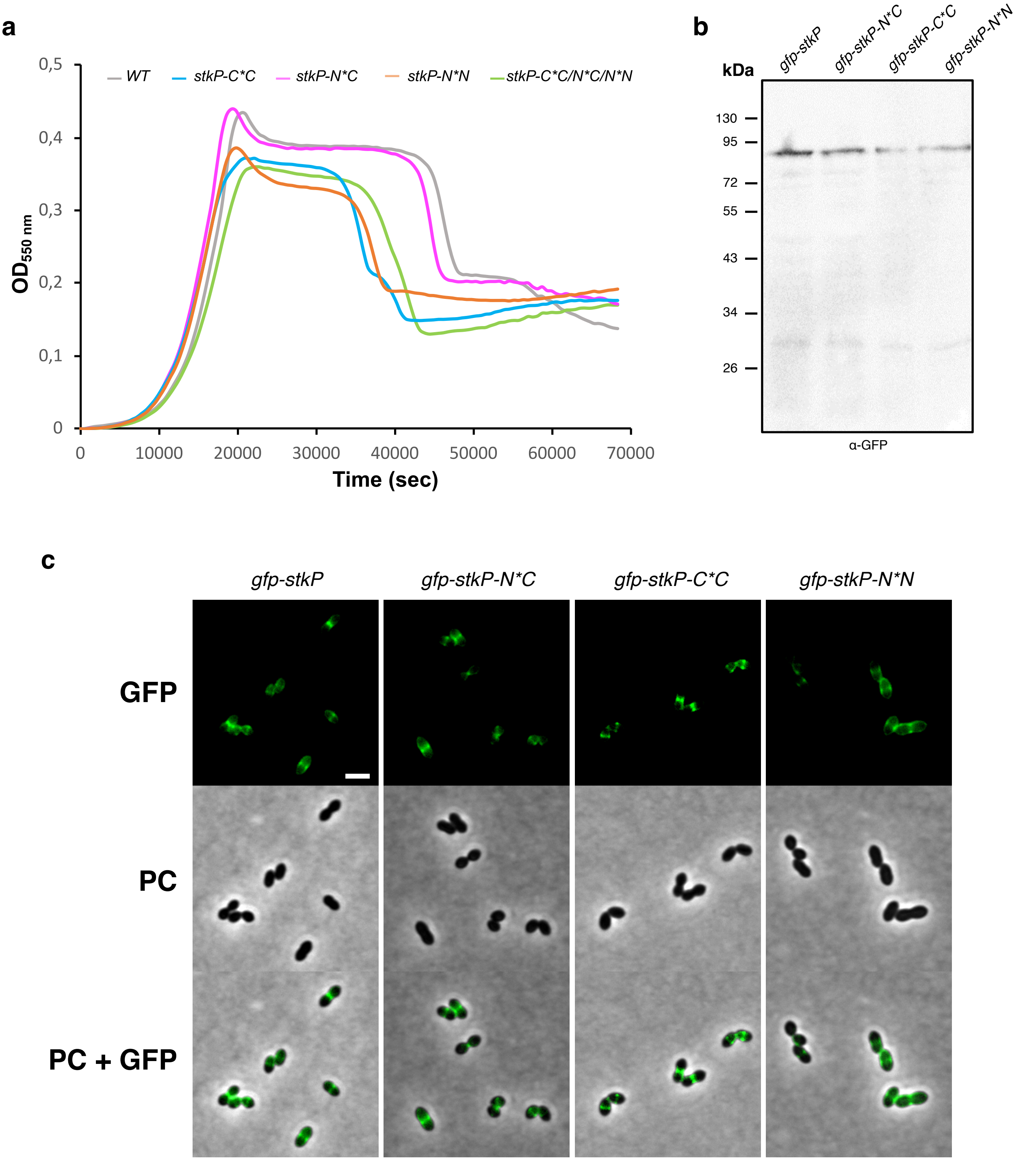

a
WT
stkP-C*C
stkP-N*C
stkP-N*N
stkP-C*C/N*C/N*N
0,5
0,4
0,3
0,2
0,1
0
OD550 nm
0
10000
20000
30000
40000
50000
60000
70000
Time (sec)
b
gfp-stkP
gfp-stkP-N*C
gfp-stkP-C*C
gfp-stkP-N*N
kDa
130
95
72
55
43
34
26
α-GFP
c
gfp-stkP
gfp-stkP-N*C
gfp-stkP-C*C
gfp-stkP-N*N
GFP
PC
PC + GFP

Supplementary Table 1. Data collection and refinement statistics.

	**$StkP_{344}$-2TA**	**$StkP_{287}$-2TA (Mg)**	**$StkP_{287}$-2TA (Mn)**	**$StkP_{287}$-2TE**
**Beamline**	ESRF ID29	SOLEIL PX1	ESRF ID23-1	ESRF ID23-1
**Wavelength**	1.07227	0.97856	0.88560	0.88560
**Resolution range**	46.39-2.8 (3.02-2.8)	46.05-1.794 (1.84-1.8)	59.208-1.883 (1.916-1.883)	67.22-1.59 (1.62-1.6)
**Space group**	P 1 21 1	P 1 21 1	P 1 21 1	P 1 21 1
**Number of molecules in the a.u.**	2[1]	2[2]	2[3]	2[4]
**Unit cell (Å, °)**	69.26, 51.95 81.82, 90.00,103.41, 90.00	69.28, 51.73, 81.14, 90.00, 103.77, 90.00	69.53, 51.79, 82.54, 90.00, 104.21, 90.00	69.43, 51.71, 82.19, 90.00, 104.49, 90.00
**Total reflections**	28113 (5591)	364468 (25259)	101061 (5173)	209989 (10628)
**Unique reflections**	14193 (2823)	52450 (3783)	45024 (2266)	74168 (3731)
**Multiplicity**	2.0 (2.0)	6.9 (6.7)	2.2 (2.3)	2.8 (2.8)
**Completeness (%)**	99.8 (99.9)	99.6 (97.3)	97.0 (98.4)	97.7 (97.9)
**Mean I/sigma(I)**	6.7 (1.4)	7.6 (0.8)	6.1 (0.8)	9.3 (1.2)
**Wilson B-factor**	43.83	29.97	26.09	22.45
**R-merge**	0.110 (0.740)	0.148 (1.683)	0.081 (0.988)	0.048 (0.633)
**R-meas**	1.155 (1.046)	0.174 (1.993)	0.105 (1.281)	0.068 (0.890)
**R-pim**	0.110 (0.740)	0.092 (1.060)	0.065 (0.806)	0.048 (0.625)
**CC1/2**	0.985 (0.459)	0.996 (0.503)	0.994 (0.350)	0.997 (0.517)
**Reflections used in refinement**	14189 (2823)	52425 (2670)	44867 (2711)	72910 (5264)
**Reflections used for R-free**	709 (149)	2621 (133)	2280 (127)	72910 (5264)
**R-work**	0.2237 (0.2914)	0.1905 (0.3148)	0.2110 (0.3188)	0.2081 (0.3476)
**R-free**	0.3026 (0.3804)	0.2382 (0.3207)	0.2518 (0.3698)	0.2334 (0.3698)
**Number of non-hydrogen atoms**	4089	4706	4507	4516
**macromolecules**	4026	4249	4272	4218
**ligands**	0	78	64	32
**solvent**	63	379	171	266
**Protein residues**	515	542	545	533
**RMS (bonds)**	0.009	0.015	0.007	0.007
**RMS (angles)**	1.22	1.46	1.02	0.88
**Ramachandran favored (%)**	86.88	95.51	94.41	97.5
**Ramachandran allowed (%)**	9.74	4.12	4.66	2.5
**Ramachandran outliers (%)**	3.38	0.37	0.93	0
**Rotamer outliers (%)**	8.64	1.55	0.88	0.44
**Clashscore**	17.21	4.22	3.67	2.58
**Average B-factor**	46.49	36.35	35.10	33.72
**macromolecules**	46.59	36.15	35.27	33.87
**ligands**		37.68	38.71	25.68
**solvent**	40.62	38.32	29.29	32.43

Statistics for the highest-resolution shell are shown in parentheses.

[1]The final model contains residues 4-155 and 174–271 in chain A and residues 4–46, 50-156 and 176–274 in chain B.

[2]The final model contains residues in chain A and residues in in chain B

[3]The final model contains residues 3–43, 55-158 and 176–285 in chain A, residues 2-287 in chain B.

[4]The final model contains residues 3-19, 24-44, 51-155, 176-285 in chain A and residues 2-165 and 172-287 in chain B.

Supplementary Table 2. SAXS data analysis performed at various concentrations of StkP^{287}-WT

(*a*) Sample details						
	StkP^{287}-WT					
Extinction coefficient [A_{280}, 0.1%(*w*/*v*)]	0.650					
Partial specific volume $\bar{\upsilon}$ ($cm^3 g^{-1}$)	0.731					
M from chemical composition (Da)	32084					
Concentration ($mg.mL^{-1}$)	1.3	2.6	5.2	9.1	10	13
Solvent composition	25 mM Bis-Tris pH 7.4, 150 mM NaCl					
(*b*) SAXS data collection parameters						
Source, instrument	BM 29 BIOSAXS ESRF					
Detector	Pilatus3 2M					
Energy (keV)	12.5					
Beam size (µm)	200x100					
q-measurement range (nm^{-1})	0.025 - 6 nm^{-1}					
Exposure time, number of exposures	1s, 10 exposure frames					
Sample loading	Automated sample changer					
Sample temperature (ºC)	4°C					
(*c*) Software employed for SAXS data reduction, analysis and interpretation						
SAXS data reduction	EDNA pipeline on BM29[1]					
Basic analyses: Guinier, *P*(*r*), V_P	ScÅtter IV[2]					
(*d*) Structural parameters						
Guinier Analysis – sample C	1.3	2.6	5.2	9.1	10	13
I(0) (cm^{-1})	31.03 ± 0.07	81.28 ± 0.18	221.36 ± 0.18	455.78 ± 0.84	518.34 ± 0.47	713.23 ± 0.70
R_g (Å)	22.73 ± 0.26	28.92 ± 0.49	28.16 ± 0.16	30.10 ± 0.31	30.63 ± 0.18	32.01 ± 0.20
q-range ($Å^{-1}$)	0.0106–0.3251	0.0126 – 0.2948	0.0095 – 0.3663	0.012–0.2993	0.0095 – 0.35600	0.010–0.3046
P(*r*) analysis – sample C						
I(0) (cm^{-1})	27.07 ± 0.00	78.01 ± 0.00	213.40 ± 0.00	447.50 ± 0.00	498.70 ± 0.00	688.30 ± 0.00
R_g (Å)	21.88 ± 0.00	28.20 ± 0.00	27.77 ± 0.00	30.53 ± 0.00	30.40 ± 0.00	32.04 ± 0.00
d_{max} (Å)	68.0	102.0	105.0	118.0	120.0	122.0

Supplementary Table 3. List of primers used in this study.

#	Primer Name	+/-	Sequence 5'→3'
1	5'- [kan-rpsL]	+	CCGTTTGATTTTTAATGGATAATG
2	3' - [kan-rpsL]	-	AGAGACCTGGGCCCCTTTCC
3	upstream region StkP 538	+	GGTCAGGTTACTGCTCTGGAC
4	downstream region StkP 541	-	GAAAGGAGCACTAAAGGTCGC
5	StkP-2TA	+	GCCTTTGCAGAGACAAGTCTGgCCCAGgCTAACTCGATGTTGGGCTCAGTTC
6	StkP-2TA	-	GAACTGAGCCCAACATCGAGTTAGcCTGGGcCAGACTTGTCTCTGCAAAGGC
7	linker mGFP-StkP	+	CTCGAGGGATCCGGAATGATCCAAATCGGCAAGATTTTTG
8	downstream mGFP-StkP	-	CATGGCATAGATATCACTCTGC
9	StkP-2TE	+	GCCTTTGCAGAGACAAGTCTGgAACAGgAAAACTCGATGTTGGGCTCAGTTC
10	StkP-2TE	-	GAACTGAGCCCAACATCGAGTTTTcCTGTTcCAGACTTGTCTCTGCAAAGGC
11	StkP-D136N	+	GGAATTGTTCACAGGAACTTGAAACCTCAAAATATCCTTTTG
12	StkP-D136N	-	CAAAAGGATATTTTGAGGTTTCAAGTTCCTGTGAACAATTCC
13	upstream region StkP 2371	+	GTTGATACCCAGATCGATACAG
14	downstream region StkP 2372	-	AGGCAAAACCAAATAAGGTCG
15	linker mGFP-StkP	-	ctcgagggatccggaatgatccaaatcggcaagatttttg
16	StkP-D154A	+	GATGGGACTGCCAAGGTCACAGCCTTTGGGATTGCTGTAG
17	StkP-D154A	-	CTACAGCAATCCCAAAGGCTGTGACCTTGGCAGTCCCATC
18	StkP-E61A	-	GATCTGCCATAGCTCTCGCTGCACGCTGAAAACGAGCTAC
19	StkP-E61A	+	GTAGCTCGTTTTCAGCGTGCAGCGAGAGCTATGGCAGATC
20	StkP-G174P	+	AACTCGATGTTGCCCTCAGTTCATTACTTGTCACCAG
21	StkP-G174P	-	CTGGTGACAAGTAATGAACTGAGGGCAACATCGAGTT
22	StkP-N*C	+	GCTCGGTTTCAGAGATGGCTGTAGACTTGTCTAGTAGCTTG
23	StkP-N*C	-	CAAGCTACTAGACAAGTCTACAGCCATCTCTGAAACCGAGC
24	StkP-N*N	+	CCAAAGACTTAATCGATGATGGGGAAGAAGTGGCAGTG
25	StkP-N*N	-	CACTGCCACTTCTTCCCCATCATCGATTAAGTCTTTGG
26	StkP-C*C	+	CGGGGATAGCGCGGATACCATTGAACTCCAGCATTTCCAG
27	StkP-C*C	-	CTGGAAATGCTGGAGTTCAATGGTATCCGCGCTATCCCCG
28	StkP-N*C/N*N/C*C	+	CGTTCGGATAACAGCTATTGGTGAGG
29	StkP-N*C/N*N/C*C	-	CCTCACCAATAGCTGTTATCCGAACG
30	StkP- pETPhos	+	GTATTTCCAGGGCCATATGATCCAAATCGGCAAGATTTTTG
31	StkP-E287-pETPhos	-	CTTAGTTATTAGGATCCTTCATCAAAGATTAACTTACTTTC
32	pETPhos	-	CATATGGCCCTGGAAATACAAG
33	pETPhos	+	GGATCCTAATAACTAAGTAAACTAGTGC
34	StkP-R344-pETPhos	-	CTTAGTTATTAGGATCCACGCATCTTAAATCTATGTTTTTTCGGTGC
35	StkP-D69A	+	GCGAGAGCTATGGCAGATCTAGCCCATCCTCATATCGTTCG
36	StkP-D69A	-	CGAACGATATGAGGATGGGCTAGATCTGCCATAGCTCTCGC
37	StkP-D78A	+	CGTTCGGATAACAGCTATTGGTGAGG
38	StkP-D78A	-	CCTCACCAATAGCTGTTATCCGAACG

Supplementary Table 4. Strains and plasmids used in this study.

Strain	Genotype and description	Reference	Primers (Table S2)
S. pneumoniae strains			
R800	*R800 rpsL1; Str^R*	Gift from J.-P. Claverys (France)	
Wild type	*R800 rpsL1; Str^R*	(3)	
stkP::kan-rpsL	*R800 rpsL1, stkP::kan-rpsL; Kan^R*	(3)	
ΔstkP	*R800 rpsL1, stkP-ΔstkP; Str^R*	(3)	
stkP-K42M	*R800 rpsL1, stkP-K42M; Str^R*	(4)	
mGFP-stkP	*R800 rpsL1, mGFP-stkP; Str^R*	(3)	
stkP-2TA (T167A, T169A)	*R800 rpsL1, stkP-2TA (T167A, T169A); Str^R*	This study	5, 6, 13 and 14 *(Wild type used as template)*
mGFP-stkP-2TA	*R800 rpsL1, mGFP-stkP-2TA; Str^R*	This study	5, 6, 13 and 14 *(mGFP-stkP used as template)*
stkP-2TE (T167E, T169E)	*R800 rpsL1, stkP-2TE (T167E, T169E); Str^R*	This study	9, 10, 13 and 14 *(Wild type used as template)*
mGFP-stkP-2TE	*R800 rpsL1, mGFP-stkP-2TE; Str^R*	This study	9, 10, 13 and 14 *(mGFP-stkP used as template)*
stkP-D136N	*R800 rpsL1, stkP-D136N; Str^R*	This study	11, 12, 13 and 14 *(Wild type used as template)*
stkP-D154A	*R800 rpsL1, stkP-D154A; Str^R*	This study	3, 4, 16 and 17 *(Wild type used as template)*
stkP-E61A	*R800 rpsL1, stkP-E61A; Str^R*	This study	3, 4, 18 and 19 *(Wild type used as template)*
stkP-G174P	*R800 rpsL1, stkP-G174P; Str^R*	This study	3, 4, 20 and 21 *(Wild type used as template)*
stkP-D69A	*R800 rpsL1, stkP-D69A; Str^R*	This study	35, 36, 13 and 14 *(Wild type used as template)*
*stkP-N*C (D69A, Y266A)*	*R800 rpsL1, stkP-N*C (D69A, Y266A); Str^R*	This study	22, 23, 13 and 14 *(stkP-D69A used as template)*
*stkP-C*C (V218D, A221E)*	*R800 rpsL1, stkP-C*C (V218D, A221E); Str^R*	This study	26, 27, 13 and 14 *(Wild type used as template)*
stkP-D78A	*R800 rpsL1, stkP-D78A; Str^R*	This study	24, 25, 13 and 14 *(Wild type used as template)*
*stkP-N*N (D78A, L34D)*	*R800 rpsL1, stkP-N*N (D78A, L34D); Str^R*	This study	24, 25, 13 and 14 *(stkP-D78A used as template)*
*stkP-2TE/N*C/N*N/C*C*	*R800 rpsL1, stkP-C*C/N*C/N*N; Str^R*	This study	35, 36, 13 and 14 *(stkP-N*N used as template)*
*stkP-C*C/N*C*	*R800 rpsL1, stkP-C*C/N*C/N*N; Str^R*	This study	26, 27, 13 and 14 *(stkP-N*C used as template)*

*stkP-C*C/N*C/N*N*	*R800 rpsL1, stkP-C*C/N*C/N*N; Str^R*	This study	28, 29, 13 and 14 *(stkP-2TE/N*C/N*N/C*C and stkP-C*C/N*C used as template)*
*mGFP-stkP-N*C*	*R800 rpsL1, mGFP-stkP-N*C; Str^R*	This study	7, 15, 13 and 14 *(stkP-N*C used as template)*
*mGFP-stkP-C*C*	*R800 rpsL1, mGFP-stkP-C*C; Str^R*	This study	7, 15, 13 and 14 *(stkP-C*C used as template)*
*mGFP-stkP-N*N*	*R800 rpsL1, mGFP-stkP-N*N; Str^R*	This study	7, 15, 13 and 14 *(stkP-N*N used as template)*
E. coli strains			
DH5α	*F– φ80lacZΔ M15 Δ (lacZYA-argF) U169 recA1 endA1 hsdR17 (r_K– m_K+) PhoA supE44 λ- thi–1 gyrA96 relA1*	Lab collection	
BL21 star (DE3)	*F – ompT gal dcm lon $hsdS_B(r_B - m_B -)$ λ(DE3 [lacI lacUV5-T7p07 ind1 sam7 nin5]) $[malB^+]_{K-12}(\lambda^S)$*	Invitrogen	
Plasmids			
pETPhos-$StkP^{287}$	*pETPhos derivative, encoding StkP, from Met1 to Glu287, Amp^R*	(5)	30 and 31 *(Wild type used as template)*
pETPhos-$StkP^{344}$-2TA	*pETPhos derivative, encoding StkP-2TA (T167A, T169A) from Met1 to Arg344, Amp^R*	This study	30 and 34 *(stkP-2TA used as template)*
pETPhos-$StkP^{287}$-2TA	*pETPhos derivative, encoding StkP-2TA (T167A, T169A) from Met1 to Glu287, Amp^R*	This study	30 and 31 *(stkP-2TA used as template)*
pETPhos-$StkP^{287}$-2TE	*pETPhos derivative, encoding StkP-2TE (T167E, T169E) from Met1 to Glu287, Amp^R*	This study	30 and 31 *(stkP-2TE used as template)*
*pETPhos-$StkP^{287}$-N*C*	*pETPhos derivative, encoding StkP- N*C (D69A, Y266A) from Met1 to Glu287, Amp^R*	This study	30 and 31 *(stkP-N*C used as template)*
*pETPhos-$StkP^{287}$-C*C*	*pETPhos derivative, encoding StkP- C*C (V218D, A221E), from Met1 to Glu287, Amp^R*	This study	30 and 31 *(stkP-C*C used as template)*
*pETPhos-$StkP^{287}$-N*N*	*pETPhos derivative, encoding StkP- N*C (D78A, L34D) from Met1 to Glu287, Amp^R*	This study	30 and 31 *(stkP-N*N used as template)*

Supplementary Table 5. Setup for the molecular dynamics simulations.

In crystallo	**(StkP^{344}-2TA dimer: 2AMP-PNP:2Mg^{2+})x2 (A,B,C,D)**	**(StkP^{344}-2TA dimer: 2ATP:2Mg^{2+})x2 (A,B,C,D)**
Starting structure	X-ray + RCD+	X-ray + RCD+
Box dimensions (Å^3)	138 x 122 x 147	138 x 122 x 147
Minimum distance (Å) between any solute atom and the edge of the periodic box	12	12
Water molecules	65 408	65 409
Ions	19 Cl$^-$ 30 K$^+$	18 Cl$^-$ 30 K$^+$
Total number of atoms	215 911	215 909
Trajectory length (300 K)	300 ns	300 ns
Trajectory length (300$\rightarrow$273K)	1 ns x 60	1 ns x 60

**In solution**	**wtStkP^{287}:ATP:2Mg^{2+}/ wtStkP^{287}:ADP:Mg^{2+}**	***wt*StkP:ATP:2Mg^{2+} (replica 1)**
Starting structure	X-ray + RCD+	X-ray + RCD+
Box dimensions (Å^3)	112 x 93 x 94	91 x 72 x 76
Minimum distance (Å) between any solute atom and the edge of the periodic box	12	12
Water molecules	24 482	12 177
Ions	5 Na$^+$	28 Cl$^-$ 30 K$^+$
Total number of atoms	82 362	40 963
Trajectory length (300 K)	300 ns	300 ns
Trajectory length (300$\rightarrow$273K)	1 ns x 60	1 ns x 60
	***wt*StkP:ATP:2Mg^{2+} (replica 2)**	***wt*StkP:ATP:2Mg^{2+} (replica 3)**
Starting structure	X-ray + RCD+	X-ray + RCD+
Box dimensions (Å^3)	91 x 72 x 76	91 x 72 x 76
Minimum distance (Å) between any solute atom and the edge of the periodic box	12	12
Water molecules	12 073	12 177
Ions	28 Cl$^-$ 30 K$^+$	28 Cl$^-$ 30 K$^+$
Total number of atoms	40 837	40 963
Trajectory length (300 K)	300 ns	300 ns
Trajectory length (300$\rightarrow$273K)	1 ns x 60	1 ns x 60
	**StkP.2TA:ATP:2Mg^{2+} (replica 1)**	**StkP.2TA:ATP:2Mg^{2+} (replica 2)**
Starting structure	X-ray + RCD+	X-ray + RCD+
Box dimensions (Å^3)	91 x 73 x 76	91 x 72 x 76
Minimum distance (Å) between any solute atom and the edge of the periodic box	12	12
Water molecules	12 219	12 076
Ions	28 Cl$^-$ 30 K$^+$	28 Cl$^-$ 30 K$^+$
Total number of atoms	41 267	48 838
Trajectory length (300 K)	300 ns	300 ns
Trajectory length (300$\rightarrow$273K)	1 ns x 60	1 ns x 60

**In solution**	**StkP.2TA:ATP:2Mg^{2+} (replica 3)**	**StkP.2TE:ATP:2Mg^{2+} (replica 1)**
Starting structure	X-ray + RCD+	X-ray + RCD+
Box dimensions (Å^3)	91 x 72 x 82	91 x 72 x 76
Minimum distance (Å) between any solute atom and the edge of the periodic box	12	12
Water molecules	13 118	12 080
Ions	28 Cl^- 30 K^+	26 Cl^- 30 K^+
Total number of atoms	43 964	40 858
Trajectory length (300 K)	300 ns	300 ns
Trajectory length (300→273K)	1 ns x 60	1 ns x 60
	**StkP.2TE:ATP:2Mg^{2+} (replica 2)**	**StkP.2TE:ATP:2Mg^{2+} (replica 3)**
Starting structure	X-ray + RCD+	X-ray + RCD+
Box dimensions (Å^3)	91 x 72 x 76	91 x 72 x 82
Minimum distance (Å) between any solute atom and the edge of the periodic box	12	12
Water molecules	12 073	13 116
Ions	26 Cl^- 30 K^+	26 Cl^- 30 K^+
Total number of atoms	40 837	43 966
Trajectory length (300 K)	300 ns	300 ns
Trajectory length (300→273K)	1 ns x 60	1 ns x 60
	**StkP.2TPO:ATP:2Mg^{2+}**	**StkP.2TPO:ATP:2Mg^{2+}**
Starting structure	SwissModel (template = Xray)	SwissModel (template = 4HPU)
Box dimensions (Å^3)	91 x 72 x 82	92 x 77 x 76
Minimum distance (Å) between any solute atom and the edge of the periodic box	12	12
Water molecules	13 114	12 954
Ions	28 Cl^- 30 K^+	28 Cl^- 30 K^+
Total number of atoms	43 960	43 482
Trajectory length (300 K)	300 ns	300 ns
Trajectory length (300→273K)	1 ns x 60	1 ns x 60
	**$StkP^{287}$-TPO^{167}:ATP:2Mg^{2+}**	**$StkP^{287}$-TPO^{169}:ATP:2Mg^{2+}**
Starting structure	AlphaFold 3 + PTM	AlphaFold 3 + PTM
Box dimensions (Å^3)	94 x 74 x 80	94 x 74 x 78
Minimum distance (Å) between any solute atom and the edge of the periodic box	12	12
Water molecules	13 527	13 305
Ions	25 Cl^- 30 K^+	25 Cl^- 30 K^+
Total number of atoms	45 198	44 532
Trajectory length (300 K)	300 ns	300 ns
Trajectory length (300→273K)	1 ns x 60	1 ns x 60

In solution	StkP^{287}-2TPO:ATP:2Mg^{2+}	StkP^{280}-2TPO:ATP:2Mg^{2+} / wtStkP^{287} dimer
Starting structure	AlphaFold 3 + PTM	AlphaFold 3 + PTM
Box dimensions (Å^3)	94 x 74 x 89	96 x 113 x 96
Minimum distance (Å) between any solute atom and the edge of the periodic box	12	12
Water molecules	13 526	27 068
Ions	23 Cl^- 30 K^+	8 Na^+
Total number of atoms	45 196	90 089
Trajectory length (300 K)	300 ns	300 ns
Trajectory length (300→273K)	1 ns x 60	1 ns x 60

Supplementary References

1. Pernot P, Round A, Barrett R, De Maria Antolinos A, Gobbo A, Gordon E, et al. Upgraded ESRF BM29 beamline for SAXS on macromolecules in solution. J Synchrotron Radiat. 2013; 20: 660–664.
2. Tully MD, Tarbouriech N, Rambo RP & Hutin S. Analysis of SEC-SAXS data via EFA deconvolution and Scatter. J Vis Exp. 2021; doi:10.3791/61578.
3. Fleurie A, Cluzel C, Guiral S, Freton C, Galisson F, Zanella-Cleon I, et al. Mutational dissection of the S/T-kinase StkP reveals crucial roles in cell division of Streptococcus pneumoniae. Mol Microbiol. 2012; 83:746-58.
4. Fleurie A, Manuse S, Zhao C, Campo N, Cluzel C, Lavergne JP, et al. Interplay of the serine/threonine-kinase StkP and the paralogs DivIVA and GpsB in pneumococcal cell elongation and division. PLoS Genet. 2014;10: e1004275.
5. Hamidi M, Nagarajan SN, Ravikumar V, Gueguen-Chaignon V, Laguri C, Freton C, et al. The juxtamembrane domain of StkP is phosphorylated and influences cell division in Streptococcus pneumoniae. mBio 2025; 16: e0379924

Supplementary Fig. 2a

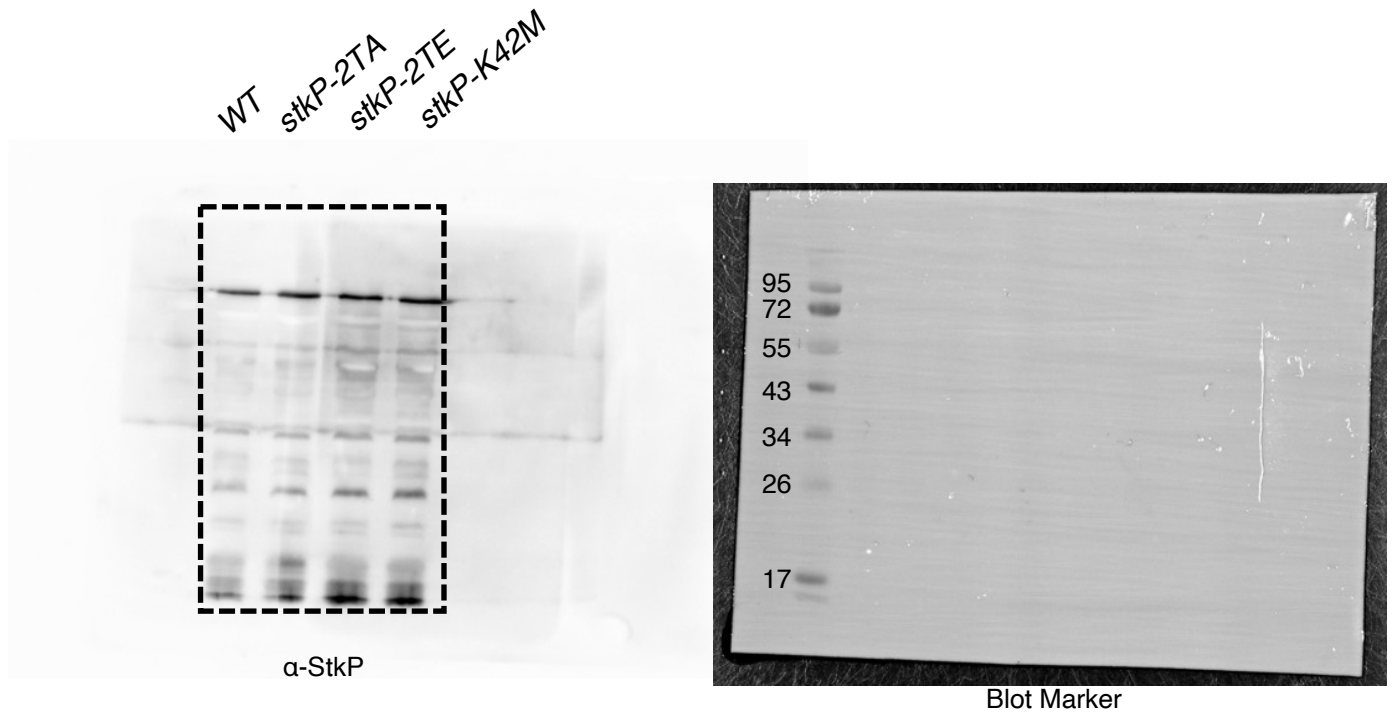

Supplementary Fig. 2c

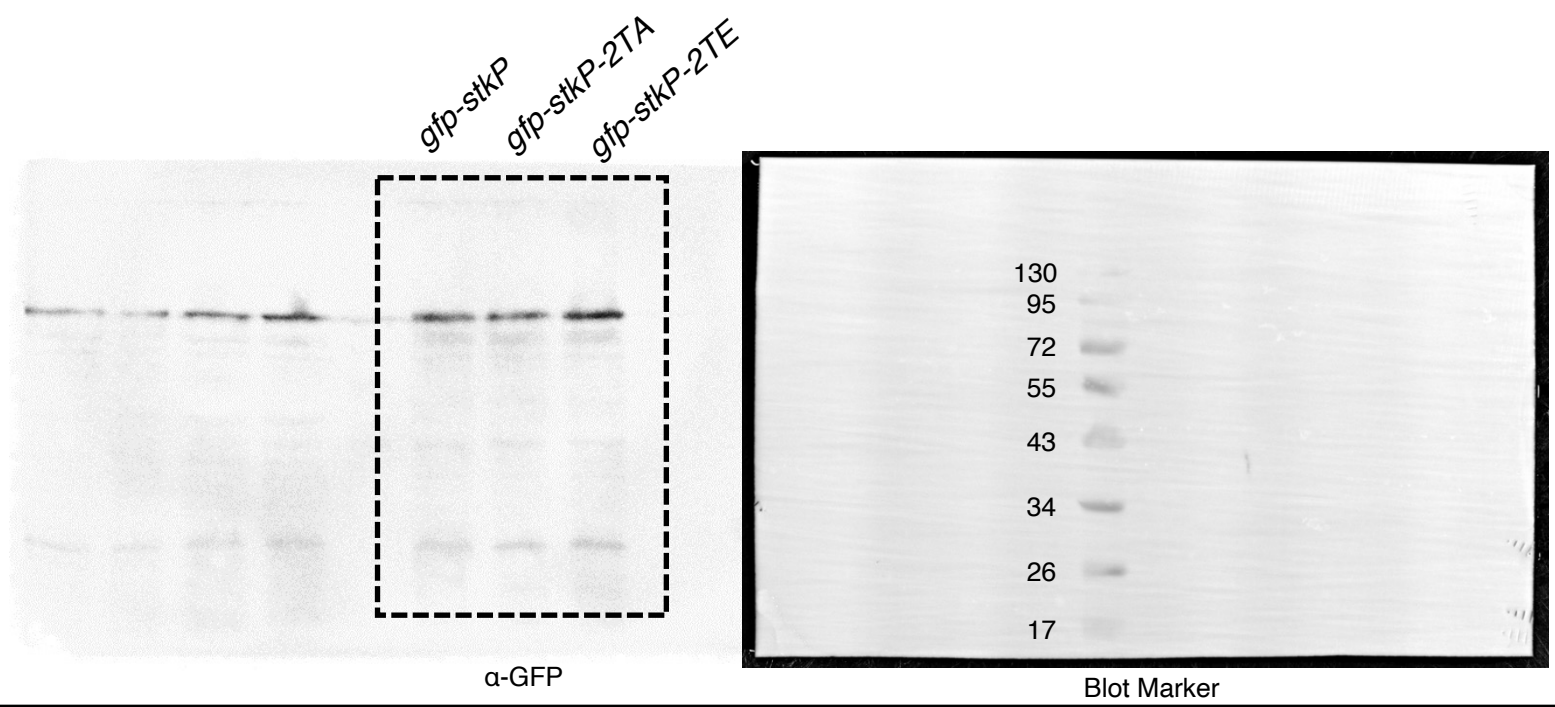

Supplementary Fig. 3a

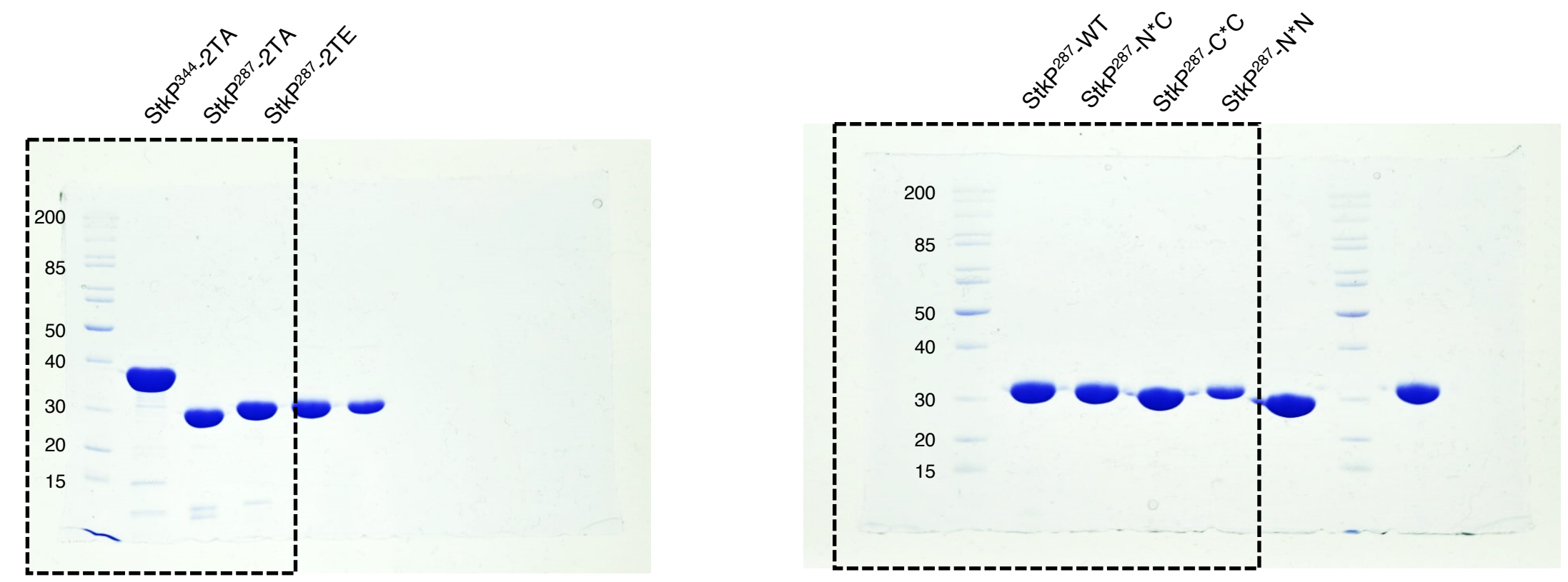

Supplementary Fig. 4b

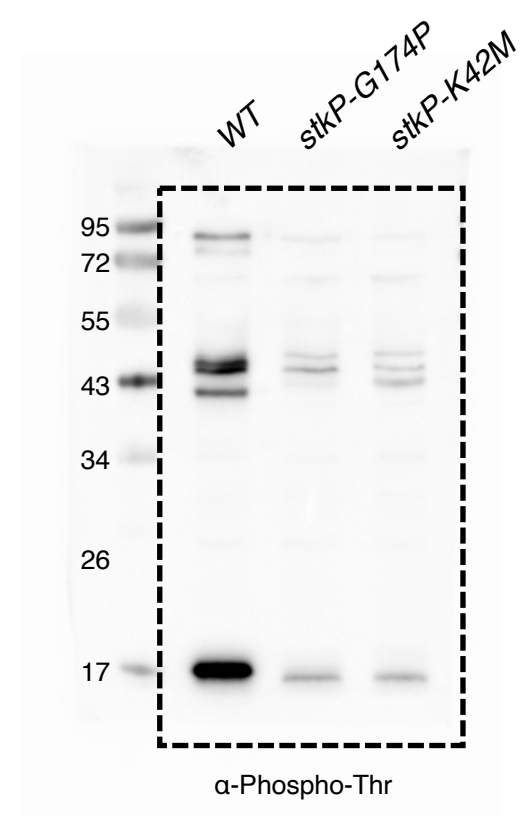

Supplementary Fig. 9b

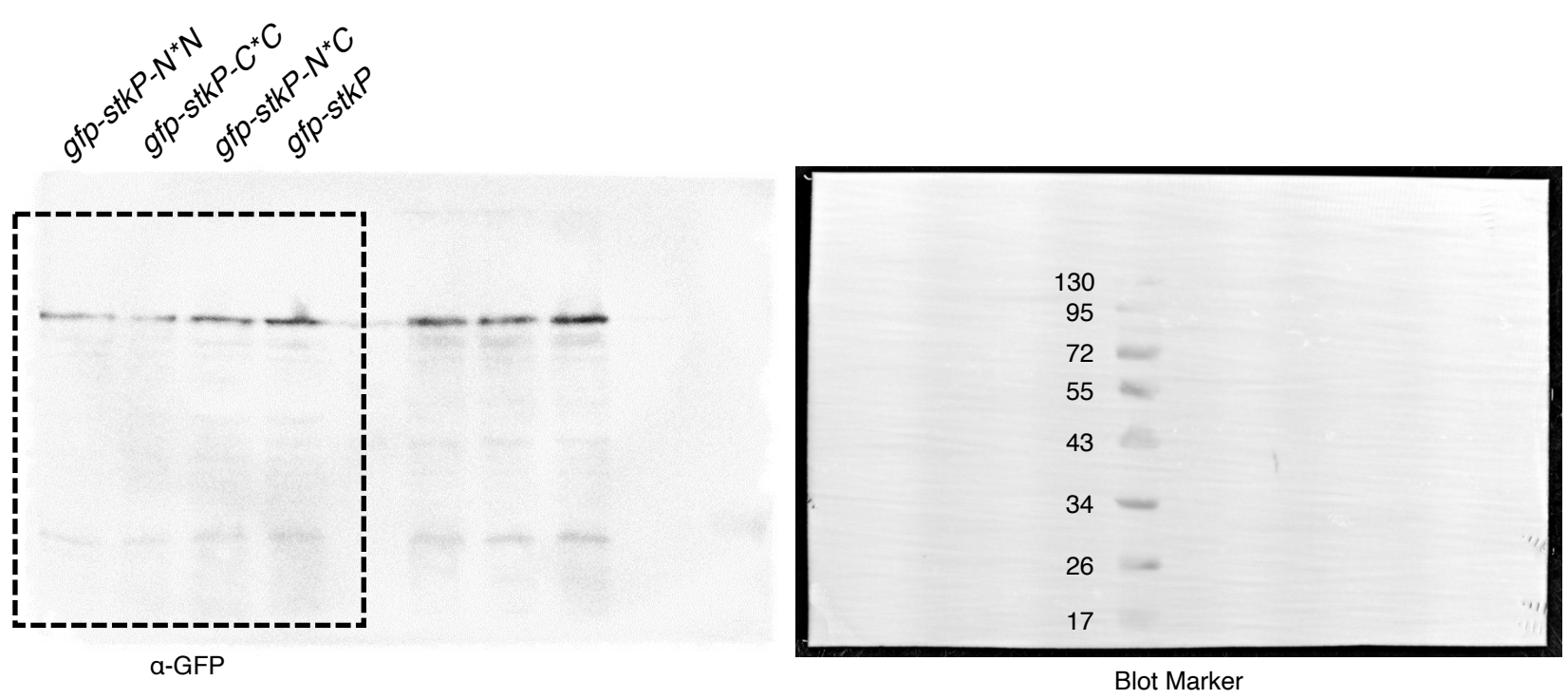

Supplement: Supplementary file 1 [file 41467_2026_74470_MOESM1_ESM.pdf]
