# Supplementary material for: Structural dissection of the catalytic domain of the serine threonine kinase StkP of Streptococcus pneumoniae
Source: Nat Commun. 2026 Jun 18;17:7687. doi: 10.1038/s41467-026-74470-6 (PMC13434768; doi:10.1038/s41467-026-74470-6)
Supplement: Supplementary file 2 [file 41467_2026_74470_MOESM2_ESM.pdf]

The Description of Additional Supplementary Files

Supplementary Video 1: Molecular dynamics simulation of StkP287- 2TA with Mg2+-bound ATP.

Supplementary Video 2: Molecular dynamics simulation of WT StkP287 with Mg2+-bound ATP.

Supplementary Video 3: Molecular dynamics simulation of phosphorylated StkP287- 2pT with Mg2+-bound ATP.

Supplementary Video 4: Molecular dynamics simulation of StkP287- 2TE with Mg2+-bound ATP.

Supplementary Video 5: Molecular dynamics simulation of StkP287- 2pT with Mg2+-bound ATP zoomed-in on the αC helix and the formation the catalytically essential K42–E61 salt bridge.
