# Supplementary material for: Structural dissection of the catalytic domain of the serine threonine kinase StkP of Streptococcus pneumoniae
Source: Nat Commun. 2026 Jun 18;17:7687. doi: 10.1038/s41467-026-74470-6 (PMC13434768; doi:10.1038/s41467-026-74470-6)
Supplement: Supplementary file 4 [file 41467_2026_74470_MOESM9_ESM.pdf]

nature portfolio

Peer Review File

Structural dissection of the catalytic domain of the serine threonine kinase StkP of Streptococcus pneumoniae

Corresponding Author: Dr Christophe Grangeasse

This file contains all reviewer reports in order by version, followed by all author rebuttals in order by version.

Version 0:

Reviewer comments:

Reviewer #1

(Remarks to the Author)
The authors of this paper report on the structural basis for the activation of the serine/threonine protein kinase StKP from Streptococcus pneumoniae. Using a combination of X-ray crystallography, mutational analysis, SAXS, and cell imaging, the authors propose a model of kinase activation regulated by local kinase concentration. Specifically, the authors propose an inactive dimer to an active conformational shift induced by the local concentration of the kinase domain. This conformational change exposes the activation loop in the kinase domain to endogenous substrates for phosphorylation. Given that StKP can be a potential target for S. pneumoniae, the proposed studies can inform drug discovery efforts. The integration of multiple approaches to investigate StKP activation is also a strength of the study. The integrative approaches employed, including molecular dynamics simulation, to probe StKP structural dynamics, will provide a framework for investigating the bacterial kinome, which remains relatively understudied compared to human and other eukaryotic kinases. My minor suggestions for the authors are below:

1. It would be good to include some discussions on how the mechanistic details obtained on StKP shed light on other bacterial serine-threonine kinases with an N-terminal PASTA domain. Also, there appear to be some similarities in human receptor tyrosine kinase regulation and StKP regulation that may be worth exploring/speculating on in the discussion.
2. Molecular dynamics simulations and structural comparisons show conformational changes in the G-loop and the G-helix, but these structural changes are not adequately described on incorporated into the structural basis of StKP activation.

Reviewer #2

(Remarks to the Author)
In the manuscript entitled “Structural dissection of the catalytic domain of the serine/threonine kinase StkP of Streptococcus pneumoniae” the authors report crystal structures of PASTA-Hanks kinase StkP variants, demonstrating that phosphorylation of two threonine residues regulates the structure and dynamics of the activation loop. Their findings also reveal an alternative mode of dimerization, whereby inactive dimers form at the bacterial division site, preventing substrate binding. By integrating structural, computational, biophysical, and cellular imaging studies, the authors propose a mechanism in which reorganization into active dimers enables substrate phosphorylation, providing new insights into the regulation of bacterial cell division by PASTA-Hanks kinases.

Overall, this work is of potential interest in the field, as the $StkP^{287}$-2TA structure represents the first bacterial Hanks kinase structure with a fully resolved activation loop. Furthermore, the study contributes to a clearer understanding of the in vivo activation mechanism of PASTA-Hanks kinases, a topic that remains highly debated.
However, some concerns remain regarding the use and presentation of molecular dynamics (MD) simulations, which, as noted by the authors, support the proposed link between loop mobility and phosphorylation. The reporting of the MD results is not sufficiently clear or detailed, and the simulations themselves could have been more fully leveraged. Given their suitability for probing the conformational landscape of protein kinases, MD simulations provide a particularly valuable means of investigating how phosphorylation affects the orientation of key structural elements, including the αC helix.
Major Points
1. In the Methods section, only the MD simulation of the $StkP^{287}$-2TA system in complex with AMP-PNP and Mg^{2+} is described, whereas $StkP^{287}$-WT, $StkP^{287}$-2pT, and $StkP^{287}$-2TE were also simulated. The modeling procedures for these

systems should be clarified in detail. It would be advisable to include a table listing, for each simulated system, the total number of atoms (protein, solvent, and ions), and the length of each trajectory.
2. The presentation of the MD results is limited. Only a single trajectory plot is provided (Fig. 4a), showing the RMSFs of the system referred to as "StkP^{287}-2T" in the figure legend. Were 300 ns sufficient to ensure convergence of the systems? How much did the simulated systems deviate from their initial states during the course of the trajectories? Furthermore, monitoring key interatomic distances over the simulations would strengthen the description of conformational motions.
3. The simulations were conducted over a relatively short timescale, limiting the robustness of the conclusions. For each system, at least three independent replicas should be performed, and the corresponding analyses reported.
4. The three highest-scoring conformations of the activation loop generated by the RCD+ server were used as independent replicas for the StkP^{287}-2TA simulation. However, it should be noted that the RCD+ algorithm is optimized for loop lengths of up to 12 residues. A comment on the rationale for using RCD+ in this context would be appropriate.
5. It is well established that AlphaFold3 performs well in modeling structured regions, but is less reliable for intrinsically disordered regions. Since the authors note that the degree of confidence is lower for the activation loop than for the rest of the protein, Extended Data Fig.4 should include the predicted local distance difference test (pLDDT) scores for the AlphaFold3 models. Moreover, the discussion regarding the orientation of the αC-helix and the K42–E61 salt bridge, based on the AlphaFold3 model, would be more robust if supported by a broader panel of MD simulations. Despite their computational cost, MD simulations have been widely applied to investigate conformational transitions in kinases.
6. Figure 4. The authors should clarify which representative MD snapshot is shown, and on what basis it was selected.
Minor Points
1. There are few inaccuracies in the legend of Extended Data Figure 4.
2. Correct ANP.
3. The x-axis label is missing in Fig. 4a.

Reviewer #3

(Remarks to the Author)
This study reports the structural analysis of the catalytic domain of the Hanks protein kinase StkP from S. pneumoniae and examines how the phosphorylation of the activation loop and the dimerization of the catalytic domain contribute to its activation. This is a solid work combining X-ray crystallography with biophysical methods, molecular dynamics simulations, in vitro and in vivo mutational analysis and cell imaging. The data support the two major conclusions of the manuscript: firstly, that the phosphorylation of the activation loop regulates activity by influencing the loop conformation and dynamics; and secondly, that kinase activation in vivo is driven by concentration-dependent dimerization of the catalytic domain, indicating that the binding of peptidoglycan fragments or precursors to PASTA domains is not essential for StkP activation. This study is based on well-designed experiments, and the results are of importance to the field of bacterial signaling. However, there are some points (detailed below) that must be addressed by the authors prior to publication.

The authors state in the introduction that the current model involves peptidoglycan-induced PASTA dimerization (as shown schematically in Fig.1a) acting as signaling domains to trigger kinase activation, and refer to this model as "a conventional ligand-induced activation paradigm of PASTA-Hanks kinases" (page 20, line 6). However, if extracellular ligand-induced dimerization could be considered a paradigm for eukaryotic receptor kinases, it is clearly not a paradigm for bacterial transmembrane kinases. Particularly in the case of PASTA kinases, ligand-induced dimerization is just a highly speculative hypothesis lacking experimental support that, to the best of my knowledge, was put forward by Barthe et al (Structure, 18, 606-615, 2010) based on a vague homology of PASTA kinases with the unrelated structure of PASTA-containing PBP2X (Pares et al, Nat Struct Biol, 3, 284-289, 1996). In contrast, recent studies from various laboratories, including the authors' own, have cast significant doubt on this interpretation and even questioned the function of PASTA subunits as signaling domains. The current knowledge about the possible roles of PASTA domains in StkP activity needs to be presented in a more balanced manner, and Fig. 1a should be modified accordingly.

The molecular dynamics simulations described in page 10 indicate that the activation loop was highly flexible in the 2pT and 2TE mutants, in agreement with the crystal structures, but remained in a conformation obstructing the active site in the wt and the 2TA mutant. As this is a central result of the manuscript, these mobility differences should be shown in Figure 4, possibly by modifying panel 4A.

The crystal structures revealed a novel, asymmetric dimerization mode of the catalytic domain, involving contacts between the N-lobe of one molecule and the C-lobe of the second molecule (the NC interface), which maintains the kinase in an inactive conformation. This NC interface differs from the back-to-back (NN) and front-to-front (CC) dimerization modes observed for M. tuberculosis PknB. Importantly, the AUC experiments (Figs. 5d-g) showed that the NC dimers seen in the crystals are also present in solution. This, together with the conservation of the dimeric interface in various Streptococcus species, suggest a putative physiological role of these NC StkP dimers. Such conservation is difficult to see in the multiple alignment (Extended Data Fig. 5) and should be better shown by mapping the conserved residues on the molecular surface of StkP.

The analysis of the phosphorylation pattern, growth and cell morphology of three StkP mutated pneumococcal strains, respectively unable to form NC, NN or CC dimers, provided important information on the biological relevance of the dimerization interfaces. The N*N and C*C mutants, but not the N*C mutant, exhibited significantly altered phosphorylation patterns and impaired growth, consistent with those of a dead-kinase mutant strain. These results are relevant, as they suggest that the CC and N-N dimers are important for kinase activation, in a similar way as proposed for PknB. However,

neither of these complexes could be detected by AUC in vitro, not even for the N*C mutant at the highest tested concentration of 10 mg/ml (Fig. 5e). While I can understand this for the CC complex (as it possibly corresponds to the transient autophosphorylation complex), it is more difficult to see why the N-N dimer could not be detected.

As the N*C mutant strain exhibit a normal phosphorylation pattern in vivo, the authors suggest that these dimers may play a non-catalytic role. They propose a revised model for StkP activation according to which NC dimerization represents a resting inactive state to prevent premature activation, and further interaction with a (so far unidentified) cellular partner may be required to trigger the transition into the active NN dimer. However, some difficulties with this model are that cell growth and morphologies of the N*C mutant strain are wild-type-like, suggesting that NC dimerization would not be required for normal cell division in vivo, and that the StkP NN dimers could not be detected in vitro, as noted above. Could it be possible that the mutations of the putative NN interface could affect kinase activity by a mechanism other than precluding NN dimerization?

In page 15, lines 1-3, the authors state that “the absence of an elongated phenotype for the C*C mutant strain suggests that mutating this interface suppresses the need of StkP kinase activity for cell division”, implying that kinase activity is normally required for cell division. However, the same chain-like morphological phenotype seen for the C*C strain was also observed for the ΔstpK mutant strain (Fig. 1e). As the C*C mutation is expected to prevent formation of the autophosphorylation complex, one could think from the above phenotypes that kinase activity would not be required for cell division. Could the authors comment on this?

In page 8, lines 24-26, the authors state that “stabilization of the activation loop resulted from the addition of AMP-PNP and cations, rather than crystal packing”. However, the inspection of the highest resolution structure suggests the opposite conclusion, namely that stabilization resulted primarily from crystal contacts. Indeed, the ordered loop in the first independent molecule is engaged in intermolecular contacts with two other StkP molecules in the crystal, whereas the same loop in the second independent molecule (which obviously binds the same nucleotide and cations) is less engaged in crystal contacts and is structurally disordered.

In Page 10, lines 22-26, the authors describe the G174P mutation, which they designed to investigate the flexibility of the activation loop. However, in the crystal structure Gly174 adopts a main-chain conformation that is not compatible with a proline residue, and the substitution is therefore introducing conformational changes. I wonder whether these changes, rather than the decreased mobility, could account for the observed growth effects (Fig. 4d). The authors should comment about this.

In Page 10, lines 7-8, the authors state that this is the first reported bacterial Hank-kinase structure in which the activation loop is fully resolved. This is not the case, as for instance the structure of M. tuberculosis PknG also has a well-defined activation loop in the crystal structures (Scherr et al, PNAS 104, 12151, 2007; Lisa et al, Structure 23, 1039, 2015).

In page 17, line 8, change ‘cognate phosphate’ for ‘cognate phosphatase’.

In page 17, lines 15-16: Figure 4 does not show the differences in loop mobility between the 2TA and 2TE mutants (see comment above on Figure 4).

The legend to Fig. 4C is missing.

Version 1:

Reviewer comments:

Reviewer #1

(Remarks to the Author)
The authors have satisfactorily addressed my comments and suggestions

Reviewer #2

(Remarks to the Author)
Dear Authors,
Thank you for your thorough and well-organized responses to the comments. I am satisfied that the concerns I raised have been carefully considered and addressed.

Reviewer #3

(Remarks to the Author)
I believe that the authors have addressed most of my concerns and those from the other reviewers in the revised version. The toned down interpretations make for a more balanced discussion, even if I’m still not fully convinced that, based on the crystallographic data alone, one could conclude that stabilization of the activation loop is primarily due to nucleotide and cation binding.

Open Access This Peer Review File is licensed under a Creative Commons Attribution 4.0 International License, which permits use, sharing, adaptation, distribution and reproduction in any medium or format, as long as you give appropriate credit to the original author(s) and the source, provide a link to the Creative Commons license, and indicate if changes were made.
In cases where reviewers are anonymous, credit should be given to 'Anonymous Referee' and the source.
The images or other third party material in this Peer Review File are included in the article's Creative Commons license, unless indicated otherwise in a credit line to the material. If material is not included in the article's Creative Commons license and your intended use is not permitted by statutory regulation or exceeds the permitted use, you will need to obtain permission directly from the copyright holder.
To view a copy of this license, visit https://creativecommons.org/licenses/by/4.0/

Reviewer #1 (Remarks to the Author):

The authors of this paper report on the structural basis for the activation of the serine/threonine protein kinase StKP from Streptococcus pneumoniae. Using a combination of X-ray crystallography, mutational analysis, SAXS, and cell imaging, the authors propose a model of kinase activation regulated by local kinase concentration. Specifically, the authors propose an inactive dimer to an active conformational shift induced by the local concentration of the kinase domain. This conformational change exposes the activation loop in the kinase domain to endogenous substrates for phosphorylation. Given that StKP can be a potential target for S. pneumoniae, the proposed studies can inform drug discovery efforts. The integration of multiple approaches to investigate StKP activation is also a strength of the study. The integrative approaches employed, including molecular dynamics simulation, to probe StKP structural dynamics, will provide a framework for investigating the bacterial kinome, which remains relatively understudied compared to human and other eukaryotic kinases. My minor suggestions for the authors are below:
We thank the Referee for the positive feedback on our manuscript. Below, we provide our responses to his/her comments.

1. It would be good to include some discussions on how the mechanistic details obtained on StKP shed light on other bacterial serine-threonine kinases with an N-terminal PASTA domain. Also, there appear to be some similarities in human receptor tyrosine kinase regulation and StKP regulation that may be worth exploring/speculating on in the discussion.
We agree. We have modified the discussion (page 20, line 21 to page 22 line 2) to mention that the mechanistic details reported for StkP may shed light on the understanding of the activation process of other bacterial PASTA-Hanks kinases. However, discussing the potential similarities with human and other eukaryotic kinases, which are devoid of PASTA domains and which highly differ in their activation mechanisms, may be to much speculative and we think it's better to stick to the comparison to bacterial PASTA-Hanks kinases.

2. Molecular dynamics simulations and structural comparisons show conformational changes in the G-loop and the G-helix, but these structural changes are not adequately described on incorporated into the structural basis of StKP activation.
The conformational changes observed in the G-loop and G-helix are subtle, as described in the Results section, and do not allow us to draw firm conclusions regarding their specific contribution to StkP activation. Given the limited magnitude of these differences, we have avoided overinterpreting their structural or functional significance. Nevertheless, we agree that the G-loop (Hanks motif I) and the G-helix display minor positional variability. Because they are located in close proximity to the activation loop, it suggests that they may exhibit dynamics that accompany activation loop movements. In agreemment with the referee's comment, and to provide clearer structural context, we modified the Extended Figure 3d highlighting the relative positions of the G-loop and G-helix with respect to the activation loop. We have also modified the text (page 9, line 2) to mention the conformational changes of the G-loop and G-helix.

Reviewer #2 (Remarks to the Author):

In the manuscript entitled "Structural dissection of the catalytic domain of the serine/threonine kinase StkP of Streptococcus pneumoniae" the authors report crystal structures of PASTA-Hanks kinase StkP variants, demonstrating that phosphorylation of two threonine residues regulates the structure and dynamics of the activation loop. Their findings also reveal an alternative mode of dimerization, whereby inactive dimers form at the bacterial division site, preventing substrate binding. By integrating structural, computational, biophysical, and cellular imaging studies, the authors propose a mechanism in which reorganization into active dimers enables substrate phosphorylation, providing new insights into the regulation of bacterial cell division by PASTA-Hanks kinases.
Overall, this work is of potential interest in the field, as the StkP^{287}-2TA structure represents the first bacterial Hanks kinase structure with a fully resolved activation loop. Furthermore, the study contributes to a clearer understanding of the in vivo activation mechanism of PASTA-Hanks kinases, a topic that remains highly debated. However, some concerns remain regarding the use and presentation of molecular dynamics (MD) simulations, which, as noted by the authors, support the proposed link between loop mobility and phosphorylation. The reporting of the MD results is not sufficiently clear or detailed, and the simulations themselves could have been more fully leveraged. Given their suitability for probing the conformational landscape of protein kinases, MD simulations provide a particularly valuable means of investigating how phosphorylation affects the orientation of key structural elements, including the αC helix.
We thank the referee for his/her positive comments and we provide below our answers to his/her comments together with new experiments that strengthen our conclusions.

We understand the reviewers' concerns and apologize for not making the computational section much clearer. It has been argued that for robust statistical analysis and a thorough exploration of a system's behavior, using multiple short simulations from different starting configurations is generally more effective and statistically reliable than running a few very long simulations because of the well-known risk of getting trapped in a local energy minimum, thus failing to explore the full conformational landscape (Knapp et al., *J. Chem. Theory Comput*. 2018, 14, 6127−6138). Given our computational resources and the nature of the specific research question related to loop dynamics, we opted for the first strategy, as now explained in the Methods section. As regards replicas, it is customary to either (i) assign different initial atomic velocities using different random seeds and/or heating protocols or (ii) start the simulation with slightly altered initial structures. This latter alternative was used in the present case as it allowed a reasonably broad sampling of the phase space in the activation loop and αC helix regions. In addition to using the RCD+ web server for loop remodeling, we employed as alternative initial structures those generated by AlphaFold and by SwissModel. We have updated the Methods section and the details of this modelisation are described in the new Supplementary Table 5 and Supplementary Figure 6. The outcome of these simulations was a manifold of conformations that covered the whole spectrum from inactive to active states. We provide a new movie (Supplementary Video 5) and a new figure (Figure 4d) to cover the issues put forward by the reviewers, namely (i) the conformational motions driving the activation process, (ii) the orientation of the αC helix, and (iii) the formation/disruption of the catalytically essential K42–E61 salt bridge.

Major Points
1. In the Methods section, only the MD simulation of the StkP^{287}-2TA system in complex with AMP-PNP and Mg^{2+} is described, whereas StkP^{287}-WT, StkP^{287}-2pT, and StkP^{287}-2TE were also

simulated. The modeling procedures for these systems should be clarified in detail. It would be advisable to include a table listing, for each simulated system, the total number of atoms (protein, solvent, and ions), and the length of each trajectory.
The Methods description has been rewritten and a table (Supplementary Table 5) together with a new figure (Supplementary Figure 6) now provide the necessary details about the molecular dynamics simulations, as requested.

2. The presentation of the MD results is limited. Only a single trajectory plot is provided (Fig. 4a), showing the RMSFs of the system referred to as "StkP^{287}-2T" in the figure legend. Were 300 ns sufficient to ensure convergence of the systems? How much did the simulated systems deviate from their initial states during the course of the trajectories? Furthermore, monitoring key interatomic distances over the simulations would strengthen the description of conformational motions.
The trajectory plot in this figure is representative of the whole set of initial simulations as it clearly highlights the most flexible regions, which correspond very accurately to the sequence stretches that are awarded the lowest confidence values by tools like AlphaFold or Swiss-Model, precisely because they can adopt a variety of conformations. This is particularly true for the pleomorphic activation loop, which in the absence of threonine phosphorylation displays the highest flexibility. As we also perfomed the simulations of StkP287-2TA and StkP287-2TE, we now provide in Figure 4 (Figure 4abc) three panels showing the RMSFs of StkP^{287}-2T, StkP287-2TA and StkP287-2TE.

As regards deviation from the starting structure over time, it is popular to plot the rmsd vs ns to identify large changes in overall protein structure relative to the starting point (or sometimes the final or average structure) as an easy means to interpret global stability (i.e., positional differences between entire structures over time). However, a plateau may only indicate that the simulation is stuck in a local minimum but not the global, functional minimum. Convergence and stability are not at stake here because they guaranteed, as can be visually apprehended by watching the movies. We believe it is far more valuable (and visually comprehensible) to analyze the flexibility of individual residues during each simulation (i.e., how much particular residues fluctuate over time). In our view, reporting this behavior for the whole set of simulations as a list of distances would be very cumbersome to read and understand.

3. The simulations were conducted over a relatively short timescale, limiting the robustness of the conclusions. For each system, at least three independent replicas should be performed, and the corresponding analyses reported.
It must be borne in mind that the goal of our initial simulations starting from the X-ray crystal structure(s) was to sample the conformational behavior of the least well-defined regions r. Furthermore, three independent replicas of each system were simulated, as now more clearly explained in the Methods section and the Supplementary Table 5.

4. The three highest-scoring conformations of the activation loop generated by the RCD+ server were used as independent replicas for the StkP^{287}-2TA simulation. However, it should be noted that the RCD+ algorithm is optimized for loop lengths of up to 12 residues. A comment on the rationale for using RCD+ in this context would be appropriate.
Please refer to the general comments above and to the newly written sentence in the Methods section "The RCD+ server was used to generate alternative conformations of the incomplete activation loop (amino acids 164-174) and select the three top-scoring ones as system replicates with distinct initial configurations."

5. It is well established that AlphaFold3 performs well in modeling structured regions, but is less reliable for intrinsically disordered regions. Since the authors note that the degree of confidence is lower for the activation loop than for the rest of the protein, Extended Data Fig.4 should include the predicted local distance difference test (pLDDT) scores for the AlphaFold3 models. Moreover, the discussion regarding the orientation of the αC-helix and the K42–E61 salt bridge, based on the AlphaFold3 model, would be more robust if supported by a broader panel of MD simulations. Despite their computational cost, MD simulations have been widely applied to investigate conformational transitions in kinases.
Please refer to the General comments above, the Supplementary Table 5 reporting the MD simulations and the new Supplementary Figure 6 that show the requested metrics for both the AlphaFold models and the homology model built by Swiss-Model.

6. Figure 4. The authors should clarify which representative MD snapshot is shown, and on what basis it was selected.
We have modified Figure 4 to answer the other concerns and have updated the legend accordingly to clarify what is shown.

Minor Points

1. There are few inaccuracies in the legend of Extended Data Figure 4.
The Extended Data Figure 4 is now Supplementary Fig. 5 and its legend has been improved.

2. Correct ANP.
ANP is the correct term and corresponds to AMP-PNP according to the WWPDB Chemical Component Dictionary. It is now indicated in the text.

3. The x-axis label is missing in Fig. 4a.
Corrected

Reviewer #3 (Remarks to the Author):

This study reports the structural analysis of the catalytic domain of the Hanks protein kinase StkP from S. pneumoniae and examines how the phosphorylation of the activation loop and the dimerization of the catalytic domain contribute to its activation. This is a solid work combining X-ray crystallography with biophysical methods, molecular dynamics simulations, in vitro and in vivo mutational analysis and cell imaging. The data support the two major conclusions of the manuscript: firstly, that the phosphorylation of the activation loop regulates activity by influencing the loop conformation and dynamics; and secondly, that kinase activation in vivo is driven by concentration-dependent dimerization of the catalytic domain, indicating that the binding of peptidoglycan fragments or precursors to PASTA domains is not essential for StkP activation. This study is based on well-designed experiments, and the results are of importance to the field of bacterial signaling. However, there are some points (detailed below) that must be addressed by the authors prior to publication.
We thank the referee for the laudatory comments about our work and we provide below our answers to his/her comments.

The authors state in the introduction that the current model involves peptidoglycan-induced PASTA dimerization (as shown schematically in Fig.1a) acting as signaling domains to trigger kinase activation, and refer to this model as "a conventional ligand-induced activation paradigm of PASTA-Hanks kinases" (page 20, line 6). However, if extracellular ligand-induced dimerization could be considered a paradigm for eukaryotic receptor kinases, it is clearly not a paradigm for bacterial transmembrane kinases. Particularly in the case of PASTA kinases, ligand-induced dimerization is just a highly speculative hypothesis lacking experimental support that, to the best of my knowledge, was put forward by Barthe et al (Structure, 18, 606-615, 2010) based on a vague homology of PASTA kinases with the unrelated structure of PASTA-containing PBP2X (Pares et al, Nat Struct Biol, 3, 284-289, 1996). In contrast, recent studies from various laboratories, including the authors' own, have cast significant doubt on this interpretation and even questioned the function of PASTA subunits as signaling domains. The current knowledge about the possible roles of PASTA domains in StkP activity needs to be presented in a more balanced manner, and Fig. 1a should be modified accordingly.
We agree with the referee. The sentence has been revised (page 20, line 15) to clarify this point. We also now indicate in the introduction that this model remains an highly debated area of research (page 3, line 26). We think that modifying Fig. 1a would be a challenging undertaking as it corresponds to the model currently proposed. Consequently, a second question mark has been incorporated to emphasize the ongoing ambiguity surrounding both ligand binding and the activation process by dimerization. In conjunction with Fig. 7, which presents the alternative model based on our observations, and in which the activation trigger may be cytoplasmic and PASTA domains are not involved, we think that it represents an appropriate way to present the activation of PASTA-Hanks kinases in a balanced manner.

The molecular dynamics simulations described in page 10 indicate that the activation loop was highly flexible in the 2pT and 2TE mutants, in agreement with the crystal structures, but remained in a conformation obstructing the active site in the wt and the 2TA mutant. As this is a central result of the manuscript, these mobility differences should be shown in Figure 4, possibly by modifying panel 4A.
We agree and have now updated Figure 4. New RMSFs are shown for StkP287-2TA and StkP287-2TE in panels b and c and a new figure 4d is provided to clarify this point.

The crystal structures revealed a novel, asymmetric dimerization mode of the catalytic domain, involving contacts between the N-lobe of one molecule and the C-lobe of the second molecule (the NC interface), which maintains the kinase in an inactive conformation. This NC interface differs from the back-to-back (NN) and front-to-front (CC) dimerization modes observed for M. tuberculosis PknB. Importantly, the AUC experiments (Figs. 5d-g) showed that the NC dimers seen in the crystals are also present in solution. This, together with the conservation of the dimeric interface in various Streptococcus species, suggest a putative physiological role of these NC StkP dimers. Such conservation is difficult to see in the multiple alignment (Extended Data Fig. 5) and should be better shown by mapping the conserved residues on the molecular surface of StkP.

We agree with the referee and have included a new panel in Supplementary Fig. 7 (previously Extended Data Fig. 5) showing the mapping of conserved residues on the molecular surface of StkP. The figure legend was updated accordingly.

The analysis of the phosphorylation pattern, growth and cell morphology of three StkP mutated pneumococcal strains, respectively unable to form NC, NN or CC dimers, provided important information on the biological relevance of the dimerization interfaces. The N*N and C*C mutants, but not the N*C mutant, exhibited significantly altered phosphorylation patterns and impaired growth, consistent with those of a dead-kinase mutant strain. These results are relevant, as they suggest that the CC and N-N dimers are important for kinase activation, in a similar way as proposed for PknB. However, neither of these complexes could be detected by AUC in vitro, not even for the N*C mutant at the highest tested concentration of 10 mg/ml (Fig. 5e). While I can understand this for the CC complex (as it possibly corresponds to the transient autophosphorylation complex), it is more difficult to see why the N-N dimer could not be detected.

We fully agree with the referee and were also surprised that the NN dimer was not detected. Our hypothesis is that other structural features of StkP are required for the NN dimerization process *in viv,* including its juxtamembrane cytoplasmic domain and/or the transmembrane segment. It is also possible that other cellular components may contribute to the stabilization of the NN interface *in vivo*. Resolving this issue is clearly needed and will be a primary focus in future investigations.

As the N*C mutant strain exhibit a normal phosphorylation pattern in vivo, the authors suggest that these dimers may play a non-catalytic role. They propose a revised model for StkP activation according to which NC dimerization represents a resting inactive state to prevent premature activation, and further interaction with a (so far unidentified) cellular partner may be required to trigger the transition into the active NN dimer. However, some difficulties with this model are that cell growth and morphologies of the N*C mutant strain are wild-type-like, suggesting that NC dimerization would not be required for normal cell division in vivo, and that the StkP NN dimers could not be detected in vitro, as noted above. Could it be possible that the mutations of the putative NN interface could affect kinase activity by a mechanism other than precluding NN dimerization?

We don't think so. As stated in our previous response, it is also possible that the juxtamembrane cytoplasmic domain establishes interaction with the back side of the kinase domain through the same interface that is used for NN dimerization. Addressing these issues will require further experiments to gain a comprehensive understanding of StkP activation.

In page 15, lines 1-3, the authors state that "the absence of an elongated phenotype for the C*C mutant strain suggests that mutating this interface suppresses the need of StkP kinase activity for cell division", implying that kinase activity is normally required for cell division. However,

the same chain-like morphological phenotype seen for the C*C strain was also observed for the ΔstpK mutant strain (Fig. 1e). As the C*C mutation is expected to prevent formation of the autophosphorylation complex, one could think from the above phenotypes that kinase activity would not be required for cell division. Could the authors comment on this?
Cell division defects results in different phenotypes, incuding aberrant cell elongation, cell rounding and/or cell chaining. As previously reported (Fleurie et al., 20212, Mol. Microbiol.), the deletion of *stkp* leads to cell rounding and chaining whereas kinase inactivation with the K42M mutation leads to cell elongation. This means that beyond its kinase activity, StkP likely fullfil other functions by establishing interactions with other components that are required for efficient cell division. This is notably the case for the cell division proteins LytB (Martinez-Carballero et al., 2023, Cell Rep.) and PBP2x (Morlot et al., 20212, Mol. Microbiol.). The observation that both the deletion of *stkP* and expression of the C*C form display cell rounding and chaining suggest that this phenotype is due not only to an absence of kinase activity but also to an impaired ability to interact with some partners. On this basis, it can be speculated that the C*C mutation also affects the organization of StkP to allow optimal interaction with some partners. This conclusion is however very speculative and we don't think it deserve to be presented in the manuscript.

In page 8, lines 24-26, the authors state that "stabilization of the activation loop resulted from the addition of AMP-PNP and cations, rather than crystal packing". However, the inspection of the highest resolution structure suggests the opposite conclusion, namely that stabilization resulted primarily from crystal contacts. Indeed, the ordered loop in the first independent molecule is engaged in intermolecular contacts with two other StkP molecules in the crystal, whereas the same loop in the second independent molecule (which obviously binds the same nucleotide and cations) is less engaged in crystal contacts and is structurally disordered.
We agree with the referee that the activation loop of molecule B in the asymmetric unit is more extensively engaged in intermolecular crystal contacts than in molecule A, and that this correlates with its increased ordering. However, the $StkP^{344}$–2TA structure was obtained in the same space group and with essentially identical crystal packing, yet the activation loop is not visible in any molecule of the asymmetric unit. The main difference between these two structures is the shorter length of the construct and the presence of AMP-PNP and cations. We therefore suggest that ligand binding primarily promotes stabilization of the activation loop, while crystal contacts may further enhance its ordering in one molecule of the asymmetric unit.

In Page 10, lines 22-26, the authors describe the G174P mutation, which they designed to investigate the flexibility of the activation loop. However, in the crystal structure Gly174 adopts a main-chain conformation that is not compatible with a proline residue, and the substitution is therefore introducing conformational changes. I wonder whether these changes, rather than the decreased mobility, could account for the observed growth effects (Fig. 4d). The authors should comment about this.
We thank the reviewer for raising this point. To address whether the G174P substitution would modify backbone conformation, we modeled the G174P mutation into the StkP287-TA crystal structure and performed local refinement. Although proline imposes additional conformational constraints, this analysis indicates that the main-chain conformation can be accommodated without major structural rearrangements at this position.
That said, we agree that we cannot fully exclude the possibility that the effects observed in Supplementary Fig. 4 arise not only from reduced loop flexibility but also from more subtle perturbations introduced by the mutation. In particular, Gly174 may contribute not only to activation-loop mobility but also to backbone-mediated interactions involved in substrate recognition or binding, which could be affected by the proline substitution. We have therefore

revised the manuscript to clarify that while the G174P mutation was designed to reduce flexibility, additional structural or functional effects may also contribute to the observed growth phenotype (page 11, lines 3-9).

In Page 10, lines 7-8, the authors state that this is the first reported bacterial Hank-kinase structure in which the activation loop is fully resolved. This is not the case, as for instance the structure of M. tuberculosis PknG also has a well-defined activation loop in the crystal structures (Scherr et al, PNAS 104, 12151, 2007; Lisa et al, Structure 23, 1039, 2015).
We agree with the referee and indeed meant that this is the first reported PASTA-Hanks kinase for which the activation loop is fully resolved. We have revised the sentence on page 10, line 7-9, to clarify this point. The revised text now reads: "Although fully resolved activation loops have previously been reported for bacterial Hanks kinases, for example in the *Mycobacterium tuberculosis* PknG structures (Scherr *et al.*, PNAS 104, 12151, 2007; Lisa *et al.*, *Structure* 23, 1039, 2015), this represents the first reported bacterial PASTA-Hanks kinase structure in which the activation loop is fully resolved."

In page 17, line 8, change 'cognate phosphate' for 'cognate phosphatase'.
Corrected.

In page 17, lines 15-16: Figure 4 does not show the differences in loop mobility between the 2TA and 2TE mutants (see comment above on Figure 4).
As stated above, we have now updated Figure 4. New RMSFs are shown for StkP287-2TA and StkP287-2TE in panels b and c and a new figure 4d is provided to clarify this point.

The legend to Fig. 4C is missing.
We apologize for this omission. The Fig 4c is now refered as Fig. 4 f and g and the legend has now been added.

Reviewer #1
The authors have satisfactorily addressed my comments and suggestions
We thank the reviewer for his/her positive feedback.

Reviewer #2
Dear Authors,
Thank you for your thorough and well-organized responses to the comments. I am satisfied that the concerns I raised have been carefully considered and addressed.
We thank the reviewer for appreciating our changes to the manuscript.

Reviewer #3
I believe that the authors have addressed most of my concerns and those from the other reviewers in the revised version. The toned down interpretations make for a more balanced discussion, even if I'm still not fully convinced that, based on the crystallographic data alone, one could conclude that stabilization of the activation loop is primarily due to nucleotide and cation binding.
We thank the reviewer for his/her positive comment. Primary stabilization of the activation loop by nucleotide and cation binding is a hypothesis and we don't exclude that other events may contribute, as stated in the manuscript.
