# Supplementary material for: Structural dissection of the catalytic domain of the serine threonine kinase StkP of Streptococcus pneumoniae
Source: Nat Commun. 2026 Jun 18;17:7687. doi: 10.1038/s41467-026-74470-6 (PMC13434768; doi:10.1038/s41467-026-74470-6)

Electron density map of residues 42-63 (active site) from the 4 crystal structures : StkP^{344}-2TA, StkP^{287}-2TA (Mg), StkP^{287}-2TA (Mn) and StkP^{287}-2TE . Cartoons, main chain; sticks, side chains and AMP-PNP; grey mesh, 1-s contour of the 2F o – F c electron density map.

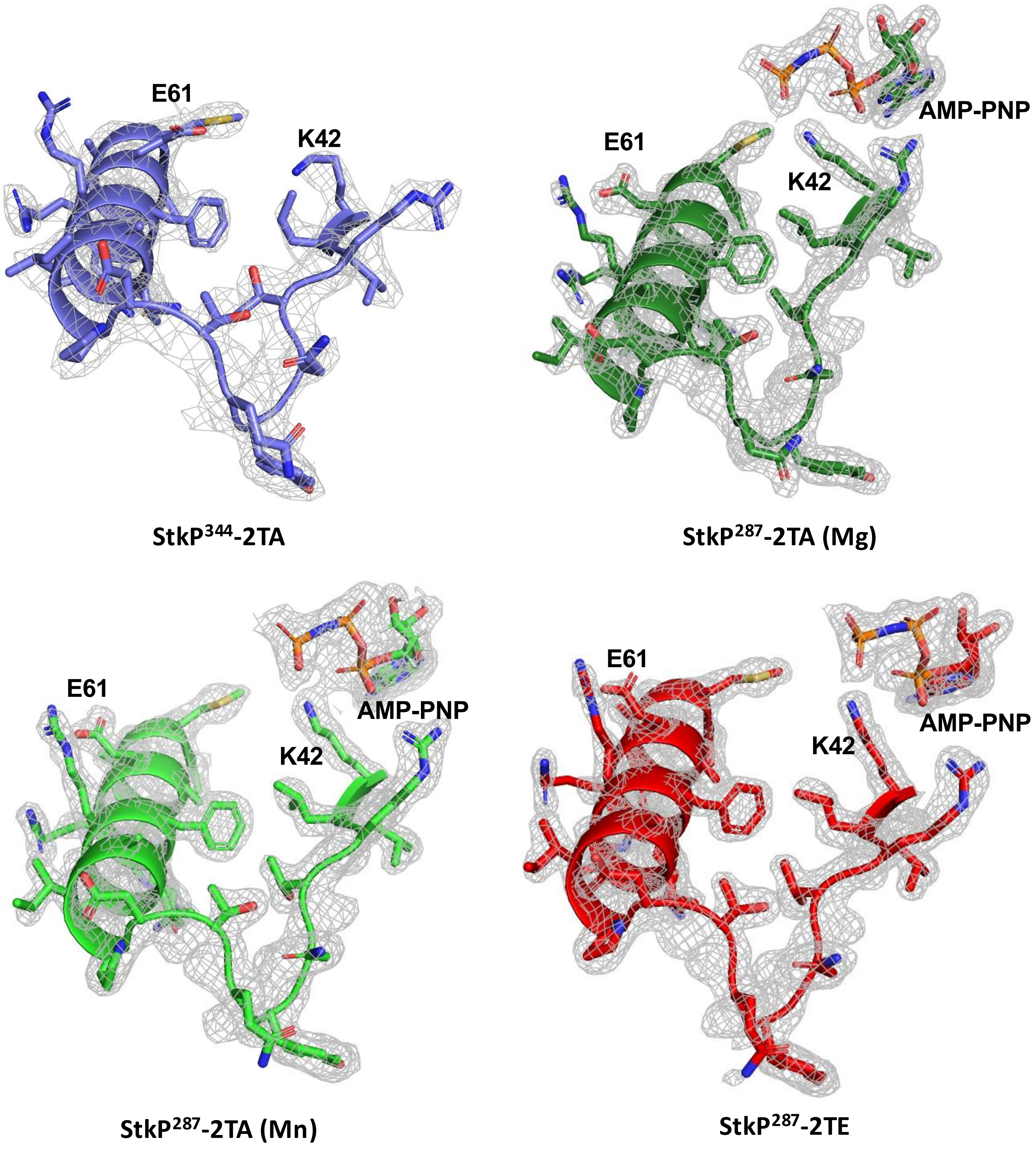

Supplement: Supplementary file 5 [file 41467_2026_74470_MOESM10_ESM.zip › SourceData/SourceData-electron-density-maps-Fig2and4-SuppFig3and5 copie.pdf]
