# Supplementary material for: Structural dissection of the catalytic domain of the serine threonine kinase StkP of Streptococcus pneumoniae
Source: Nat Commun. 2026 Jun 18;17:7687. doi: 10.1038/s41467-026-74470-6 (PMC13434768; doi:10.1038/s41467-026-74470-6)

Fig. 1b

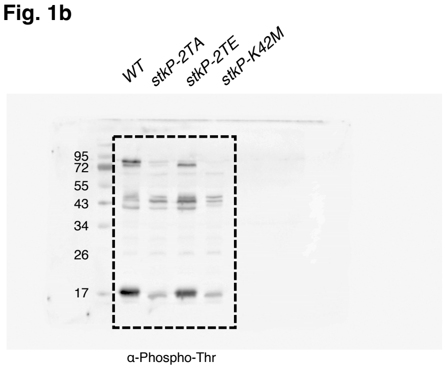

Fig. 3a

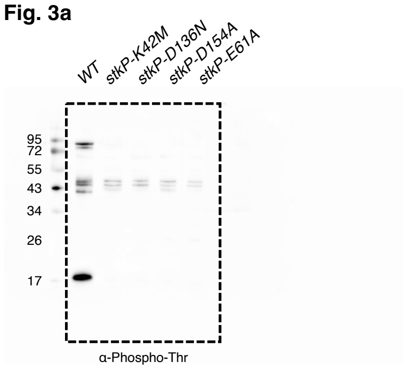

Fig. 6a

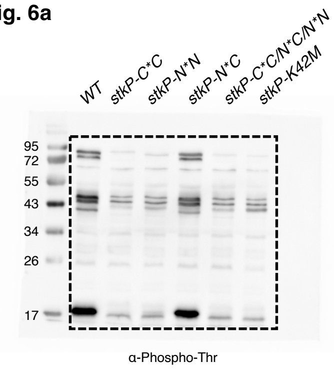

Supplement: Supplementary file 5 [file 41467_2026_74470_MOESM10_ESM.zip › SourceData/SourceData-WesternBlot-Fig1b-3a-6a copie.pdf]
